# The eIF3 complex of *Leishmania*—subunit composition and mode of recruitment to different cap-binding complexes

**DOI:** 10.1093/nar/gkv564

**Published:** 2015-06-19

**Authors:** Shimi Meleppattu, Dikla Kamus-Elimeleh, Alexandra Zinoviev, Shahar Cohen-Mor, Irit Orr, Michal Shapira

**Affiliations:** Department of Life Sciences, Ben-Gurion University of the Negev, Beer Sheva, Israel

## Abstract

Eukaryotic initiation factor 3 (eIF3) is a multi-protein complex and a key participant in the assembly of the translation initiation machinery. In mammals, eIF3 comprises 13 subunits, most of which are characterized by conserved structural domains. The trypanosomatid eIF3 subunits are poorly conserved. Here, we identify 12 subunits that comprise the *Leishmania* eIF3 complex (LeishIF3a-l) by combining bioinformatics with affinity purification and mass spectrometry analyses. These results highlight the strong association of LeishIF3 with LeishIF1, LeishIF2 and LeishIF5, suggesting the existence of a multi-factor complex. In trypanosomatids, the translation machinery is tightly regulated in the different life stages of these organisms as part of their adaptation and survival in changing environments. We, therefore, addressed the mechanism by which LeishIF3 is recruited to different mRNA cap-binding complexes. A direct interaction was observed *in vitro* between the fully assembled LeishIF3 complex and recombinant LeishIF4G3, the canonical scaffolding protein of the cap-binding complex in *Leishmania* promastigotes. We further highlight a novel interaction between the C-terminus of LeishIF3a and LeishIF4E1, the only cap-binding protein that efficiently binds the cap structure under heat shock conditions, anchoring a complex that is deficient of any MIF4G-based scaffolding subunit.

## INTRODUCTION

Translation initiation in eukaryotes is a highly regulated process that requires the coordinated action of a large number of proteins. The process begins with the binding of the ternary complex (TC, eIF2-GTP-Met-tRNA^met^) to the 40S ribosomal subunit, resulting in the formation of the 43S pre-initiation complex (PIC) (1). The PIC then interacts with the 5’ end of the mRNA with the help of the eukaryotic initiation factor 4F (eIF4F) to form the 48S PIC (2). eIF4F consists of a cap-binding protein, eIF4E, an RNA helicase, eIF4A and a scaffolding protein eIF4G, which in most cases is responsible for the recruitment of eIF3 and its associated ribosome to the mRNA (3). The initiation complex scans the mRNA until it reaches the initiation codon, where the 60S subunit joins, and the assembled 80S ribosome enters the elongation stage (1). eIF3 plays a major role in almost all the steps in this complex process, serving as a ‘versatile scaffold’ and coordinating the activity of the initiation factors involved (4,5,6,7,8). The diversity of eIF3 function is also reflected in its involvement in coupling transcription and translation (9), mRNA export (10), non-sense-mediated mRNA decay (11) and anti-viral defense mechanisms (12,13). In addition, eIF3 is also engaged in the re-initiation of specific transcripts under discrete conditions (14,15). eIF3 subunits were identified as targets for mTOR and SK6 signaling pathways, thereby providing a platform for translation regulation in response to various stresses (16). Finally, the altered regulation of eIF3 subunit expression has been implicated in the development and progression of cancer (17,18).

Our knowledge of eIF3 structure and function originates mainly from studies in yeast and mammals. The mammalian eIF3 is a large complex (∼800 kDa), comprising 13 subunits, eIF3a-eIF3m (8). Budding yeast eIF3, on the other hand, is much simpler, with only five stoichiometric subunits, orthologs of the mammalian a, b, c, g and i subunits, and a sixth, sub-stoichiometric subunit, an ortholog of subunit j. The five stoichiometric subunits of yeast eIF3 are often referred to as the ‘conserved core’ of eIF3, as these subunits are sufficient for performing ‘core’ eIF3 functions (19). Cryo-EM reconstruction studies of the human eIF3 complex proposed a stable structural core consisting of subunits a (amino acids 5–654) and c (amino acids 302–913), along with subunits e, f, h, k, l and m (20). These eight eIF3 subunits share a high degree of sequence and structural conservation with components of the proteasome lid and the COP9 signalosome, together known as PCI (Proteasome, COP9/Signalosome, eIF3) complexes. Each PCI complex contains six PCI-domain subunits (in eIF3, these are subunits a, c, e, k, l and m) and two MPN (Mpr1p and Pad1p N-termini) domain-containing subunits (in eIF3, these are subunits f and h) (21). Among the PCI complexes, only eIF3 includes an additional five non-PCI/MPN-containing subunits (b, d, g, i and j). eIF3 subunits b and g are known to harbor an RNA recognition motif (RRM) (22,23). The PCI-MPN core of the mammalian eIF3 engages with the 40S ribosomal subunit in the same manner as does the eIF3a-eIF3c PCI heterodimer in yeast, while a non-PCI module involving part of eIF3b, along with subunits g and i, is positioned somewhat apart from the PCI-MPN core in both systems. The strategic positioning of these sub-complexes allows eIF3 to completely encircle the 40S subunit and interact with its other binding partners (19). The ‘m’ subunit can dissociate from eIF3 under unfavorable conditions, suggesting that it resides on the surface of the complex (24). Subunit j is loosely associated with the eIF3 complex, where it is found in sub-stoichiometric amounts, although it stabilizes the binding of eIF3 to 40S ribosomal subunits (23,^25^).

The interaction between eIF3 and eIF4G, the scaffold subunit of the eIF4F complex, assists in the binding of the 40S ribosomal subunit to mRNA in mammals (3,26). This interaction is a critical step in the initiation of cap-dependent translation. Pathogenic trypanosomatids, such as *Leishmania*, possess an expanding number of eIF4E and eIF4G variants. There are six isoforms identified for eIF4E and at least five isoforms of eIF4G in these organisms(27,^28^,^29^,^30^,^31^,^32^). Different eIF4E orthologs were shown to possess altered cap-binding properties, as well as variations with respect to their eIF4G partners (28,33,34,35). Cap-dependent translation is known to be highly sensitive to a variety of stresses. Under such conditions, translation of specific mRNAs proceeds through diverse mechanisms, including cap-independent pathways. For digenetic parasites, such as *Leishmania*, which cycle between invertebrate vectors and mammalian hosts, these regulatory mechanisms are highly significant, as they must adapt to extreme environmental changes during the organism's life cycle. Altered profiles of gene expression lay the foundation for differentiation processes, with translation regulation playing a key role (36,37).

The canonical cap-binding complex of *Leishmania* is believed to consist of LeishIF4E4, LeishIF4G3 and LeishIF4A1 (29,32). However, this complex was shown to be unstable at temperatures typical of the mammalian host, when the LeishIF4E4-LeishIF4G3 interaction is interrupted and LeishIF4E4 loses its ability to bind the cap structure (35). Under these conditions, another cap-binding protein, LeishIF4E1, comes into play as it retains the ability to bind the cap structure even at such conditions. The role of LeishIF4E1 is not clear yet, although it is known to bind the cap structure very efficiently at all temperatures and associates with a multitude of translation initiation factors, apart from any eIF4G ortholog (35).

In the context of different cap-binding complexes that are observed in *Leishmania*, the mechanisms by which these organisms recruit the 40S ribosomes to the mRNA cap are rather obscure. Here, we describe a biochemical analysis of the *Leishmania* IF3 complex, supported by a bioinformatics analysis, and address the versatile mechanisms by which the complex is recruited to the different cap-binding complexes which prevail during the parasite's life cycle. We show that while LeishIF3 is recruited to the canonical LeishIF4E4-based cap-binding complex through an eIF4G ortholog, LeishIF3 shows an unusual mode of recruitment to the LeishIF4E1 complex, based on its direct binding to LeishIF4E1, in the absence of any eIF4G-like hub.

## MATERIALS AND METHODS

### Organisms

*L. amazonensis* promastigotes were cultured in Medium 199 (pH 7) supplemented with 10% fetal calf serum (FCS), 5 μg/mL hemin, 0.1 mM adenine, 40 mM Hepes (pH 7.4), 4 mM L-glutamine, 100 U/mL penicillin and 100 μg/mL streptomycin at 25°C.

### Database searches and homology studies

The TriTryp genome database (www.tritrypdb.org/) was scanned for potential homologs of eIF3 subunits by the BLASTp facility of the database. The query sequences were taken from HomoloGene (http://www.ncbi.nlm.nih.gov/homologene) or Uniprot (http://www.uniprot.org). To identify proteins with very low conservation, sequences of interest served as queries in searches of the NCBI *Leishmania* database using the PsiBLAST server (http://www.ncbi.nlm.nih.gov/blast/). Hits identified were further used as query sequences in BLASTP searches against the NCBI genome database. Additionally, any sequences that were thus identified were analyzed by HHpred (toolkit.tuebingen.mpg.de/hhpred) to ensure homology. The presence of signature domains in the subunits was verified by SMART (smart.embl-heidelberg.de/) and Pfam (http://pfam.xfam.org/search) searches, as well as by HHpred analysis. Secondary structure predictions were carried out using psipred (bioinf.cs.ucl.ac.uk/psipred/). Multiple sequence alignments were performed using the ClustalW server (www.ch.embnet.org/software/ClustalW.htm).

### Plasmids for generating transgenic parasites

Polymerase chain reaction (PCR)-amplified LeishIF3e, LeishIF3a and LeishIF4G3 genes were cloned into the BamHI and Xba1 sites of the pX H-SBP-H plasmid cassette(35) which introduces a C-terminal streptavidin-binding peptide (SBP) tag (for primer sequence, see Supplemental Table S1). *L. amazonensis* cells were transfected as previously described(35) with the different pX-derived transfection vectors harboring translation initiation factors fused to SBP, under control of HSP83 intergenic regions (H), and stable cell lines were selected using 200 μg/ml G-418. For expression of recombinant LeishIF4G3 and LeishIF4E4 in bacteria, the open reading frames of each gene were cloned into plasmid pGST(38) . A cell line that overexpressed untagged LeishIF4E1 was generated by transfection of the pX-H-LeishIF4E1-H plasmid (35). LeishIF4E1 expression was, therefore, driven by upstream and downstream intergenic regions derived from the HSP83 genomic cluster.

### *In vivo* pull-down analysis

LeishIF3e, LeishIF3a, LeishIF4G3 and LeishIF4E4 were expressed in *Leishmania* as transgenic fusion proteins bearing SBP tags. For pull-down assays, transgenic cells (0.5–1×10^9^) were harvested, washed twice in PBS (pH 7.4), once in PRS (35 mM HEPES (pH 7.4), 100 mM KCl, 10 mM MgCl_2_, 1 mM DTT) and lysed in PRS+, consisting of PRS buffer supplemented with a 1X cocktail of protease inhibitors (Sigma), 20 mM iodoacetamide, 20 mM NaF, 50 mM β glycerophosphate and 1% Triton X-100. The cells were incubated on ice for 5 min, centrifuged for 20 min at 20 000 g and the supernatant was incubated with streptavidin-Sepharose beads for 2 h at 4°C with constant shaking. The beads were washed with PRS+ containing 1% NP40, four times. The bound protein complex was eluted by incubation in PRS+ containing 5 mM biotin. A control pull-down assay was carried out with cells transfected with a luciferase gene that was expressed as a SBP-tagged fusion protein as described above. The pull-down experiments were repeated at least three times to ensure reproducibility.

### Mass spectrometric analysis

The eluted protein complex was precipitated by trichloroacetic acid (TCA) and separated over 12% sodium dodecyl sulphate (SDS) polyacrylamide gels. The gel was stained by Coomassie blue and the stained lane was divided into three segments. The proteins in each gel segment were digested by trypsin, analyzed by LC-MS/MS using an LTQ-Orbitrap (Thermo) and the resulting peptides were identified by Discoverer software, that was compared with the *L. major* version 7 listing obtained from the TriTryp database, or a decoy database (to determine the false discovery rate). All identified peptides were filtered with high confidence, top rank, mass accuracy and a minimum coverage of 2 peptides per protein was determined. High confidence peptides exceeded a 1% false discovery rate, namely the estimated fraction of false positives in a list of peptides. Semi-quantitation of the abundance of each protein was deduced from the peak area, as well as by the protein abundance factor (PAF) values, corresponding to the spectral count normalized to the molecular weight of the protein. The area of the protein represents the average of the three most intense peptides from each protein.

### Monitoring the *in vitro* interaction between the LeishIF3 complex and recombinant LeishIF4G3, LeishIF4E4 and LeishIF4E1

LeishIF4G3 and LeishIF4E4 were expressed in bacteria as fusion proteins bearing a GST tag at their N-terminus, using the pGST-parallel expression vector, whereas LeishIF4E1 was expressed from the pHIS-parallel expression vector (38). A bacterial culture (100 ml) of strains expressing these proteins was lysed in PRS buffer (10 ml) containing protease inhibitors, using a French press pressure cell. Following cell disruption, the bacterial extract was centrifuged for 20 min at 20 000 g. The LeishIF3 complex from a late-log culture of transgenic cells expressing the SBP-tagged LeishIF3e [100 ml (10^7^/ml)]. Following lysis with 1% Triton X-100 and centrifugation at 20 000 g at 4°C for 20 min, the mixture was bound to streptavidin-Sepharose beads via the SBP-tagged LeishIF3e subunit as described earlier, washed three times with PRS+ buffer lacking NP40. The beads with bound eIF3 complex were then re-suspended in 1 ml PRS+ buffer containing 20 μl of supernatant obtained from the bacterial cells expressing recombinant LeishIF4G3, LeishIF4E4 or LeishIF4E1 (∼0.2–0.8 μg of recombinant protein) and incubated for 1 h with constant shaking. The beads were further washed (X5) with PRS+ containing 0.1% NP40 and the bound complex was subsequently eluted with 1 ml of 5 mM biotin in PRS+. The eluted complex was further resolved over 12% sodium dodecyl sulphate-polyacrylamide gel electrophoresis (SDS PAGE), blotted and reacted with antibodies against LeishIF4G3, LeishIF4E4, LeishIF4E1 or SBP. A parallel control pull-down was performed with SBP-tagged luciferase expressed in transgenic *L. amazonensis* cells. The cell lysate of the luciferase-SBP cell lines were incubated with streptavidin-Sepharose beads, washed with PRS+ and further incubated with recombinant LeishIF4E1 or with recombinant LeishIF4G3. The bound complex was eluted and analyzed as described above.

### Monitoring the interaction between the LeishIF3 complex and overexpressed endogenous LeishIF4E1 *in vitro*

To verify the interaction between LeishIF4E1 and LeishIF3, a pull-down experiment was carried out by lysing a mixture of whole cells from two transgenic cell lines, one expressing SBP-tagged LeishIF3a and the other expressing non-tagged LeishIF4E1 from the pX-H-LeishIF4E1-H plasmid described above. The cells were mixed at a 10:1 ratio (of cell counts), the mixture was lysed by the addition of 1% Triton X-100, followed by centrifugation at 20 000 g at 4°C for 20 min. LeishIF3a-interacting proteins were purified over streptavidin-Sepharose beads, as described above. The presence of LeishIF4E1 in the elution was demonstrated with antibodies raised against LeishIF4E1 in an immunoblot protocol. A control experiment was carried out by mixing cell lines expressing SBP-tagged luciferase and non-tagged LeishIF4E1.

### Yeast two-hybrid assays

A yeast two-hybrid assay was performed using a commercial GAL4-based Two-Hybrid Phagemid Vector Kit (Stratagene), following the manufacturer's instructions. The LeishIF4E1 ORF was cloned into the GAL4-binding domain vector (pBD) using the EcoRI and Sal I sites. Full length and truncated versions of LeishIF3a were cloned into the pAD yeast vector expressing a GAL4-activation domain, using the EcoRI site. The eIF3a fragments corresponded to positions 1–210, 211–400, 401–536, 537–774. Two additional overlapping fragments were also generated, corresponding to positions 1–400 and 211–536. The yeast strain YRG-2 (Mata ura352 his3-200 ade2-101 lys2-801 trp1-901 leu2-3 112 gal4-542 gal80-538 LYS2::UASGAL1-TATA GAL1-HIS3RA3::UASGAL4 17mers(x3) TATACYC1-lacZ) was co-transformed with plasmids pBD-LeishIF4E1 and pAD-LeishIF3a. A reciprocal assay was also performed with plasmids pAD-LeishIF4E1 and pBD-LeishIF3a. Assays that tested the interaction between LeishIF4E1 and the LeishIF3a fragments were done with the pBD-LeishIF4E1 and pAD-LeishIF3a-fragment plasmids. The transformed yeast were cultured in liquid SD-2 (-Trp/-Leu) medium at 30°C overnight, diluted to a final concentration of OD_600_ of 0.15 and grown to OD_600_ of 0.5. The yeast were spotted in X3 fold dilutions on SD-2 (-Trp/-Leu) and SD-3 (-Trp/-Leu/-His) plates. Empty pAD and pBD plasmids, or plasmids encoding a non-relevant protein, served as negative controls, following the manufacturer's instructions. To address LeishIF4E1-LeishIF3a fragment interactions, yeast transformants were plated on SD-2 (-Trp/-Leu) and SD-3 (-Trp/-Leu/-His) plates.

For demonstrating the interaction between subunits a and c of LeishIF3, their ORFs were amplified from genomic DNA using gene-specific primers (Supplemental Table S1). PCR-amplified LeishIF3a was cloned into the BamHI and XbaI sites of the pAD yeast vector and LeishIF3c was cloned into the EcoRI and SalI sites of the pBD yeast vector. The two plasmids were used in the yeast two-hybrid system described above, with the SD-2 and SD-3 plates containing 1.75 mM 3-amino-1,2,4-triazole (3AT). Growth in the absence of histidine, indicating interaction of the LeishIF3a and LeishIF3c subunits, was monitored after 4–7 days.

Expression of the proteins tested in the yeast two-hybrid assay was monitored by western blot analysis. Log phase yeast cultures (OD_600_ = 1.0) were harvested, and precipitated in 200 μl 20% TCA. Glass beads were added and the cells were disrupted by vortexing X3 for 5 min each. The glass beads were discarded and the lysate was centrifuged at 10 000 g for 10 min. The pellet was re-suspended in Laemmli sample buffer, boiled for 8 min, centrifuged for an additional 2 min at 13 000 g and equal protein quantities were loaded onto SDS-polyacrylamide gels (12%). The gels were blotted and reacted with specific antibodies against the AD and BD domains.

## RESULTS

### Identification of the eIF3 subunits from *Leishmania* using bioinformatics tools

The subunit composition of the eIF3 complex seems to vary among different organisms. Since *Leishmania* are early eukaryotes, *in silico* identification of functional homologs in this organism is a major challenge. We conducted a bioinformatics survey of the trypanosomatid genome database using search engines such as BLAST and PSI BLAST to identify putative homologs of other eIF3 subunits that were originally not identified. Any hits were further verified by reverse BLAST and HHpred. In addition to subunits b, c, d, e, i and l, we identified subunits a, f, g, h, j and k. The only subunit that was not identified was subunit m. Although a candidate ORF (LmjF25.0390) was identified by a BLAST search using the human IF3m subunit as query, the reciprocal BLAST analysis revealed that this protein had a higher similarity with the csn-7 subunit of the COP9 signalosome. While this manuscript was under revision, a bioinformatics analysis of eIF3 subunits from pathogenic excavates that include trypanosomatids was published (39). In agreement with our results, these authors also reported the presence of 12 eIF3 subunits (LeishIF3 a-LeishIF3l) in trypanosomatids, however, no recognizable eIF3m was identified in this study either.

Most of the identified *Leishmania* eIF3 subunits show relatively low sequence conservation, and are shorter than their mammalian counterparts (Supplemental Figures S1–S12). For example, LeishIF3a lacks the region that parallels the human C-terminus, between positions 791–1389 (Supplemental Figure S1A). Overall, the predicted *Leishmania* a, b, c, f, h and j subunits are poorly conserved, and all identities range between 16–30%, as compared to the human orthologs. Subunits d, e and i are better conserved (Table 1). A parallel comparison of eIF3 subunits from representative members of lower eukaryotes taxa, such as *Toxoplaxma gondii, Neurospora crassa* and the diatom *Phaeodactylum tricornutum*, also revealed very low similarities to the LeishIF3 subunits (Supplemental Figures S1–S12, B,C sections). A phylogenetic analysis also highlights that the subunits of the *Leishmania* eIF3 complex branched relatively early in evolution (Supplemental Figures S1–S12, D sections).

**Table 1.** Subunit composition of the *Leishmania* eIF3 complex

Protein	Accession number	Area^b^	Relative PAF Values^a,b^ (mean value^SD^)	Identity to human protein (%)	Molecular weight (kDa)	Conserved domains
LeishIF3a	LmjF.17.0010	3.381E9	0.65^±0.13^	18.89	87.6	PCI
LeishIF3b	LmjF.17.1290	3.381E9	0.59^±0.10^	18.89	80.7	RRM
LeishIF3c	LmjF.36.6980	5.066E9	0.47^±0.08^	19.8	82	PCI
LeishIF3d	LmjF.30.3040	2.457E9	0.42^±0.06^	26.81	60.6	ND
LeishIF3e*	LmjF.28.2310	1.518E10	1.00	27.41	46.3	PCI
LeishIF3f	LmjF.25.1610	1.964E9	0.68^±0.02^	18.46	36.7	MPN
LeishIF3g	LmjF.34.2700	1.619E9	0.48^±0.2^	21.58	28.8	RRM
LeishIF3h	LmjF.07.0640	2.228E9	0.78^±0.07^	16.49	37.9	MPN
LeishIF3i	LmjF.36.3880	1.838E9	0.34^±0.01^	30.03	45.4	WD
LeishIF3j	LmjF.25.2120	1.084E8	0.13^±0.19^	18.54	23.5	ND
LeishIF3k	LmjF.32.2180	1.052E9	0.67^±0.04^	25.39	26.3	PCI
LeishIF3l	LmjF.36.0250	2.313E9	0.58^±0.08^	24.24	62.7	PCI

*Leishmania* eIF3 subunits were identified by a bioinformatics survey combined with affinity purification of the complex and mass spectrometry analysis. The LeishIF3e subunit was tagged and the purified complex were analyzed by LC-MS/MS. Relative abundance is highlighted by the peptide count, the peak area, which is the average of the three most intense peptides from each protein, and the Protein Abundance Factor (PAF). The PAF is calculated by dividing the number of unique peptides that identify the protein by the molecular weight of the protein x10^4^(40). The relative stoichiometry of subunits was obtained by normalizing the PAF value obtained for each subunit with that of the bait protein. ND - not detected.

*Bait protein.

^a^The relative PAF value is the PAF of each subunit normalized to the PAF of the bait protein.

^b^Mean of values obtained from four independent experiments.

^SD^Standard Deviation

The subunits of eIF3 contain several well-defined domains, which are known to be significant for the structural integrity of eIF3, as well as for its interactions with other translation factors. Using a variety of domain search tools, we identified the PCI domain in subunits a, c, e, k and l, while MPN domains were found in subunits f and h. As expected, an RRM domain was mapped in subunits b and g, as was a WD domain in subunit i. The expected PCI domain in LeishIF3l was not identified by any of the programs employed. However, HHpred analysis predicted a region that is similar to the TRP-like repeats that is occasionally observed in the N-terminal portion of PCI domains, including that of eIF3l homologs.

### Subunit composition of the *Leishmania* eIF3 complex - *in vivo* analysis

To verify our bioinformatics results and to identify other interactors of the *Leishmania* eIF3 complex, we performed a series of *in vivo* pull-down experiments in which the relatively conserved eIF3e subunit was SBP-tagged at the C-terminus, using the pX-H-SBP-H vector cassette (35). The SBP-tagged LeishIF3 complex was affinity purified from extracts of the transgenic parasites over streptavidin-Sepharose columns in four independent experiments. The eluted proteins were separated over SDS-polyacrylamide gels followed by LC-MS/MS analysis. A control experiment was performed with SBP-tagged luciferase following the same protocol. Relative semi-quantitative evaluation of LeishIF3 subunit abundance was based on peptide peak areas and PAF values, corresponding to the non-redundant peptide count normalized to the molecular weight of the protein(40). The mean PAF values of proteins that were present in the control pull-down experiment were subtracted from values obtained in the eIF3e purification experiments. Finally, the PAF values obtained for each protein in each independent experiment were normalized to the PAF value of the bait protein in that experiment. Tables 1, 2 and Supplemental Table S2 present the mean values of the relative PAFs obtained in four independent experiments.

**Figure 1.**
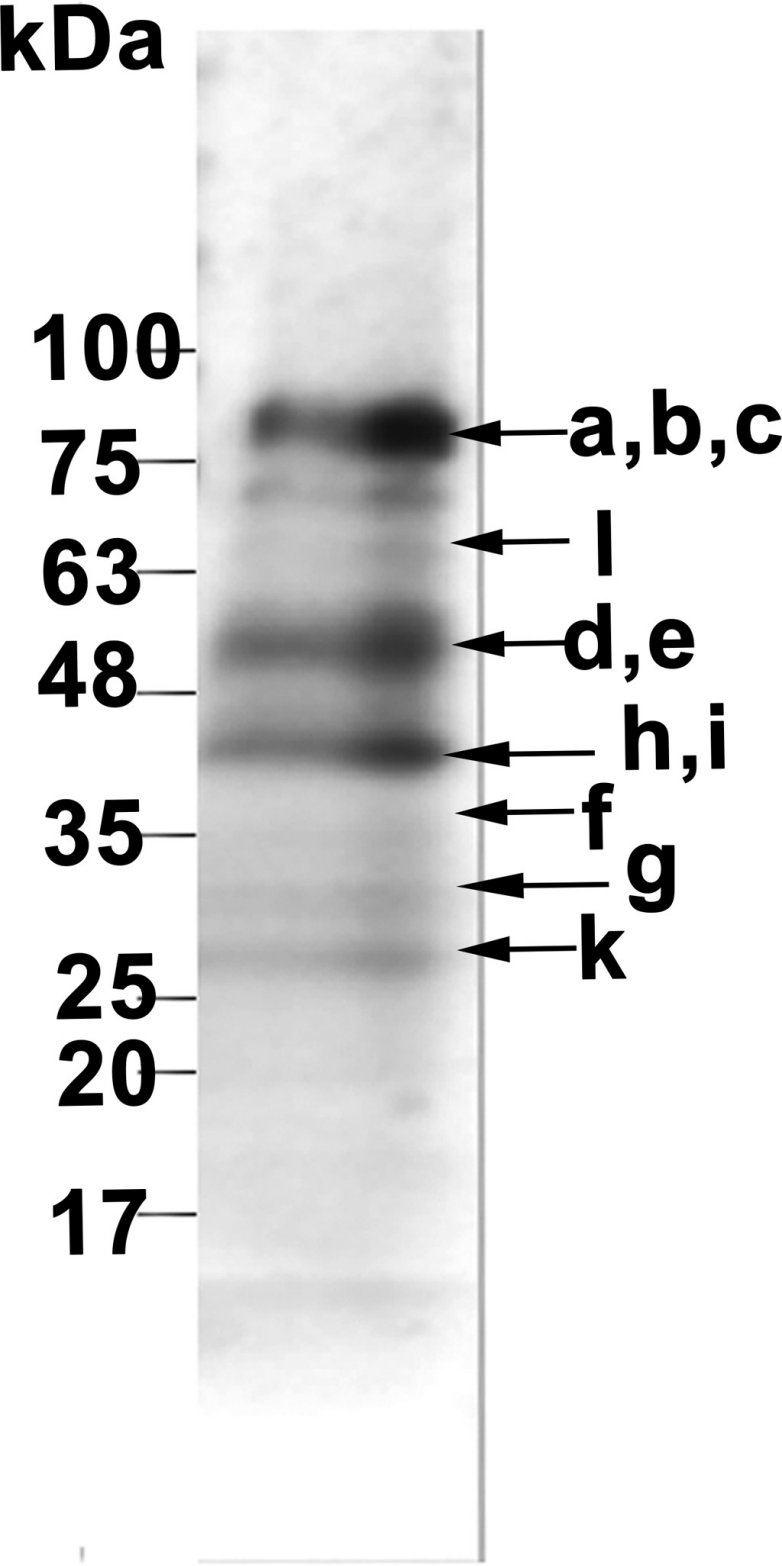
SDS-PAGE profile of the LeishIF3 complex. The LeishIF3 complex was affinity purified via the tagged LeishIF3e subunit. The interacting proteins were separated over SDS-PAGE. Each band was individually excised from the gel and subjected to LC-MS/MS analysis. The bands marked correspond to the *Leishmania* counterparts of eIF3 subunits a, b and c (LmjF17.0010, LmjF17.1290 and LmjF36.6980), l (LmjF36.0250), d and e (LmjF30.3040 and LmjF28.2310), h and i (LmjF07.0640 and LmjF36.3880), f (LmjF25.1610), g and k (LmjF34.2700 and LmjF32.2180), as identified by LC-MS/MS.

Tagging of LeishIF3e led to the consistent purification of a stable LeishIF3 complex that included 11 subunits, namely subunits a, b, c, d, e, f, g, h, i, k, but that was devoid of subunit ‘m’. The ‘j’ subunit was observed at relatively low abundance (Table 1) in some of the pull-down analyses. Nevertheless, subunit ‘m’ was not identified by bioinformatics tools or in pull-down assays. The identified subunits were also visible by SDS-polyacrylamide gel electrophoresis (SDS-PAGE), using 4–20% gradient gels. The Coomassie-stained bands were individually subjected to LC-MS/MS analysis, enabling their identification on the stained gel (Figure 1).

In the eIF3e pull-down complex, we consistently observed a high PAF value for subunit ‘h’ and a slightly lower but comparable value for subunit ‘f’. This could indicate a direct interaction between subunits ‘e’ and ‘h’ but could also be related to the reported structure of the human eIF3 complex, where subunit ‘h’ is found in the proximity of subunit ‘e’ (41). Further, we consistently observed comparable stoichiometric values for all the other PCI/MPN subunits. Although the PAF values for subunit ‘c’ were relatively low, this subunit presented the highest peak intensity among the eIF3 subunits, pointing to its strong association with LeishIF3e. The ‘b’ subunit also appeared with high PAF values and intense peaks in our pull-down assays. Subunits ‘i’ and ‘g’, which are expected to form a sub-complex with subunit ‘b’, are more peripheral(24,^41^) and were present at a lower stoichiometry (Table 1).

**Figure 2.**
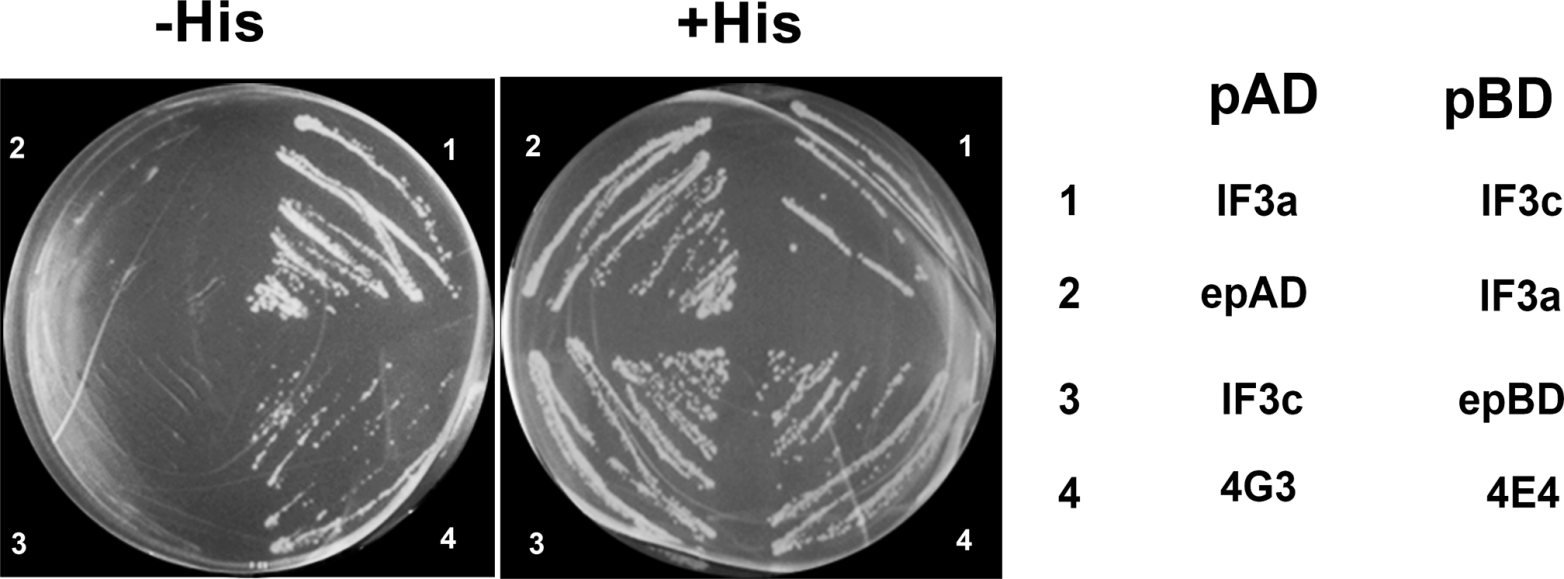
The interaction between LeishIF3a and LeishIF3c. A yeast two-hybrid assay demonstrates an interaction between LeishIF3a and LeishIF3c. The yeast strain YRG-2 was co-transfected with the yeast plasmids encoding LeishIF3a and LeishIF3c subunits fused to the AD and BD domains of GAL4, respectively, termed pAD-LeishIF3a and pBD-LeishIF3c, respectively (1). Controls that exclude false positive leaky growth were obtained by transfections with plasmid pBD-LeishIF3a and an empty pAD plasmid (2), as well as plasmid pAD-LeishIF3c and the empty pBD plasmid (3). A positive control interaction is shown between LeishIF4E4 and LeishIF4G3 (4).

Next, we tried to identify binary interactions within the *Leishmania* eIF3 complex by yeast two- hybrid assays. Among the different interactions that we tested, we identified a direct interaction between the two largest subunits of *Leishmania* IF3, subunits a and c (Figure 2). A dimer consisting of these two subunits was found to be fundamental in assembly of the human eIF3, as well as its interaction with other factors within the 43 PIC (19,20,41). Dimerization of subunits a and c is known to be promoted via their PCI domains (19). However, efforts to monitor direct interactions between other subunits by yeast two-hybrid assays failed to establish many of the conserved interactions in eIF3 (data not shown).

### eIF3-interacting proteins in *Leishmania*

Next, we tried to identify the other proteins that consistently co-purified with the eIF3 complex. A strong association was observed between the LeishIF3 complex and LeishIF1 that scored a relative high PAF value, similar to that of some of the eIF3 subunits. eIF1A was also efficiently pulled down (Table 2). Direct interaction of eIF1 or eIF1A with eIF3 in both human and yeast was previously reported (6,42,43,44). The eIF3, eIF1 and eIF1A complexes can bind to the 40S ribosome subunit, promoting the binding of TC to the 40S subunit, which results in the formation of the 43S pre-initiation complex (45,46).

**Table 2.** Translation factors associated with the eIF3 complex

	Protein	Accession Number	Area^b^	Relative PAF Values^a,b^	Molecular weight (kDa)
Initiation factors	LeishIF1 (Sui1)	LmjF24.1210	8.672E8	0.51^±0.15^	12.3
	LeishIF2α	LmjF03.0980	1.394E8	0.25 ^±0.18^	46.6
	LeishIF2β	LmjF.08.0550	5.194E7	0.17^±0.13^	38
	LeishIF2γ	LmjF.09.1070	8.968E7	0.11^±0.06^	52.5
	LeishIF5	LmjF.34.0350	2.259E7	0.19^±0.14^	42.9
	LeishIF1A	LmjF.16.0140	7.615E7	0.34^±0.05^	18.6
Elongation factors	EF1B	LmjF.34.0820	2.243E8	0.32^±0.18^	25.6
	EF2	LmjF.36.0180	2.035E7	0.25^±0.24^	94.1
	EF1γ	LmjF.09.0970	1.700E7	0.12^±0.08^	46.2
	EF1α	LmjF.17.0084	4.971E7	0.15^±0.2^	49.1

LC-MS/MS analysis of the eIF3e pull-down complex shows consistent co-purification of eIF1, eIF2, eIF5 and eIF1A and the eIF3 complex, with a high relative protein abundance factor. The eIF3 interactome also contains elongation factors.

^a^The relative PAF value represents the PAF of each subunit normalized to the PAF of the bait protein.

^b^Mean of values obtained from four independent experiments.

Among the other initiation factors that co-purified with tagged LeishIF3e, relatively high PAF values for eIF2 and eIF5 were consistently observed. This could indicate the occurrence of a strong association between eIF3 and these factors along with eIF1, supporting the presence of a putative multi-factor complex (MFC) that usually forms independently of the ribosome, as observed in human, plant and yeast cells (43,47,48). The MFC is required for initiation complex assembly and ribosome scanning, as well as for AUG recognition *in vivo* (6,43,49). The strong association of LeishIF3 with other MFC components was also evident from our pull-down assays, which captured another tagged subunit, LeishIF3a (Supplemental Table S3). The presence of 40S ribosomal proteins is expected, as unassembled ribosomal 40S subunits are stably associated with eIF3 in the cytoplasm (50) and remain associated throughout the initiation phase of translation. The mRNA-associated proteins LeishIF4G3, LeishIF4E4 and LeishPABP1 appeared only in some of our purification samples with much lower PAF values. The low abundance of eIF4F complex in our purification could be due to the transient or weak nature of its interaction with the 43S PIC.

**Figure 3.**
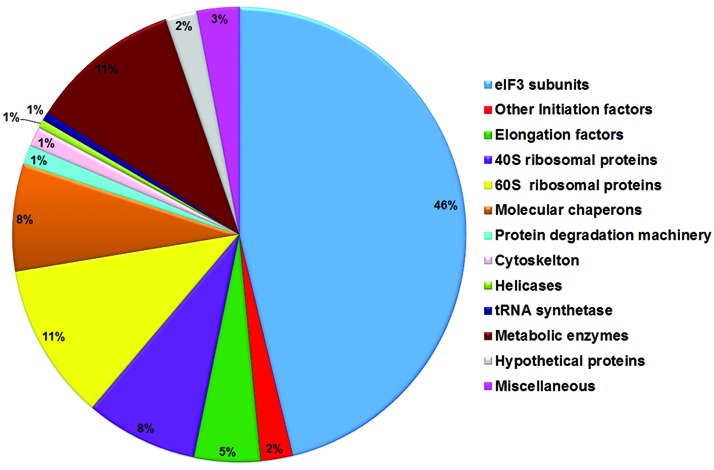
A pie chart showing the relative abundance of the proteins identified by LC-MS/MS in the pulled down LeishIF3e complex. The identified proteins that were pulled down with LeishIF3e were assigned to different functional categories. Relative quantification of each group was based on the sum of the peptide ion peak area of individual proteins in the group. The values obtained for each group was then normalized against the total peak areas in the individual run and are given in percentage. The representative values are the mean of three independent experiments.

Results of the pull-down experiment revealed the presence of additional translation factors, like eIF4A, eIF6, eIF5A2, and elongation factors, as well as 60S ribosomal proteins (Supplemental Table S2). The relatively high abundance of 60S ribosomal proteins and elongation factors appears to be in conflict with the fact that the majority of the roles assigned to eIF3 deal with the initiation phase of translation. Overall, our observation is in agreement with an earlier report that characterized the highly specific interactome of fission yeast eIF3, showing the presence of 60S subunits and translation elongation factors (51). Apart from these factors, the eIF3 interactome also contains molecular chaperones, tRNA synthetases, RNA helicases and many proteins associated with the cytoskeleton. Molecular chaperones related to Hsp70 and Hsp83, chaperonin-containing t-complex proteins (CCT) and stress inducible protein (Sti1) homologs were relatively abundant in our purifications. Some of these proteins are known to be involved in the co-translational folding of newly synthesized polypeptides (52). A significant enrichment of microtubules and associated proteins was noticed in the LeishIF3 purification, in accordance with earlier reports on the association of eIF3 with tubulin and other cytoskeleton components (53). Proteins involved in the ubiquitination system were also co-purified with the eIF3 complex with relatively high abundance, in line with reports on the translasome mega-complex (51). Finally, metabolic enzymes account for a significant fraction (11%) of the eIF3-associated complex. Owing to their high abundance within the cells, these proteins could be purified as nascent polypeptide along with actively translating ribosomes (51). It is possible that many of the interactions described above, could occur indirectly through their association with ribosomes that were co-purified in our pull-down experiments. The presence of metabolic enzymes could also represent moonlighting events that were recently reported (54). Indeed, some of these enzymes were previously shown to also contain RNA-binding domains (55). The eIF3-interacting proteins identified in the LC-MS/MS analyses were functionally clustered and a pie chart based on peptide ion peak area was drawn, showing the relative abundance of each group. As expected, the eIF3 subunits make up the most prominent fraction (46%) in the purified sample (Figure 3).

### Recruitment of LeishIF3 to the translation initiation complex in *Leishmania* promastigotes

One of the most significant interactions in eukaryotic translation initiation occurs between eIF3 and eIF4G. The latter mediates eIF3 recruitment to the initiation complex. However, this interaction is not conserved in yeast, where translation initiation complexes are recruited to mRNA in a manner stimulated by eIF3 and eIF2, yet independent of eIF4G (56). This prompted us to address how eIF3 is recruited to the 48S pre-initiation complex in *Leishmania*. To investigate this point, reciprocal pull-down experiments were carried out with tagged transgenic LeishIF4G3 and LeishIF4E4, expressed in *L. amazonensis* promastigotes. Targeting tagged LeishIF4G3 resulted in efficient capture of LeishIF3 subunits, as evident from significantly high PAF values obtained for most of the subunits. At the same time, the ability of tagged LeishIF4E4 to bring down LeishIF3 subunits was much weaker (Table 3). A complete list of LeishIF4G3-interacting proteins identified by LC-MS/MS analysis is given in Supplemental Table S4. LeishIF4E4-interacting proteins have been previously described (35).

**Table 3.** LeishIF4G3 recruits the eIF3 complex

		Relative PAF		
Protein	Accession number	LeishIF4G3 Pull-down Complex	LeishIF4E4 Pull-down Complex	Molecular Weight (kDa)
LeishIF4G3	LmjF16.1600	1.00	0.92	71.2
LeishIF4E4	LmjF30.0450	0.57	1	33.8
LeishIF4A	LmjF.01.0770	0.47	0.35	45.3
LeishIF3a	LmjF.17.0010	0.23	-	87.6
LeishIF3b	LmjF.17.1290	0.26	-	80.7
LeishIF3c	LmjF.36.6980	0.28	0.03	82
LeishIF3d	LmjF.30.3040	0.09	-	60.6
LeishIF3e	LmjF.28.2310	0.25	-	46.3
LeishIF3f	LmjF.25.1610	0.09	0.05	36.7
LeishIF3g	LmjF.34.2700	0.11	-	28.8
LeishIF3h	LmjF.07.0640	0.18	0.12	37.9
LeishIF3i	LmjF.36.3880	0.10	0.02	45.4
LeishIF3k	LmjF.32.2180	0.13	0.03	26.3
LeishIF3l	LmjF.36.0250	0.14	-	62.7

Reciprocal pull-down assays were carried out using SBP-tagged LeishIF4G3 and LeisheIF4E4. LC-MS/MS analysis shows that LeishIF4G3 captures eIF3 subunits more efficiently than LeishIF4E4, suggesting that LeishIF4G3 plays a central role in the recruitment of LeishIF3.

**Figure 4.**
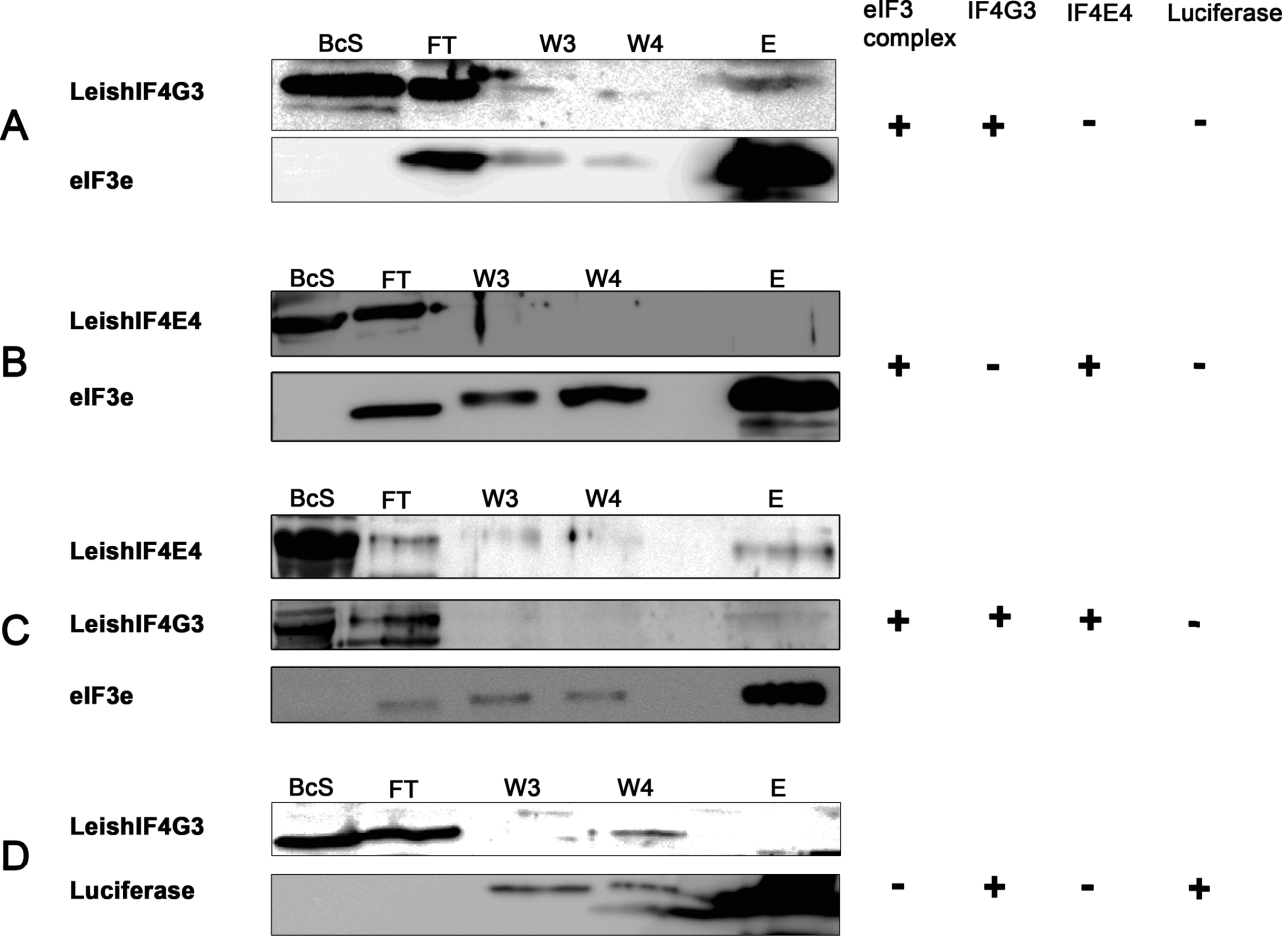
eIF4G3 interacts with the eIF3 complex in *Leishmania* promastigotes. Extracts of cells expressing tagged LeishIF3e were incubated with streptavidin-Sepharose beads, washed with PRS and further incubated with purified recombinant LeishIF4G-3, LeishIF4E-4, or with both recombinant proteins. The mixture was washed again and eluted upon addition of biotin (5 mM) in PRS+. Samples were taken from the total bacterial extracts (BcS; 20 μl containing ∼0.2–0.8 μg of the recombinant protein). The other fractions, including flow-through (FT, 100%), washes (W, 100%) and the elution (E, 100%) were TCA-precipitated. These were separated by SDS-PAGE and subjected to western analysis using antibodies against LeishIF4G-3 or LeishIF4E-4, along with antibodies against the SBP tag that identified the tagged LeishIF3e bait. Panel **A** shows the interaction between LeishIF3 and recombinant LeishIF4G-3, which co-purified with the LeishIF3 complex. Panel **B** excludes the occurrence of an interaction between the LeishIF3 complex and recombinant LeishIF4E4. Panel **C** shows that Leish4E4 co-purified with the LeishIF3 complex only in the presence of LeishIF4G3, suggesting that the interaction between LeishIF3 and Leish4E4 is mediated by LeishIF4G3. Panel **D** represents a negative control with SBP-tagged luciferase and recombinant LeishIF4G3.

These results highlight the potential role of LeishIF4G3 in recruitment of the LeishIF3 complex and the 40S ribosomal subunit. An *in vitro* approach was employed to further establish that LeishIF4G3 directly interacts with the LeishIF3 complex. Bacterial extracts containing recombinant LeishIF4G3 were incubated with SBP-tagged LeishIF3 complexes, bound to streptavidin-Sepharose beads. After additional washes, the bound complex was eluted by biotin. The eluted material was subjected to western analysis using antibodies against LeishIF4G3 and against the SBP tag (the latter serving to identify the LeishIF3e subunit). Figure 4A shows that recombinant LeishIF4G3 co-eluted with the LeishIF3 complex, indicating that LeishIF4G3 binds directly to the LeishIF3 complex. The absence of any endogenous eIF4E4 or eIF4A1 in the complex mediating the interaction was verified by re-probing the blots with antibodies against these proteins. The direct nature of the interaction between LeishIF4G3 and LeishIF3 was further emphasized by the inability of the latter to interact with recombinant LeishIF4E4 in a parallel assay (Figure 4B). However, if recombinant LeishIF4G3 was also included in the binding mixture, along with the recombinant LeishIF4E4, the latter could be pulled down by the tagged IF3 complex (Figure 4C), most probably through its interaction with LeishIF4G3. A control co-purification experiment was carried out between recombinant LeishIFG3 and the unrelated SBP-tagged luciferase. As expected, the recombinant LeishIF4G3 did not co-elute with the tagged luciferase, further confirming the efficient binding between LeisIF4G3 and LeishIF3 (Figure 4D).

### The cap-binding protein LeishIF4E1 interacts with the LeishIF3 complex

**Figure 5.**
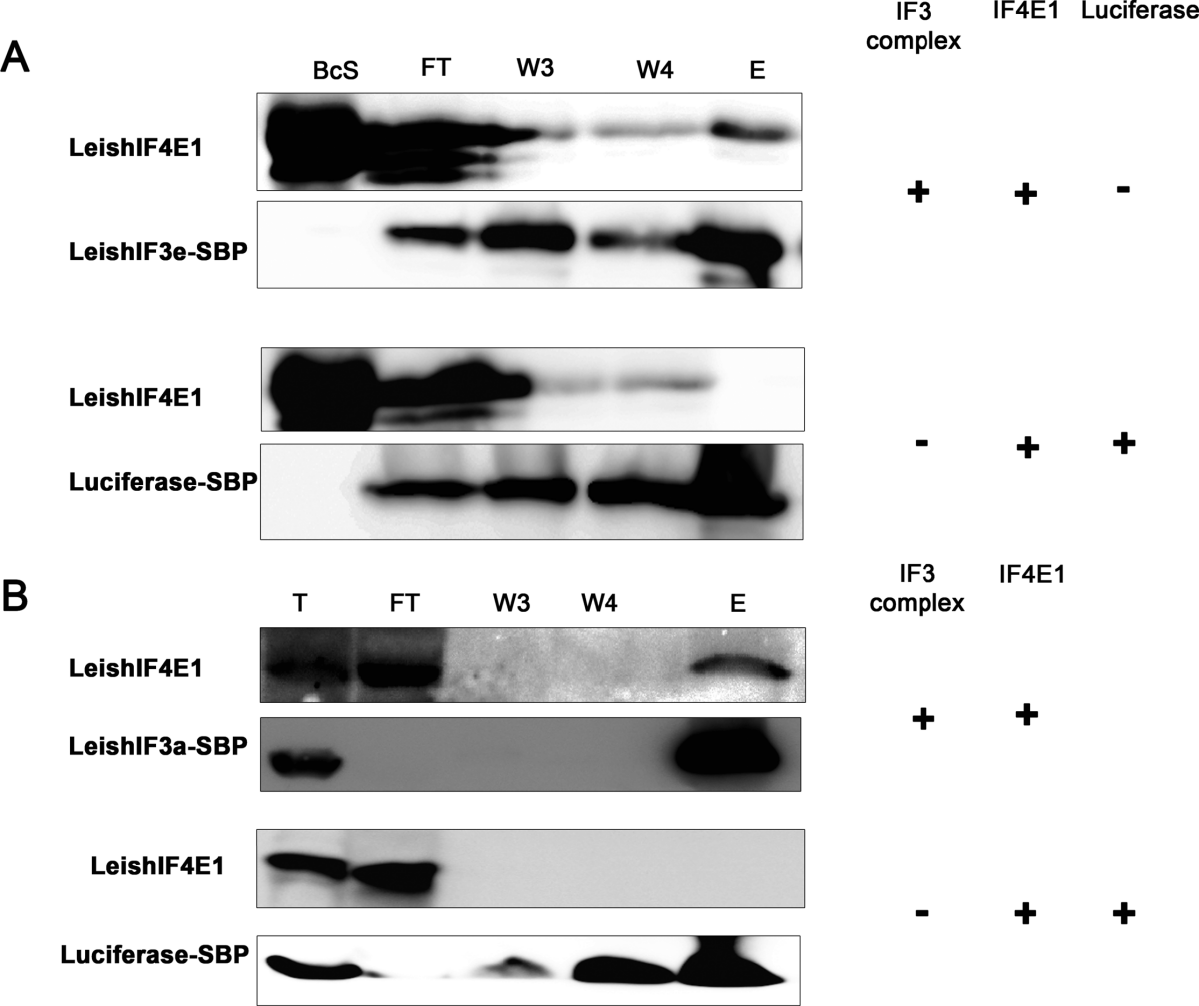
LeishIF4E1 directly interact with LeishIF3 in an eIF4G-independent manner. (**A**) Recombinant LeishIF4E1 was allowed to interact with the LeishIF3 complex immobilized on streptavidin Sepahrose beads as described in the legend to Figure 4 and interaction was confirmed by detecting LeishIF4E1 in the elution using specific antibodies raised against LeishIF4E-1 and the SBP tag. A similar experiment was run with luciferase and LeishIF4E1 to assess the extent of non-specific interactions. (**B**) An *in vivo* pull-down experiment showing the LeishIF4E1 was co-purified with LeishIF3 complex when overexpressed. The cell lysate of an untagged LeishIF4E1-expressing cell line was mixed with lysate from a SBP-tagged LeishIF3a cell line. The LeishIF3a-associated complex was then purified over streptavidin-Sepharose beads and the eluate was checked for the presence of LeishIF4E1. A control experiment was conducted by mixing an untagged LeishIF4E1-expressing cell line with a SBP-tagged luciferase-expressing cell line.

We previously reported on changes that occur in the endogenous *Leishmania* cap-binding complex following exposure of the parasites to elevated temperatures. It was shown that at the mammalian-specific temperature, the canonical LeishIF4E4 complex is inactivated, as LeishIF4E4 no longer binds to the cap structure, whereas LeishIF4E1 maintains its cap-binding activity. Since LeishIF4E1 does not interact with any eIF4G ortholog, including LeishIFG3, (35) it was unclear how LeishIF3 was recruited to the LeishIF4E1–mediated complex. To examine whether a direct interaction could be detected between LeishIF3 and LeishIF4E1, an experimental approach was employed, similar to that described for LeishIF4G3. Accordingly, purified LeishIF3e complex bound to streptavidin-Sepharose beads was allowed to interact with recombinant LeishIF4E1, The eluted material was subjected to western analysis using antibodies against LeishIF4E1 and against the SBP tag (the latter serving to identify the LeishIF3e subunit). The presence of LeishIF4E1 in the pull-down extracts is demonstrated in Figure 5A. A control experiment conducted with SBP-tagged luciferase-expressing cells (as described in Figure 4) excluded non-specific binding of LeishIF4E1 to the beads.

Although tagged endogenous LeishIF4E1 efficiently pulled down the LeishIF3 complex, as previously shown (35), it was difficult to monitor the components of the cap-binding complexes in the LeishIF3e pull-down extracts. This could indicate that the majority of LeishIF3 was associated with free 40S ribosomal subunits, or with the MFC complex. The low level of cap-binding proteins in the cells could also contribute to this difficulty. However, when LeishIF4E1 was abundant in the pre-pull-down lysate, its interaction with the LeishIF3 complex was evident. This was revealed by mixing cells expressing tagged LeishIF3a with cells that expressed non-tagged LeishIF4E1. The mixed cells were disrupted and subjected to affinity purification over Streptavidin beads. Following elution of the tagged LeishIF3 complex with biotin, the presence of the untagged LeishIF4E1 in the eluted mixture was demonstrated on western blots. A control experiment in which tagged LeishIF3a cells were exchanged with tagged luciferase failed to show the presence of LeishIF4E1 in the complex that was pulled down by the tagged luciferase (Figure 5B).

### The cap-binding protein LeishIF4E1 interacts with C-terminus of LeishIF3a

**Figure 6.**
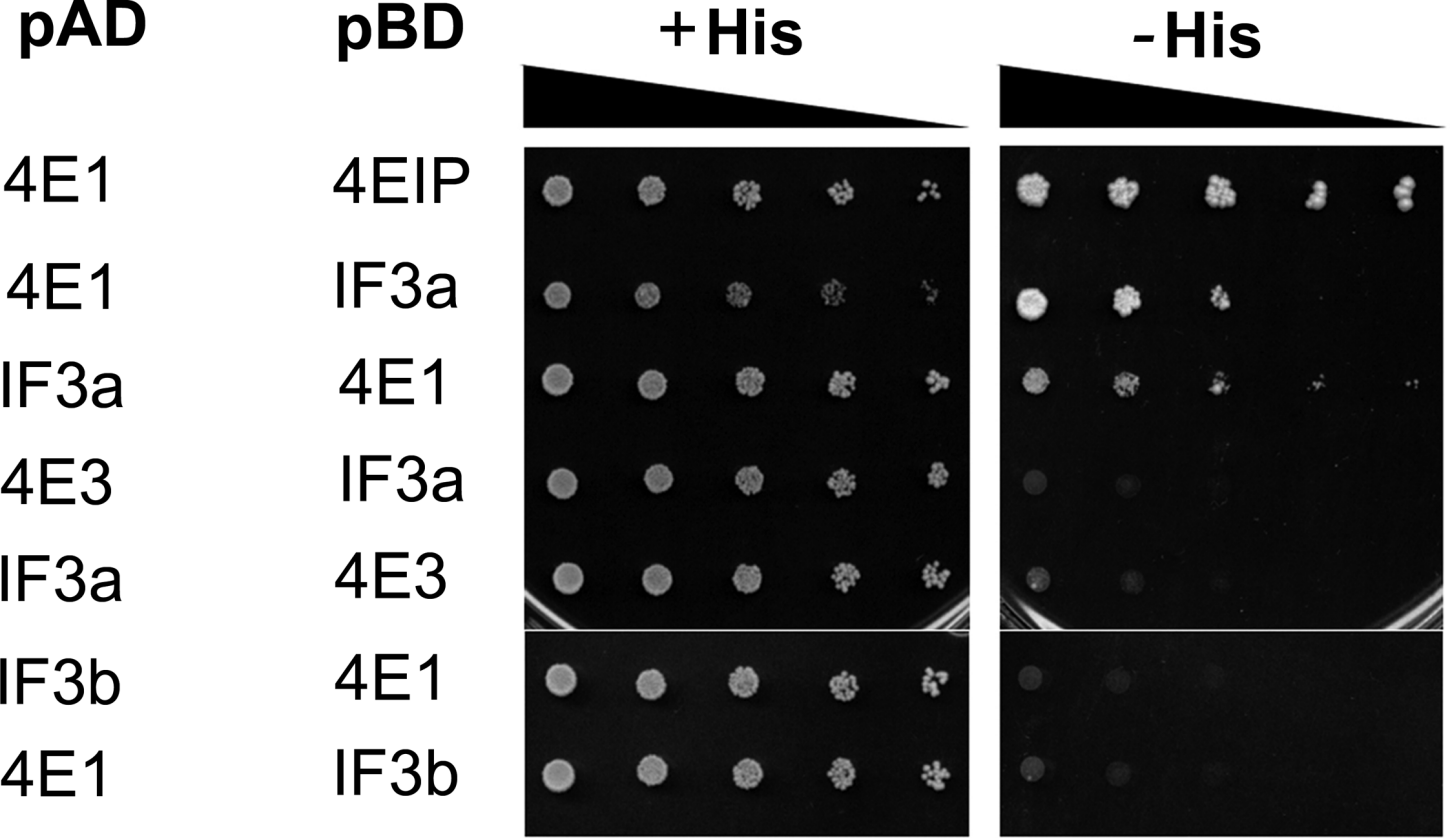
4E1 interacts with the ‘a’ subunit of eIF3. Wild type YRG2 cells were co-transfected with yeast plasmids encoding the AD and BD fusion proteins. The BD and AD sequences were fused to the open reading frames of LeishIF4E1 or LeishIF3a. The cells were cultured under permissive (+His) and restrictive (-His) conditions and spotted in three-fold dilution steps. The black triangle represents decreasing concentrations generated by X3 fold dilutions of the spotted yeast cells, up to 1:81. The interaction between LeishIF4E1 and Leish4EIP served as a strong positive control. Negative controls are represented by the interaction between LeishIF3a and LeishIF4E3, cloned reciprocally in the pBD and pAD plasmids. Another negative control is represented by the lack of interaction between LeishIF3b and LeishIF4E1, reciprocally cloned in plasmids pBD and pAD.

The interaction between LeishIF4E1 and LeishIF3 was further verified by yeast two-hybrid experiments, using LeishIF4E1 and subunits of the LeishIF3 complex. A direct interaction was observed between LeishIF4E1 and LeishIF3a, as indicated in Figure 6. The interaction was confirmed by reciprocal yeast two-hybrid assays, along with positive and negative controls, in which LeishIF4E1 was replaced by LeishIF4E3. The positive control consisted of LeishIF4E1 and its previously reported binding partner Leish4E-IP (35). No interaction was observed between LeishIF4E1 and LeishIF3b (Figure 6), or with other LeishIF3 subunits (data not shown).

**Figure 7.**
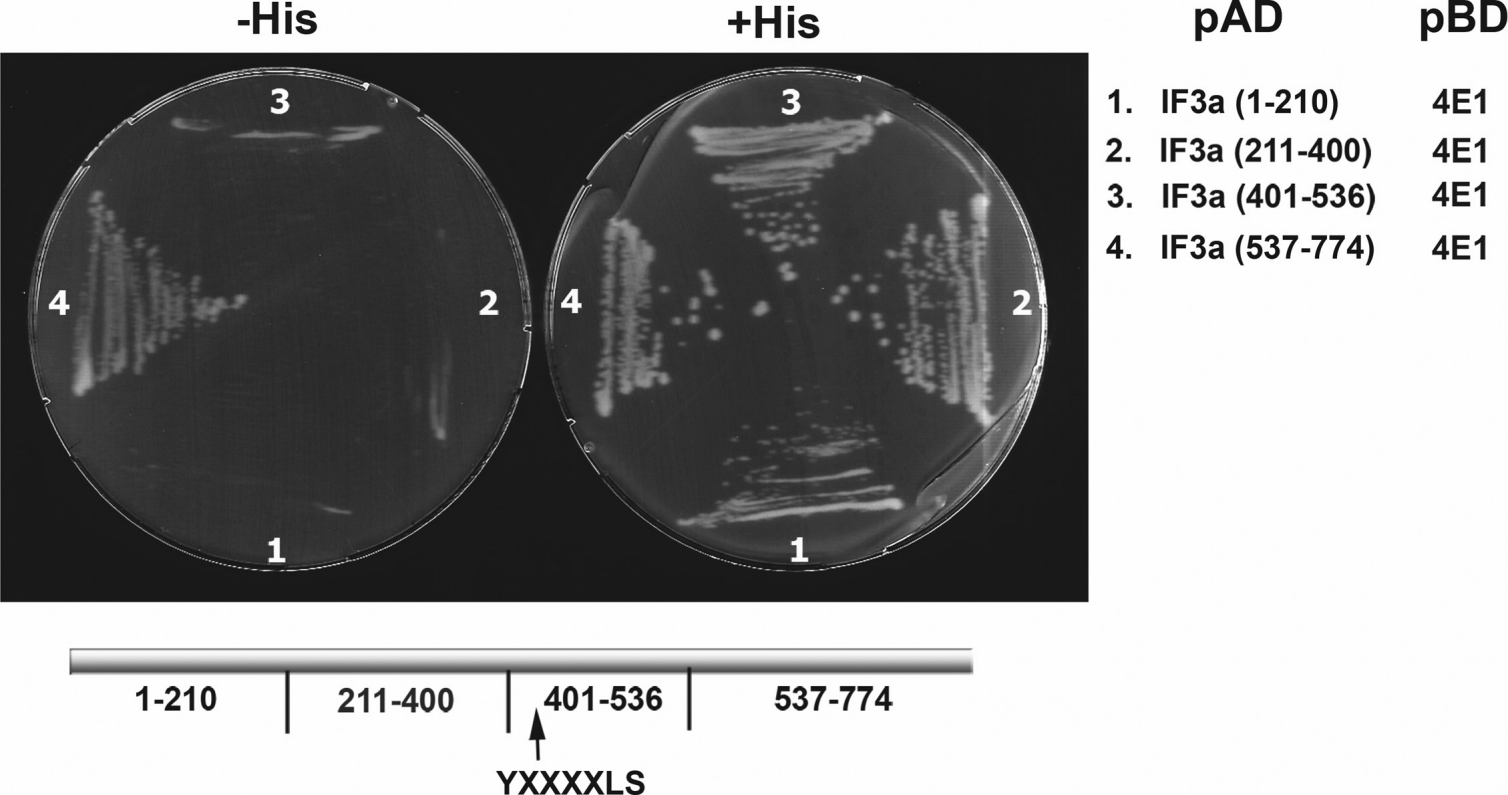
LeishIF4E-1 interacts with the C-terminal region of LeishIF3a. The interaction between LeishIF4E-1 and LeishIF3a was mapped using the yeast two-hybrid assay. LeishIF3a was fragmented into four segments corresponding to residues 1–210, 211–400, 401–536 and 537–774. DNA encoding each of these fragments was cloned into the yeast pAD vector, fused to the GAL4 transcription activation domain. LeishIF4E1 was cloned into the yeast pBD vector, fused to the GAL4 DNA-binding domain. The C-terminal region lacks a putative YXXXXLφ element, suggesting a non-canonical interaction between the two proteins.

LeishIF3a was fragmented to identify the potential eIF4E-binding region in the protein using a yeast two-hybrid assay. DNA encoding LeishIF3a fragments corresponding to positions 1–210, 211–400, 400–536 and 537–774 were generated and cloned into the yeast pAD vector and tested for their ability to interact with LeishIF4E1, cloned in the pBD vector. Figure 7 shows that the C-terminal fragment of IF3a, corresponding to residues 537–774, interacted with the cap-binding protein ortholog LeishIF4E1. Interestingly, this fragment did not include the consensus element found in most eIF4E-binding proteins, further emphasizing that this interaction could be based on an alternative structural basis. It was especially intriguing to realize that a putative conserved element (YXXXXLS) was found between positions 435–441 of IF3a. This motif bears similarity to the consensus YXXXXLφ sequence, although the serine residue could compromise the ability to interact with LeishIF4E1. Monitoring expression of the various fragments in the transfected yeast by western analysis indicated the strong expression of LeishIF3a fragments 210–400 and 537–774 but weaker expression of fragments 1–210 and 400–536 (Supplemental Figure S13). To further validate that the binding was mainly directed by the C-terminus and not by other parts of LeishIF3a, additional fragments spanning positions 1–400 and 211–536 were generated. These two fragments were designed to be larger than the earlier fragments and to overlap. Fragment 211–536 included the YXXXXLS sequence element yet failed to bind LeishIF4E1 in the yeast two-hybrid assay. Only fragment 537–774 could bind LeishIF4E1. Thus, binding was directed by the LeishIF3a C-terminal fragment (residues 537–774) (Supplemental Figure S14A). The different fragments were highly expressed in the transformed yeast cells (Supplemental Figure S14B), confirming that binding of LeishIF4E1 by LeishIF3a was mainly directed by the C-terminus of the latter.

## DISCUSSION

In this study, we characterized the eIF3 complex from *Leishmania*, a kinetoplastid known for its unusual genome organization, RNA metabolism and translation (57). Despite the large evolutionary distance between *Leishmania* and mammals, the two encode an eIF3 complex with compositional similarity. A bioinformatics search of the *Leishmania* genome revealed 12 subunits (LeishIF3a through LeishIF3l) of the expected 13 known subunits; subunit LeishIF3m was not identified by this approach. Affinity purification of the LeishIF3 complex verified the presence of all 12 LeishIF3 subunits (a-l), except for the missing ‘m’ subunit. LeishIF3j was observed at a lower stoichiometry, possibly due to its loose association with the complex, as observed in other eukaryotes (51,58,59,60).

The presence of a ‘human-like’ eIF3 complex in *Leishmania* suggests that the presence of the complex dates back to the appearance of early eukaryotes. The fact that the *S. cerevisiae* genome encodes a simpler version of the eIF3 complex, containing only six subunits, shows that during evolution, this organism selectively retained only those subunits essential for eIF3 activity. In line with this hypothesis, a recent study showed that yeast eIF3 binds to 40S in a clamp-like manner, where it can engage other initiation factors, efficiently facilitating all the core functions of eIF3 (19).

PCI complexes from higher eukaryotes, namely the proteasome lid, the COP9 signalosome and eIF3, are conserved and share a common architecture (61). However, less information is available on these complexes from lower eukaryotes. The *Leishmania* genome database was, therefore, searched for the proteasome lid and COP9 signalosome subunits, using bioinformatics tools. The proteasome lid of *Leishmania* and trypanosomes contains the full repertoire of the eight expected PCI subunits, whereas the search for COP9 subunits revealed only four in *Leishmania* (CSN2, CSN4, CSN5 and CSN7). Two additional putative subunits were identified in *Trypanosoma* species (CSN1 and CSN6) (Table 4). While the possibility that the missing subunits have diverged beyond recognition cannot be excluded, the COP9 complex could also be complemented for some of its missing subunits by introduction of the parallel subunits of LeishIF3 or the proteasome lid, as shown for *Caenorhabditis elegans* and yeast (62,63,64). Remarkably, the *Leishmania* ortholog of the COP9 signalaosome CSN7 subunit shows significant similarities to eIF3m family members. In *C. elegans*, the only CSN7 ortholog identified, CIF-1, shows a high degree of sequence homology to eIF3m and was shown to be a part of both eIF3 and COP9 signalosome complexes (63). However, our pull-down analysis did not offer any indication that supports a possible connection between LeishCSN7 and LeishIF3.

**Table 4.** PCI complex of Leishmania

eIF3		Proteasome lid		COP9 signalosome	
Human	*Leishmania major*	Human	*Leishmania major*	Human	*Leishmania major/Trypanosoma spp*.
eIF3a	LmjF17.0010	Rpn5	LmjF21.0760	Csn4	LmjF.36.6510
eIF3c	LmjF36.6980	Rpn6	LmjF02.0370	Csn2	LmjF.25.2210
eIF3e	LmjF28.2310	Rpn7	LmjF32.2820	Csn1	*T. brucei* (Tb927.10.2510) and *T. cruzi* (TcCLB.507883.60)
eIF3l	LmjF36.0250	Rpn3	LmjF27.1460	Csn3	ND
eIF3k	LmjF32.2180	Rpn12	LmjF32.1200	Csn8	ND
eIF3h	LmjF.07.0640	Rpn8	LmjF32.0390	Csn6	*T. brucei* Tb927.8.3640
eIF3f	LmjF25.1610	Rpn11	LmjF34.0650	Csn5	LmjF.16.0850
eIF3m	ND	Rpn9	LmjF19.1120	Csn7	LmjF25.0390

*L. major* subunit orthologs of the three PCI complexes: eIF3, the proteasome lid and the COP9 signalosome. The orthologs were identified by BLAST/psiBLAST searches. ND—not detected.

The recently solved structure of the *Trypanosoma brucei* ribosome highlights trypanosome-specific features, such as extraordinarily large RNA expansion segments and protein extensions. It was suggested that the ribosomal expansion segments, located in the proposed binding site for eIF3, could lead to an unusual mode of interaction between the ribosome and eIF3 in trypanosomes (65). The non-conserved nature of *Leishmania* eIF3 subunits, especially the divergence observed in those domains which are known to be crucial for ribosome binding, further make this interaction an interesting element for additional investigation.

The association of eIF1 with eIF3 is critical for binding of the latter to 40S ribosomes and strong biochemical evidence exists for the physical association between the two factors (66,67). Accordingly, we discerned a strong association of eIF1 with LeishIF3 in all our pull-down analyses, and in most of the cases eIF1 was found in equal stoichiometry with eIF3 subunits. eIF1 and eIF3 are both components of the multifactor complex (MFC), which is ribosome-free. Other components of the MFC, eIF2 and eIF5, were also co-purified at relatively high abundance. These factors, however, showed a stronger association with LeishIF3 when LeishIF3a served as the bait protein (Supplemental Table S3). Our results could be taken as a preliminary evidence for the possible existence of an MFC in *Leishmania*, although it is yet unclear whether this complex forms independently of ribosomes in this organism.

In higher eukaryotes, the eIF3 complex is recruited to the 48S PIC via the scaffold protein eIF4G. Since the canonical LeishIF4G-3 is shorter in *Leishmania* than in its mammalian counterpart, and is, therefore, missing some of the functions of the mammalian component (35), it was important to verify the existence of this interaction in *Leishmania*. Indeed, the LeishIF3 complex was clearly able to pull-down recombinant LeishIF4G3 in an *in vitro* binding assay. Furthermore, the LeishIF3 complex could directly interact with LeishIF4G3 but not with its binding partner LeishIF4E4, which served as a negative control for non-specific binding. LeishIF4E4 was found in the pulled down extracts only when LeishIF4G3 was included in the reaction, acting as a bridge between LeishIF3 and LeishIF4E4.

It was shown that LeishIF4E4 loses its ability to bind the cap-structure and LeishIFG3 after prolonged exposure to mammalian-like temperatures. Under these conditions, the only endogenous cap-binding protein that maintained its ability to bind m^7^GTP was LeishIF4E1. However, LeishIF4E1 does not complex well with any MIF4G-domain protein, making it unclear how LeishIF3 was recruited to its complex. Pull-down experiments with tagged LeishIF4E1 showed that the protein brings down a multitude of translation initiation factors, excluding only the eIF4G orthologs (35). An *in vitro* co-purification assay indeed showed direct interaction between the endogenous LeishIF3 complex and the recombinant LeishIF4E1. Furthermore, a yeast two-hybrid analysis between LeishIF4E1 and different LeishIF3 subunits highlighted that LeishIF3a could bind LeishIF4E1. This interaction is not known from higher eukaryotes, and could serve as a basis for the recruitment of LeishIF3 to the LeishIF4E1 complex. Furthermore, the interaction was mapped to the C-terminal region of LeishIF3a, a segment that does not contain the YXXXXLφ element found in most eIF4E-binding proteins of higher eukaryotes (68). This is thus the second example in *Leishmania* of a protein that interacts with an eIF4E ortholog devoid of this element, as we earlier showed that LeishIF4G-4 binds efficiently to the eIF4E paralog LeishIF4E-3 through a region that does not contain the consensus YXXXXLφ motif (34).

The role of LeishIF4E1 is still not fully resolved, although we favor the hypothesis that it acts as a translation factor that functions during prolonged stresses. Alternatively, it could serve as a cap-binding protein that protects inactive mRNAs during stress, either anchoring a protective complex on the mRNA, or serving as a dedicated cap-binding protein. In any case, LeishIF4E1 anchors a complex that contains LeishIF3, with the latter being recruited via the LeishIF3a subunit. Our study points to the versatility of cap-binding proteins in *Leishmania* as they engage in novel interactions to accommodate changes in protein expression that will ultimately assist them in overcoming the damaging effects inflicted by the continuous exposure to stress conditions.

## SUPPLEMENTARY DATA

Supplementary Data are available at NAR Online.

SUPPLEMENTARY DATA
