# Supplementary material for: The eIF3 complex of Leishmania—subunit composition and mode of recruitment to different cap-binding complexes
Source: Nucleic Acids Res. 2015 Jun 19;43(13):6222–35. doi: 10.1093/nar/gkv564 (PMC4513851; doi:10.1093/nar/gkv564)
Supplement: SUPPLEMENTARY DATA [file supp_43_13_6222__index.html]

The eIF3 complex of Leishmania—subunit composition and mode of recruitment to different cap-binding complexes — SUPPLEMENTARY DATA 

# The eIF3 complex of *Leishmania*—subunit composition and mode of recruitment to different cap-binding complexes

## SUPPLEMENTARY DATA

- SUPPLEMENTARY DATA
- SUPPLEMENTARY DATA
- SUPPLEMENTARY DATA
- SUPPLEMENTARY DATA
- SUPPLEMENTARY DATA
- SUPPLEMENTARY DATA
- SUPPLEMENTARY DATA
- SUPPLEMENTARY DATA
- SUPPLEMENTARY DATA
