# Supplementary material for: The eIF3 complex of Leishmania—subunit composition and mode of recruitment to different cap-binding complexes
Source: Nucleic Acids Res. 2015 Jun 19;43(13):6222–35. doi: 10.1093/nar/gkv564 (PMC4513851; doi:10.1093/nar/gkv564)
Supplement: SUPPLEMENTARY DATA [file supp_gkv564_nar-01173-v-2015-File009.docx]

| **No** | **Primer** | **Gene/position** | **Sequence** | **Restriction sites** |
| --- | --- | --- | --- | --- |
| 1 | LeishIF3e-Fwd | LmjF28.2310/1-22 | GCAGGATCC ATGGACATGCTAACGAAGCTGT | BamHI |
| 2 | LeishIF3e-Rev no stop codon | LmjF28.2310/1196-1218 | CTGTCTAGA ACGCATAACGGTGTCTAGCTTG | Xba1 |
| 3 | LeishIF4G3-Fwd | LmjF16.1600/1-20 | CGGGATCC ATGCAGTTCA CCGTGGAGCA | BamH1 |
| 4 | LeishIF4G3-Rev no stop codon | LmjF16.1600/1890-1908 | ATATCTAGACTTGGGGAAGCGCTCCTT | Xba1 |
| 5 | LeishIF3a-Fwd | LmjF17.0010/1-20 | GCAGGATCC ATGGATCGCGCAGCAACCTC | BamH1 |
| 6 | LeishIF3a-Rev | LmjF17.0010/ 2304-2325 | CTGTCTAGA TCATTCGCCCCCGAGACGG | Xba1 |
| 7 | LeishIF3a-Fwd | LmjF17.0010/1-20 | ATCGT GAATTC ATGGATCGCGCAGCAACCTC | EcoR1 |
| 8 | LeishIF3a Rev | LmjF17.0010/611-630 | ATCGT GAATTC TCA GTCCAGCGCCATGTGCGATA | EcoR1 |
| 9 | LeishIF3a-Fwd | LmjF17.0010/631-650 | ATATAT GAATTC CACCTGCACGACCACGACG | EcoR1 |
| 10 | LeishIF3a  Rev | LmjF17.0010/1180-1200 | ATCGT GAATTC TCA GTTGGGGTCCTCGAGGTTGG | EcoR1 |
| 11 | LeishIF3a  fwd | LmjF17.0010/1201-1221 | ATCGT GAATTC TTCTCCGACTTCACCGGATTG | EcoR1 |
| 12 | LeishIF3a  Rev | LmjF17.0010/1588-1608 | ATCGT GAATTC TCA CGATGAAGACGCGGCAACGT | EcoR1 |
| 13 | LeishIF3a  fwd | LmjF17.0010/1609-1629 | ATCGT GAATTC AAGAGGCAGGTGACTCCGTC | EcoR1 |
| 14 | LeishIF3a  Rev | LmjF17.0010 2304-2325 | ATCGT GAATTC TCATTCGCCCCCGAGACGG | EcoR1 |
| 15 | LeishIF3c  Fwd | LmjF36.6980  1-26 | CGC GAATCC ATG AAC TTT TTC GCG ATC AGC TCT AG | EcoR1 |
| 16 | LeishIF3c  Rev | LmjF36.6980  2176-2196 | GCTGTCGACTTA GCG GCT GCC ACG GCC AC | Sal1 |
| 17 | LeishIF4E-1 Fwd | LmjF27.1620/1-21 | CGCGGATCCATGTCAGCCCCGTCTTCAGTT | BamHI |
| 18 | Leish4E-1 Rev | LeishIF4E-1/623-642 | GCATCTAGATTAGACTAAGACGCCTCGCCGTGC | Xba1 |
| 19 | Leish4E-4 Fwd | Leish4E-4/1-20 | CGCGGATCCATGAACCCCAACGCCACGGA | BamH1 |
| 20 | Leish4E-4  Rev no stop codon | LmjF30.0450/903-924 | GCATCTAGAGTAGCGCCGACGGTTCTT | Xba1 |

**Supplemental Table 1. The list of primers used for plasmid construction.** Primer sequences were derived from *L. major*.
