# Supplementary material for: The eIF3 complex of Leishmania—subunit composition and mode of recruitment to different cap-binding complexes
Source: Nucleic Acids Res. 2015 Jun 19;43(13):6222–35. doi: 10.1093/nar/gkv564 (PMC4513851; doi:10.1093/nar/gkv564)
Supplement: SUPPLEMENTARY DATA [file supp_gkv564_nar-01173-v-2015-File010.pdf]

Supplemental Table 2. **The complete list of proteins pulled down by tagged LeishIF3e**. The list includes the protein identified in LC-MS/MS analysis of proteins complex pulled down by tagged LeishIF3e . The relative abundance of proteins are represented by peptide peak area and relative PAF values. The PAF values were determined by normalizing the peptide count of each protein to its molecular weight. The relative PAF was calculated for each protein by normalization to the PAF value of the bait protein. Proteins represented by less than two peptides were not included. All values were subjected to background substraction using the peptide count from a mock purification with a cell line expressing the tagged luciferase protein. The proteins were clustered according to their function. The mean relative PAF of three independant experiments is given along with its standard deviation.

Accession Number	Protein	Mean Relative PAF	STDEV	Mean Area	Mol.Wt
**LeishIF3 subunits**					
**LmjF.28.2310**	**eukaryotic translation initiation factor 3e**	**1.00**	0.00	1.518E10	46.35
**LmjF.07.0640**	**eukaryotic translation initiation factor 3h**	**0.78**	0.07	1.228E9	37.93
**LmjF.25.1610**	**eukaryotic translation initiation factor 3f**	**0.68**	0.02	1.964E9	36.71
**LmjF.32.2180**	**eukaryotic translation initiation factor 3k**	**0.67**	0.04	1.052E9	26.30
**LmjF.17.0010**	**eukaryotic translation initiation factor 3a**	**0.65**	0.13	3.381E9	87.57
**LmjF.17.1290**	**eukaryotic translation initiation factor 3b**	**0.59**	0.10	3.381E9	80.71
**LmjF.36.0250**	**eukaryotic translation initiation factor 3l**	**0.49**	0.07	2.313E9	72.60
**LmjF.34.2700**	**eukaryotic translation initiation factor 3g**	**0.48**	0.20	1.619E9	28.81
**LmjF.36.6980**	**eukaryotic translation initiation factor 3c**	**0.47**	0.08	5.066E9	81.98
**LmjF.30.3040**	**eukaryotic translation initiation factor 3d**	**0.42**	0.06	2.457E9	60.58
**LmjF.36.3880**	**eukaryotic translation initiation factor 3i**	**0.34**	0.01	1.838E9	45.41
**LmjF.25.2120**	**eukaryotic translation initiation factor 3j**	**0.13**	0.19	1.084E8	23.50

**Other initiation factors**					
**LmjF.24.1210**	**translation factor sui1, putative**	**0.51**	0.15	8.672E8	12.4
**LmjF.16.0140**	**eukaryotic translation initiation factor 1A, putative**	**0.34**	0.05	7.615E7	18.7
**LmjF.03.0980**	**eukaryotic initiation factor 2a, putative**	**0.25**	0.18	1.394E8	49.0
**LmjF.09.1070**	**eukaryotic translation initiation factor 2 subunit, putative**	**0.11**	0.06	8.968E7	52.6
**LmjF.34.0350**	**eukaryotic translation initiation factor 5, putative**	**0.19**	0.14	2.259E7	42.9
**LmjF.08.0550**	**eukaryotic translation initiation factor 2 beta subunit, putative**	**0.17**	0.13	5.194E7	38.0
LmjF.36.0890	eukaryotic translation initiation factor 6 (eIF-6), putative	0.14	0.11	2.692E7	27.1
LmjF.35.5040	polyadenylate-binding protein 1 (PABP1)	0.07	0.03	2.411E7	62.6
LmjF.25.0730	eukaryotic initiation factor 5a, putative (EIF5A2)	0.05	0.09	1.634E8	17.8
LmjF.30.0450	eukaryotic translation initiation factor 4e, putative	0.04	0.04	3.777E6	33.8
LmjF.16.1600	eukaryotic translation initiation factor 4 gamma, putative (EIF4G3)	0.02	0.01	6.824E6	71.2
LmjF.01.0770	eukaryotic initiation factor 4a, putative	0.02	0.14	5.488E8	45.3
**Elongation Factors**					
LmjF.34.0820	elongation factor 1-beta (eEF1B beta 1)	0.32	0.18	2.243E8	25.6
LmjF.36.0180	elongation factor 2 (EF2-1)	0.25	0.24	1.115E9	94.1
LmjF.18.0740	elongation factor Tu, putative	0.19	0.13	4.663E7	51.5
LmjF.17.0084	elongation factor 1-alpha	0.15	0.20	4.971E9	49.1
LmjF.09.0970	elongation factor-1 gamma (EF1G)	0.12	0.08	5.206E8	46.2
LmjF.36.0570	Mitochondrial elongation factor G, putative (MEFG)	0.03	0.04	1.247E7	84.2
**Ribosomal Proteins**					
LmjF.36.0940	40S ribosomal protein S18, putative	0.34	0.23	2.435E8	17.4
LmjF.29.2860	40S ribosomal protein S19-like protein	0.32	0.11	3.003E8	18.1
LmjF.28.1030	40S ribosomal protein S20	0.31	0.12	3.871E7	13.0
LmjF.36.1250	40S ribosomal protein S9, putative	0.29	0.14	2.437E8	22.1
LmjF.21.1550	40S ribosomal protein S11, putative	0.26	0.21	1.610E8	16.3
LmjF.28.2560	40S ribosomal protein S17, putative	0.24	0.22	1.332E8	16.5
LmjF.15.1470	ribosomal protein S6, putative	0.24	0.17	1.785E7	13.5

LmjF.24.2070	40S ribosomal protein S8, putative	0.22	0.08	1.114E8	24.9
LmjF.25.1190	ribosomal protein S25	0.21	0.12	5.528E7	13.0
LmjF.36.5010	40S ribosomal protein SA, putative	0.18	0.05	6.267E8	27.5
LmjF.36.0980	40S ribosomal protein S10, putative	0.16	0.08	3.758E7	18.6
LmjF.01.0420	ribosomal protein S7, putative	0.15	0.18	4.578E8	23.7
LmjF.35.2010	40S ribosomal protein S6, putative	0.15	0.16	2.910E8	28.3
LmjF.32.0450	40S ribosomal protein S2	0.15	0.16	4.211E8	28.6
LmjF.33.0920	40S ribosomal protein S3, putative	0.15	0.21	2.764E8	24.5
LmjF.21.1070	40S ribosomal protein S23, putative	0.14	0.20	2.464E8	15.9
LmjF.11.1190	40S ribosomal protein S15A, putative	0.14	0.08	2.269E8	14.7
LmjF.19.0390	40S ribosomal protein S13, putative	0.14	0.21	3.391E8	17.4
LmjF.36.0600	ubiquitin/ribosomal protein S27a, putative	0.12	0.13	1.550E8	17.0
LmjF.11.0970	40S ribosomal protein S5	0.11	0.12	2.636E8	21.3
LmjF.11.0760	40S ribosomal protein S21, putative	0.11	0.04	2.018E8	17.0
LmjF.35.0420	40S ribosomal protein S3A, putative	0.11	0.27	5.612E8	30.0
LmjF.26.0890	40S ribosomal protein S16, putative	0.08	0.22	3.165E8	16.7
LmjF.22.0420	40S ribosomal protein S15, putative	0.08	0.12	1.027E8	17.4
LmjF.13.1230	40S ribosomal protein S4, putative	0.06	0.29	8.281E8	30.7
LmjF.31.2030	ubiquitin-fusion protein(ubiquitin 60S ribosomal protein L40)	0.54	0.19	2.549E8	14.7
LmjF.35.3280	60S ribosomal subunit protein L31, putative	0.49	0.23	2.450E8	21.2
LmjF.04.0950	60S ribosomal protein L10, putative	0.40	0.31	2.833E8	24.5
LmjF.13.1670	60S ribosomal protein L44, putative	0.39	0.24	4.347E7	12.3
LmjF.33.0720	60S ribosomal protein L6, putative	0.38	0.18	1.628E8	21.1
LmjF.36.3740	60S ribosomal protein L34, putative	0.37	0.23	1.036E8	19.3
LmjF.32.2690	ribosomal protein L27, putative	0.37	0.28	3.972E8	15.4
LmjF.26.2220	ribosomal protein L38, putative	0.35	0.26	2.110E7	9.4
LmjF.29.2460	60S ribosomal protein L13, putative	0.33	0.31	4.724E8	24.7
LmjF.10.0070	ribosomal protein l35a, putative	0.31	0.60	2.344E8	16.4
LmjF.36.3390	ribosomal protein L29, putative	0.31	0.17	5.599E7	8.0
LmjF.26.0170	60S ribosomal protein L7, putative	0.31	0.19	1.862E8	28.8
LmjF.35.1920	60S Ribosomal protein L36, putative	0.26	0.32	2.690E8	11.9
LmjF.13.0560	60S ribosomal protein L18, putative	0.26	0.24	2.752E8	22.0
LmjF.35.1910	ribosomal protein L15, putative	0.24	0.13	8.493E7	24.0
LmjF.27.1390	60S acidic ribosomal subunit protein, putative	0.24	0.10	3.259E8	34.8

LmjF.06.0415	60S ribosomal protein L19, putative	0.23	0.10	3.165E8	28.8
LmjF.11.1130	60S ribosomal protein L28, putative	0.22	0.12	1.830E8	16.3
LmjF.34.2880	ribosomal protein L3, putative	0.22	0.27	5.080E8	47.5
LmjF.33.1955	60S ribosomal protein L37	0.21	0.25	3.164E7	9.8
LmjF.21.1720	60S ribosomal protein L32 (RPL32)	0.21	0.13	1.794E8	15.4
LmjF.36.1925	60S ribosomal protein L37a	0.20	0.24	1.103E8	10.3
LmjF.36.1100	ribosomal protein L24, putative	0.20	0.11	4.998E7	14.6
LmjF.35.1890	60S ribosomal protein L5, putative	0.20	0.17	4.834E8	36.6
LmjF.04.0470	60S ribosomal protein L11 (L5, L16)	0.20	0.09	2.191E8	21.6
LmjF.22.1560	60S ribosomal protein L14, putative	0.20	0.16	1.517E8	19.9
LmjF.07.0510	60S ribosomal protein L7a, putative	0.17	0.19	2.331E8	29.7
LmjF.24.2210	60S ribosomal protein L12, putative	0.15	0.06	1.561E8	17.5
LmjF.29.1080	ribosomal protein L1a, putative	0.15	0.22	2.505E8	41.1
LmjF.06.0570	60S ribosomal protein L23a, putative	0.15	0.33	2.455E8	16.3
LmjF.26.2330	60S ribosomal protein L35, putative	0.12	0.17	2.346E8	15.2
LmjF.35.3760	60S ribosomal protein L27A/L29, putative	0.11	0.18	1.158E8	16.1
LmjF.16.0460	60S ribosomal protein L21, putative	0.10	0.20	3.696E8	18.0
LmjF.35.3790	60S ribosomal protein L23, putative	0.09	0.36	3.292E8	14.9
LmjF.35.1670	60S ribosomal protein L26, putative	0.08	0.17	3.038E8	16.2
LmjF.35.1440	60S ribosomal protein L2, putative	0.08	0.30	5.061E8	28.3
LmjF.35.0240	60S ribosomal protein L30	0.08	0.36	6.677E7	11.3
LmjF.15.0200	60S ribosomal protein L13a, putative	0.02	0.26	2.514E8	25.4
LmjF.21.1050	60S ribosomal protein L9, putative	0.01	0.15	3.463E8	21.5
**tRNA synthetase**					
LmjF.27.1310	arginyl-tRNA synthetase, putative	0.26	0.15	1.453E8	78.20
LmjF.30.0460	aspartyl-tRNA synthetase, putative	0.15	0.09	7.165E7	62.33
LmjF.15.0230	lysyl-tRNA synthetase, putative	0.13	0.08	4.517E7	66.99
LmjF.11.0100	seryl-tRNA synthetase, putative	0.12	0.10	1.850E8	53.05
LmjF.15.1440	glutaminyl-tRNA synthetase, putative	0.11	0.09	4.380E7	66.03
LmjF.13.1100	leucyl-tRNA synthetase, putative	0.11	0.09	5.104E7	121.98
LmjF.36.3840	glycyl tRNA synthetase, putative	0.09	0.06	4.956E7	70.29
LmjF.22.1540	alanyl-tRNA synthetase, putative	0.08	0.06	4.028E7	106.25

LmjF.14.1370	tyrosyl-tRNA synthetase, putative (TyrS)	0.04	0.03	7.357E6	74.90
**RNA binding protein**					
LmjF.32.0750	RNA binding protein, putative	0.21	0.11	4.198E7	25.17
LmjF.23.0760	mitochondrial RNA binding protein, putative (RBP38)	0.16	0.07	1.836E7	39.71
LmjF.15.1380	nucleolar RNA binding protein, putative	0.14	0.03	3.447E7	66.01
LmjF.07.0990	nucleolar RNA-binding protein, putative	0.08	0.03	6.195E7	37.55
LmjF.21.0540	la RNA binding protein, putative	0.07	0.02	1.379E7	37.25
LmjF.04.1170	RNA-binding protein, putative	0.06	0.05	8.605E7	34.50
**Chaperones**					
LmjF.24.1500	translationally controlled tumor protein (TCTP), putative	0.45	0.03	2.041E8	0.12
LmjF.30.2550	heat shock 70-related protein 1, mitochondrial precursor, putative	0.33	0.19	1.603E9	1.54
LmjF.21.1090	T-complex protein 1, delta subunit, putative	0.27	0.16	2.275E8	0.30
LmjF.08.1110	stress-induced protein sti1	0.25	0.16	1.102E8	0.11
LmjF.36.0070	stress-inducible protein STI1 homolog	0.23	0.14	8.146E7	0.12
LmjF.33.0316	heat shock protein 83 (HSP83-3)	0.23	0.14	1.873E9	2.07
LmjF.13.1660	chaperonin TCP20, putative	0.22	0.14	1.307E8	0.13
LmjF.32.3270	chaperonin alpha subunit, putative	0.21	0.12	1.732E8	0.22
LmjF.32.1000	chaperonin containing t-complex protein, putative	0.21	0.19	2.174E8	0.18
LmjF.23.0050	cyclophilin 11, putative (CYP11)	0.20	0.05	1.380E8	0.02
LmjF.26.0660	protein disulfide isomerase, putative	0.17	0.00	9.657E7	0.11
LmjF.26.1380	prefoldin-like protein	0.16	0.05	6.793E7	0.09
LmjF.30.0730	co-chaperone GrpE, putative	0.16	0.10	9.411E7	0.19
LmjF.25.0910	cyclophilin a (CYPA)	0.16	0.02	2.713E8	0.53
LmjF.31.0050	cyclophilin 5, putative (CYP5)	0.16	0.08	1.893E7	0.02
LmjF.28.1200	glucose-regulated protein 78, putative	0.16	0.14	5.459E8	0.64
LmjF.33.2390	heat shock protein, putative	0.15	0.14	1.105E8	0.10
LmjF.06.0120	cyclophilin 2 (CYP2)	0.15	0.06	1.539E8	0.09
LmjF.22.0670	prefoldin 5-like protein	0.14	0.10	8.495E6	0.02
LmjF.36.6270	chaperone protein DNAj, putative	0.13	0.06	1.833E7	0.03

LmjF.26.1240	heat shock protein 70-related protein (HSP70.4)	0.13	0.11	1.055E9	0.24
LmjF.18.1370	heat shock protein 110, putative	0.08	0.09	2.843E8	0.41
LmjF.15.0090	heat shock protein HslVU, ATPase subunit HslU, putative	0.08	0.14	3.997E7	0.01
LmjF.04.0940	chaperone protein DNAj, putative	0.08	0.01	1.301E7	0.01
LmjF.19.1530	FK506-binding protein (FKBP)-type peptidyl-prolyl isomerase, putative	0.08	0.04	2.532E7	0.03
LmjF.09.0230	heat shock protein HslVU, ATPase subunit HslU, putative (HSLU)	0.07	0.04	2.654E7	0.04
LmjF.09.0220	catalytic subunit of the vacuolar transporter chaperone 4, putative	0.06	0.03	2.637E7	0.02
LmjF.36.0500	DNAJ domain protein, putative	0.05	0.02	7.194E6	0.01
LmjF.24.0520	DNAJ domain protein, putative	0.05	0.03	2.881E7	0.03
**RNA helicases**					
LmjF.34.2610	ATP-dependent DNA helicase, putative	0.18	0.12	6.429E7	53.60
LmjF.21.1552	RNA helicase, putative	0.16	0.15	6.855E7	49.46
LmjF.32.0400	ATP-dependent RNA helicase, putative	0.15	0.13	3.088E8	66.97
LmjF.35.3100	ATP-dependent RNA helicase, putative	0.15	0.10	1.264E8	100.18
LmjF.28.1530	ATP-dependent RNA helicase FAL1, putative	0.09	0.08	1.224E7	43.97
LmjF.36.2130	DEAD box RNA helicase, putative	0.05	0.03	1.169E7	63.09
**Cytoskelton**					
LmjF.34.1520	hypothetical protein, conserved (tubulin polymerisation promoting protein)	0.73	0.07	1.720E8	16.6
LmjF.13.0280	alpha tubulin	0.19	0.13	2.849E8	49.7
LmjF.04.1230	actin (ACT)	0.18	0.07	7.219E8	42.0
LmjF.21.1760	centromere/microtubule binding protein cbf5, putative	0.17	0.09	5.967E7	48.4
LmjF.16.1425	paraflagellar rod protein 2C	0.10	0.06	2.199E7	68.7
LmjF.24.0140	ankyrin/TPR repeat protein	0.10	0.06	1.456E7	42.9
LmjF.23.0560	kinesin, putative	0.09	0.05	2.277E7	75.4
LmjF.19.0680	kinesin, putative	0.04	0.02	2.207E7	119.0
LmjF.13.1610	MCAK-like kinesin, putative	0.04	0.02	1.784E7	80.8
LmjF.32.3870	myosin XXI	0.03	0.02	1.181E7	119.0

Protein Degradation Machinery

LmjF.04.0680	ubiquitin-conjugating enzyme e2, putative	0.42	0.13	7.102E7	17.0
LmjF.13.1580	ubiquitin-conjugating enzyme-like protein	0.25	0.10	5.068E7	16.1
LmjF.35.1300	ubiquitin-conjugating enzyme e2, putative	0.22	0.07	4.781E7	16.6
LmjF.21.1700	proteasome alpha 2 subunit, putative	0.19	0.04	6.354E7	25.1
LmjF.19.0160	aminopeptidase, putative	0.17	0.11	1.312E8	42.5
LmjF.14.0310	proteasome alpha 3 subunit, putative	0.17	0.08	4.598E7	32.1
LmjF.36.3990	hs1vu complex proteolytic subunit-like, threonine peptidase, Clan T(1), family T1B	0.14	0.10	4.788E7	23.3
LmjF.13.0090	carboxypeptidase, putative	0.14	0.06	4.542E7	57.0
LmjF.32.2820	proteasome regulatory non-ATP-ase subunit, putative	0.13	0.06	4.386E7	46.3
LmjF.33.2540	carboxypeptidase, putative	0.12	0.10	4.734E7	57.0
LmjF.32.0390	26S proteasome regulatory subunit, putative	0.10	0.07	4.182E7	40.1
LmjF.35.3840	proteasome beta 2 subunit, putative	0.10	0.07	1.867E7	27.5
LmjF.22.0620	proteasome regulatory ATPase subunit 5, putative	0.09	0.06	1.570E7	49.5
LmjF.35.4850	proteasome alpha 1 subunit, putative	0.09	0.07	1.029E7	27.2
LmjF.23.0550	ubiquitin-activating enzyme e1, putative	0.09	0.05	3.668E7	115.2
LmjF.21.1830	20S proteasome subunit alpha 5, (putative)	0.08	0.03	3.958E7	26.8
LmjF.02.0370	proteasome regulatory non-ATPase subunit 6, putative	0.08	0.07	2.645E7	58.8
LmjF.36.1600	proteasome alpha 1 subunit, putative	0.08	0.01	2.598E7	29.6
LmjF.11.0240	proteasome alpha 7 subunit, putative	0.07	0.07	6.317E7	27.8
LmjF.12.0210	proteasome regulatory ATPase subunittcc1l8.3, putative	0.07	0.06	6.389E7	45.8
LmjF.33.1610	peptidase M20/M25/M40, putative	0.06	0.05	3.031E7	51.5
LmjF.02.0710	ATP-dependent Clp protease subunit, heat shock protein 78 (HSP78), putative	0.06	0.05	2.651E7	90.8
LmjF.35.2350	aminopeptidase P, putative	0.06	0.05	2.280E7	53.6
LmjF.34.4400	20s proteasome beta 7 subunit, (putative)	0.06	0.07	5.659E7	24.7
LmjF.05.0960	metallo-peptidase, Clan M-, Family M49	0.05	0.05	2.018E7	75.9
LmjF.13.1090	proteasome regulatory ATPase subunit 2, putative	0.05	0.06	1.540E7	49.3
LmjF.35.3060	ubiquitin-activating enzyme e1, putative	0.04	0.03	1.842E7	126.4
LmjF.21.0760	proteasome regulatory non-ATP-ase subunit 5, putative	0.04	0.13	3.593E7	54.2
LmjF.20.1185	calpain-like cysteine peptidase, putative	0.04	0.02	2.507E7	91.3
LmjF.27.1460	proteasome regulatory non-ATP-ase subunit 3, putative	0.03	0.04	2.416E7	37.9

LmjF.29.1270	ATP-dependent Clp protease subunit, heat shock protein 104 (HSP104), putative,atp-dependent chaperone	0.02	0.03	1.092E7	96.9
LmjF.29.2240	aminopeptidase, putative	0.02	0.03	9.644E6	96.3
LmjF.19.1120	proteasome regulatory non-ATP-ase subunit, putative	0.01	0.02	1.213E7	46.5
**Metabolic enzymes**					
LmjF.26.1710	cytochrome c oxidase subunit V, putative	0.45	0.11	4.944E7	22.3
LmjF.35.0140	hypothetical protein, conserved (metabolic enzyme)	0.39	0.06	6.956E7	14.4
LmjF.25.2130	succinyl-CoA synthetase alpha subunit, putative	0.37	0.06	5.071E8	30.9
LmjF.28.2420	2-oxoglutarate dehydrogenase, E2 component, dihydrolipoamide succinyltransferase, putative	0.31	0.08	5.858E8	41.7
LmjF.36.3100	ATP synthase, putative	0.29	0.09	2.756E7	25.1
LmjF.32.3610	hypothetical protein, conserved (mannose 6 phosphate isomerase	0.28	0.06	5.958E7	16.8
LmjF.10.0290	isocitrate dehydrogenase [NADP], mitochondrial precursor, putative	0.27	0.08	1.640E8	48.5
LmjF.24.2110	hypothetical protein, conserved(3-hydroxy-3-methylglutaryl-CoA synthase)	0.27	0.10	1.975E8	55.2
LmjF.14.0190	hypothetical protein, conserved (thiol -sulfide oxidoreductase)	0.26	0.08	8.619E7	22.3
LmjF.35.1010	casein kinase, putative	0.25	0.10	3.664E7	39.7
LmjF.16.0550	orotidine-5-phosphate decarboxylase/orotate phosphoribosyltransferase, putative	0.24	0.06	6.417E7	49.5
LmjF.35.0100	hypothetical protein, conserved (ubiquinol-cytochrome-c reductase)	0.23	0.15	7.104E7	24.2
LmjF.34.0110	adenylate kinase, putative	0.23	0.03	3.160E7	24.4
LmjF.22.0770	NADH-cytochrome b5 reductase, putative	0.23	0.06	7.018E7	31.8
LmjF.36.3910	S-adenosylhomocysteine hydrolase	0.22	0.17	3.828E8	47.8
LmjF.21.0240	hexokinase, putative	0.22	0.12	2.443E8	51.7
LmjF.03.0200	delta-1-pyrroline-5-carboxylate dehydrogenase, putative	0.21	0.11	8.257E7	61.9
LmjF.11.1030	hypothetical protein, conserved (3-isopropylmalate dehydrogenase)	0.21	0.08	3.650E7	27.5
LmjF.35.1230	short chain dehydrogenase, putative	0.20	0.06	1.852E8	28.1
LmjF.36.6995	cytochrome c oxidase subunit I	0.20	0.03	2.858E7	13.9
LmjF.18.1380	pyruvate dehydrogenase E1 component alpha subunit, putative	0.20	0.07	3.748E7	42.8
LmjF.23.1480	alanine racemase, putative	0.19	0.04	2.048E7	27.1

LmjF.27.1805	glycosomal phosphoenolpyruvate carboxykinase, putative	0.19	0.08	2.290E8	58.2
LmjF.29.2510	ATP-dependent phosphofructokinase	0.18	0.11	1.069E8	54.0
LmjF.30.3520	S-adenosylmethionine synthetase (METK2)	0.18	0.03	3.834E8	43.0
LmjF.31.2150	prostaglandin f2-alpha synthase/D-arabinose dehydrogenase (PGFS)	0.18	0.11	7.535E7	31.8
LmjF.29.0760	lipophosphoglycan biosynthetic protein, putative (LPG3)	0.17	0.10	1.047E8	86.6
LmjF.20.0110	phosphoglycerate kinase B, cytosolic (PGKB)	0.17	0.11	7.804E7	44.9
LmjF.19.0710	glycosomal malate dehydrogenase (gMDH)	0.17	0.08	1.096E8	33.6
LmjF.19.1560	inosine-5'-monophosphate dehydrogenase	0.17	0.09	7.818E7	55.5
LmjF.35.0030	pyruvate kinase	0.16	0.10	1.151E8	54.2
LmjF.25.2010	2,4-dihydroxyhept-2-ene-1,7-dioic acid aldolase, putative	0.16	0.05	1.702E8	30.3
LmjF.33.2340	succinyl-coA:3-ketoacid-coenzyme A transferase, mitochondrial precursor, putative	0.16	0.11	5.153E7	52.5
LmjF.27.2020	D-lactate dehydrogenase-like protein	0.15	0.09	5.493E7	53.7
LmjF.30.3380	PAS-domain containing phosphoglycerate kinase, putative	0.15	0.14	2.310E7	57.5
LmjF.30.3600	ATP synthase, epsilon chain, putative	0.15	0.06	1.073E7	20.1
LmjF.18.0510	aconitase, putative	0.15	0.08	3.706E8	97.4
LmjF.12.0530	glucose-6-phosphate isomerase (PGI)	0.15	0.08	7.324E7	67.1
LmjF.15.1010	glutamate dehydrogenase (GDH)	0.14	0.09	1.862E8	115.0
LmjF.22.1290	ribonucleoside-diphosphate reductase small chain, putative	0.14	0.05	3.813E7	44.4
LmjF.35.0330	3-keto-dihydrosphingosine reductase	0.14	0.03	3.179E7	35.8
LmjF.24.2030	3-oxoacyl-(acyl-carrier protein) reductase, putative	0.14	0.06	3.125E7	27.0
LmjF.15.0990	succinate dehydrogenase, putative	0.14	0.05	6.799E7	22.5
LmjF.29.1310	carnitine/choline acetyltransferase, putative	0.14	0.07	3.487E7	68.6
LmjF.28.1840	methylthioribulose-1-phosphate dehydratase, putative	0.14	0.06	9.315E6	27.2
LmjF.27.0880	2-oxoglutarate dehydrogenase subunit, putative	0.14	0.07	2.017E8	112.6
LmjF.25.1130	cytochrome c oxidase VII, putative	0.13	0.06	3.006E7	19.1
LmjF.24.1840	lysophospholipase, putative	0.13	0.04	1.271E7	30.3
LmjF.13.1680	pyrroline-5-carboxylate reductase (P5CR)	0.13	0.01	6.559E7	28.7
LmjF.35.1180	NADH-dependent fumarate reductase, putative	0.13	0.07	6.354E7	123.1
LmjF.36.5590	CAP/Srv2p, putative	0.13	0.05	2.342E7	29.3
LmjF.04.0960	adenylate kinase, putative	0.13	0.08	1.778E7	23.4
LmjF.21.1770	ATP synthase F1 subunit gamma protein, putative	0.12	0.08	2.038E8	34.4
LmjF.36.3470	2-oxoglutarate dehydrogenase E1 component, putative	0.12	0.03	1.808E8	111.0
LmjF.33.1330	cysteine conjugate beta-lyase, aminotransferase- like protein	0.12	0.05	1.148E7	45.8

LmjF.13.1060	NADH-cytochrome b5 reductase, putative	0.12	0.09	2.828E7	34.7
LmjF.36.1140	short chain 3-hydroxyacyl-CoA dehydrogenase, putative	0.12	0.06	1.677E7	33.0
LmjF.21.1430	2-oxoisovalerate dehydrogenase alpha subunit, putative	0.12	0.05	6.107E7	53.3
LmjF.24.0770	malic enzyme, putative	0.12	0.08	8.608E7	63.4
LmjF.28.2510	acyl-CoA dehydrogenase, putative	0.12	0.09	4.356E7	68.8
LmjF.34.0140	malate dehydrogenase	0.11	0.04	1.211E8	33.3
LmjF.24.1630	succinate dehydrogenase flavoprotein, putative	0.11	0.08	3.851E7	66.7
LmjF.28.2860	cytosolic malate dehydrogenase (cMDH)	0.11	0.07	1.981E7	34.1
LmjF.31.0010	5-methyltetrahydropteroyltriglutamate-homocysteine S-methyltransferase	0.11	0.04	1.053E8	86.1
LmjF.35.5330	isopentenyl-diphosphate delta-isomerase (type II), putative	0.11	0.06	3.226E7	39.5
LmjF.32.3310	dihydrolipoamide dehydrogenase, putative (GCVL-2)	0.11	0.13	4.800E7	50.5
LmjF.16.1320	cytochrome c, putative	0.11	0.07	8.331E7	12.2
LmjF.35.1480	arginase	0.10	0.04	1.442E8	36.1
LmjF.35.3230	cystathione gamma lyase, putative	0.10	0.10	1.962E8	44.5
LmjF.05.0830	methylthioadenosine phosphorylase, putative	0.10	0.05	8.797E7	33.4
LmjF.24.0370	aspartate aminotransferase, putative	0.10	0.06	1.887E7	47.6
LmjF.25.1120	aldehyde dehydrogenase, mitochondrial precursor (ALDH2)	0.10	0.08	7.617E7	54.1
LmjF.18.1520	P-type H -ATPase, putative (H1A-2)	0.10	0.05	5.144E7	106.9
LmjF.12.0670	cytochrome c oxidase subunit IV	0.10	0.10	7.517E7	39.4
LmjF.25.1710	pyruvate dehydrogenase E1 beta subunit, putative	0.10	0.07	4.228E7	37.9
LmjF.05.0500	ATPase alpha subunit	0.09	0.14	4.508E8	62.5
LmjF.18.0670	citrate synthase, putative	0.09	0.05	1.049E8	50.4
LmjF.14.0650	fatty acid elongase, putative (ELO1.2)	0.09	0.03	7.774E7	32.3
LmjF.36.2660	dihydrolipoamide acetyltransferase precursor, putative	0.09	0.04	7.153E7	48.7
LmjF.36.4170	oxidoreductase, putative	0.09	0.08	1.432E7	36.1
LmjF.36.1960	phosphomannomutase, putative (PMM)	0.09	0.07	4.023E7	28.1
LmjF.20.0100	phosphoglycerate kinase C, glycosomal (PGKC)	0.09	0.11	8.259E7	51.5
LmjF.11.1100	sterol 14-alpha-demethylase, putative (CYP51)	0.09	0.07	2.751E7	54.1
LmjF.33.1090	guanylate kinase, putative	0.09	0.07	2.685E7	22.9
LmjF.21.1710	cytochrome c oxidase subunit VI, putative (COX6)	0.08	0.06	1.189E7	19.2
LmjF.29.1960	fumarate hydratase, putative	0.08	0.09	3.468E7	62.6
LmjF.33.2550	isocitrate dehydrogenase, putative	0.08	0.06	5.236E7	46.3
LmjF.32.1820	iron superoxide dismutase, putative (SODB1)	0.08	0.04	8.942E7	21.5
LmjF.23.0200	endoribonuclease L-PSP (pb5), putative	0.08	0.01	1.495E8	16.9

LmjF.20.1570	N-acyl-L-amino acid amidohydrolase, putative	0.08	0.04	2.754E7	45.1
LmjF.36.6650	2,3-bisphosphoglycerate-independent phosphoglycerate mutase (PGAM)	0.08	0.04	5.052E7	60.7
LmjF.03.0600	arginine N-methyltransferase, putative	0.08	0.07	2.234E7	44.4
LmjF.16.0950	sucrose-phosphate synthase-like protein	0.08	0.06	5.339E7	52.4
LmjF.36.3410	short chain dehydrogenase-like protein	0.08	0.04	2.131E7	40.7
LmjF.01.0050	carboxylase, putative	0.07	0.06	7.934E7	73.7
LmjF.36.3590	cysteine synthase	0.07	0.08	3.994E7	35.4
LmjF.28.0890	ribonucleoside-diphosphate reductase large chain, putative	0.07	0.08	5.680E7	90.7
LmjF.16.0530	dihydroorotate dehydrogenase (fumarate) (DHODH)	0.07	0.02	1.383E8	34.6
LmjF.06.1270	coproporphyrinogen III oxidase	0.07	0.02	2.434E7	34.3
LmjF.28.0490	propionyl-coa carboxylase beta chain, putative	0.06	0.02	3.552E7	56.7
LmjF.20.0560	cytidine triphosphate synthase, putative	0.06	0.07	2.972E7	65.6
LmjF.01.0520	long-chain-fatty-acid-CoA ligase, putative	0.06	0.05	3.403E7	77.6
LmjF.17.0725	guanosine monophosphate reductase	0.06	0.04	2.163E7	58.8
LmjF.28.1140	electron-transfer-flavoprotein, alpha polypeptide, putative	0.06	0.06	2.067E7	33.5
LmjF.01.0490	long-chain-fatty-acid-CoA ligase, putative	0.06	0.04	3.932E7	76.9
LmjF.06.0860	dihydrofolate reductase-thymidylate synthase (DHFR-TS)	0.06	0.01	1.768E7	58.7
LmjF.35.0820	aspartate aminotransferase, putative	0.06	0.01	4.404E7	46.0
LmjF.17.1400	otubain cysteine peptidase, Clan CA, family C65, putative	0.06	0.04	1.129E7	30.2
LmjF.18.0270	glycogen synthase kinase 3, putative (GSK3)	0.05	0.06	2.102E7	40.7
LmjF.07.0060	cytochrome c1, heme protein, mitochondrial precursor, putative	0.05	0.06	7.164E7	29.9
LmjF.14.0130	inosine-guanine nucleoside hydrolase, putative (IG-NH)	0.05	0.03	8.724E6	39.0
LmjF.28.1280	phenylalanine-4-hydroxylase (PAH)	0.05	0.02	3.273E7	51.4
LmjF.30.0120	alkyldihydroxyacetonephosphate synthase (ADS1)	0.04	0.06	2.297E7	69.5
LmjF.14.1360	inositol-3-phosphate synthase (INO1)	0.04	0.03	1.938E7	58.3
LmjF.21.0640	phosphoglucomutase, putative	0.04	0.03	4.439E7	63.9
LmjF.07.0460	acyl-CoA dehydrogenase, mitochondrial precursor, putative	0.04	0.04	5.398E6	56.4
LmjF.14.1320	serine hydroxymethyltranferase (SHMT-S) (SHMT-S)	0.03	0.05	6.227E6	50.6
LmjF.35.0830	NADH-dependent fumarate reductase-like protein	0.03	0.02	4.079E7	129.7
LmjF.35.1140	oligosaccharyl transferase-like protein	0.02	0.02	2.937E7	85.4
**Hypothetical proteins**					
LmjF.04.0630	hypothetical protein, conserved	0.19	0.16	6.788E7	23.4

LmjF.33.1070	hypothetical protein, unknown function	0.16	0.06	2.878E7	27.7
LmjF.28.0930	hypothetical protein, conserved	0.15	0.06	2.008E8	45.5
LmjF.20.0240	hypothetical protein, conserved	0.15	0.06	3.263E7	29.1
LmjF.32.0950	hypothetical protein, conserved	0.15	0.08	5.825E7	102.2
LmjF.09.0840	hypothetical protein, conserved	0.14	0.06	5.152E7	42.5
LmjF.25.2020	hypothetical protein, conserved	0.14	0.09	7.278E7	32.8
LmjF.04.0123	hypothetical protein, conserved	0.14	0.10	1.818E7	16.5
LmjF.30.1040	hypothetical protein, conserved	0.14	0.04	2.895E7	29.1
LmjF.22.0180	hypothetical protein, conserved	0.13	0.06	3.220E7	28.2
LmjF.26.0680	hypothetical protein, conserved	0.13	0.04	4.219E7	25.6
LmjF.27.1300	hypothetical protein, conserved	0.13	0.12	4.840E7	59.9
LmjF.35.1460	hypothetical protein, conserved	0.12	0.03	9.197E6	35.8
LmjF.36.0480	hypothetical protein, conserved	0.12	0.04	3.266E7	26.1
LmjF.30.3150	hypothetical protein, conserved	0.11	0.08	1.634E7	23.3
LmjF.30.0560	hypothetical protein, conserved	0.11	0.01	1.646E7	36.7
LmjF.16.0520	hypothetical protein, conserved	0.11	0.03	5.658E7	36.9
LmjF.07.0290	hypothetical protein, conserved	0.10	0.04	8.055E6	27.1
LmjF.34.0010	hypothetical protein, conserved	0.10	0.01	9.101E7	33.6
LmjF.14.0970	hypothetical protein, conserved	0.10	0.05	3.036E7	31.6
LmjF.35.4470	hypothetical protein, conserved	0.10	0.05	2.822E8	21.5
LmjF.23.1410	hypothetical protein, conserved	0.10	0.01	3.009E7	27.4
LmjF.15.0040	hypothetical protein, conserved	0.09	0.08	1.965E7	37.7
LmjF.14.0450	hypothetical protein, conserved	0.09	0.07	3.855E7	36.9
LmjF.04.0420	hypothetical protein, conserved	0.09	0.04	1.348E7	39.2
LmjF.35.2150	hypothetical protein, conserved	0.09	0.07	4.727E7	48.1
LmjF.29.1100	hypothetical protein, conserved	0.09	0.08	2.061E7	40.1
LmjF.35.4880	hypothetical protein, conserved	0.09	0.08	1.787E7	42.7
LmjF.20.1290	hypothetical protein, conserved	0.08	0.05	9.017E6	31.4
LmjF.09.1010	hypothetical protein, conserved	0.08	0.05	6.496E7	67.2
LmjF.29.0870	hypothetical protein, conserved	0.08	0.06	4.785E7	53.4
LmjF.27.1680	hypothetical protein, conserved	0.08	0.05	2.510E7	58.9
LmjF.05.1100	hypothetical protein, conserved	0.07	0.06	1.162E7	28.2
LmjF.21.0430	hypothetical protein, conserved	0.07	0.07	1.305E7	44.7
LmjF.11.0880	hypothetical protein, conserved	0.07	0.05	1.804E7	37.5
LmjF.33.1590	hypothetical protein, conserved	0.06	0.05	1.220E7	36.1

LmjF.34.3360	hypothetical protein, conserved	0.06	0.07	1.770E7	23.6
LmjF.35.4860	hypothetical protein, conserved	0.06	0.07	5.081E7	38.5
LmjF.30.3690	hypothetical protein, conserved	0.06	0.05	1.581E7	66.4
LmjF.32.0840	hypothetical protein, conserved	0.06	0.03	1.149E8	57.4
LmjF.27.1250	hypothetical protein, conserved	0.06	0.08	2.012E7	35.3
LmjF.19.1550	hypothetical protein, conserved	0.06	0.05	1.823E7	43.4
LmjF.29.1470	hypothetical protein, conserved	0.05	0.05	1.562E7	75.0
LmjF.18.0700	hypothetical protein, conserved	0.05	0.02	1.365E7	77.3
LmjF.26.1310	hypothetical protein, conserved	0.05	0.08	1.390E7	38.8
LmjF.36.4040	hypothetical protein, conserved	0.05	0.04	9.256E6	47.0
LmjF.36.1750	hypothetical protein, conserved	0.05	0.02	2.189E7	59.4
LmjF.34.3190	hypothetical protein, conserved	0.04	0.04	6.570E6	55.4
LmjF.30.0660	hypothetical protein, conserved	0.03	0.02	2.168E7	79.9
LmjF.11.0480	hypothetical protein, conserved	0.03	0.02	1.442E7	117.9
LmjF.15.0690	hypothetical protein, conserved	0.03	0.03	9.419E6	53.5
LmjF.35.4710	hypothetical protein, conserved	0.03	0.02	1.766E7	78.8
LmjF.26.1900	hypothetical protein, conserved	0.02	0.01	6.515E6	134.0
LmjF.03.0690	hypothetical protein, conserved	0.01	0.01	1.659E7	246.9
**Miscellaneous**					
LmjF.16.1610	prohibitin	0.46	0.20	5.602E7	30.3
LmjF.35.0070	prohibitin, putative	0.31	0.24	4.451E7	32.3
LmjF.13.0450	hypothetical protein, conserved (alba domain protein)	0.28	0.08	5.585E7	13.3
LmjF.14.0850	small myristoylated protein-3, putative (SMP-3)	0.26	0.12	4.154E7	12.9
LmjF.19.0210	ADP,ATP carrier protein 1, mitochondrial precursor, putative (ANC2)	0.26	0.13	1.307E9	35.1
LmjF.31.2790	ADP-ribosylation factor, putative	0.26	0.15	1.380E8	20.2
LmjF.14.1040	hypothetical protein, conserved (vacoular transporter chaperon)	0.24	0.06	3.818E7	19.9
LmjF.35.1540	rieske iron-sulfur protein precursor, putative	0.24	0.16	1.190E8	33.7
LmjF.15.1450	proliferative cell nuclear antigen (PCNA), putative	0.23	0.10	4.509E7	32.4
LmjF.08.0030	Qc-SNARE protein, putative	0.22	0.01	2.703E7	24.2
LmjF.26.0820	type II (glutathione peroxidase-like) tryparedoxin peroxidase (TDPX)	0.21	0.09	3.092E7	19.3
LmjF.19.0100	fibrillarin, putative	0.21	0.05	2.150E7	31.0

LmjF.27.2000	hypothetical protein, conserved (protein unc-119 homolog)	0.20	0.03	4.504E7	22.3
LmjF.34.2580	hypothetical protein, conserved (ribonuclease protein P subunit p25	0.19	0.05	7.764E7	22.6
LmjF.35.1380	mitochondrial processing peptidase, beta subunit, putative	0.18	0.12	1.805E8	54.5
LmjF.28.2430	vacuolar ATP synthase subunit b, putative	0.17	0.14	1.914E7	55.5
LmjF.30.0970	p22 protein precursor, putative	0.17	0.05	2.985E7	22.0
LmjF.10.0850	nuclear transport factor 2, putative	0.17	0.04	1.510E7	13.9
LmjF.13.1480	Ran-binding protein 1, putative	0.17	0.03	4.749E7	17.8
LmjF.25.0750	protein phosphatase, putative (PP2C)	0.16	0.09	1.005E8	44.9
LmjF.10.0210	nucleolar protein, putative	0.16	0.07	4.102E7	52.7
LmjF.35.2220	kinetoplastid membrane protein-11 (KMPII-2)	0.15	0.14	3.953E7	11.2
LmjF.32.2020	cop-coated vesicle membrane protein p24 precursor	0.15	0.04	2.947E7	18.1
LmjF.29.0880	ADP ribosylation factor 3, putative	0.15	0.04	4.689E7	20.2
LmjF.05.0280	protein tyrosine phosphatase, putative	0.15	0.08	3.405E7	25.2
LmjF.14.0930	tc40 antigen-like	0.14	0.09	4.454E7	91.5
LmjF.30.3430	protein mkt1, putative	0.14	0.06	2.441E7	90.2
LmjF.21.0340	mitochondrial processing peptidase alpha subunit, putative	0.14	0.05	4.858E7	51.4
LmjF.34.3500	ruvb-like 1 DNA helicase, putative	0.13	0.07	5.951E7	50.3
LmjF.36.1370	Transitional endoplasmic reticulum ATPase, putative	0.12	0.16	7.834E7	86.8
LmjF.31.1070	biotin/lipoate protein ligase-like protein	0.12	0.06	2.637E7	28.3
LmjF.34.3670	vacuolar ATP synthase catalytic subunit A, putative	0.12	0.09	1.879E8	67.7
LmjF.05.1210	protein phosphatase type 1 regulator-like protein	0.11	0.07	1.274E7	44.7
LmjF.14.0990	ADP/ATP mitochondrial carrier-like protein	0.11	0.08	1.304E7	40.5
LmjF.05.1040	stomatin-like protein	0.10	0.08	1.439E7	39.7
LmjF.27.1870	trypanothione synthetase, putative (TRYS)	0.10	0.08	5.319E7	74.4
LmjF.06.0730	serine-threonine dehydratase, putative	0.10	0.04	2.138E7	37.0
LmjF.31.2600	calreticulin, putative	0.10	0.04	7.346E7	45.1
LmjF.24.0850	triosephosphate isomerase	0.10	0.03	1.348E8	27.2
LmjF.36.3770	basic transcription factor 3a, putative	0.09	0.11	1.918E7	11.7
LmjF.36.5360	ubiquinone biosynthesis methyltransferase, putative	0.08	0.07	1.859E7	32.0
LmjF.20.1690	SNAP protein, putative	0.08	0.08	1.341E7	31.9
LmjF.13.0870	mitochondrial processing peptidase alpha subunit, putative	0.07	0.03	1.133E8	57.7
LmjF.32.0050	protein transport protein Sec13, putative	0.07	0.07	1.342E7	36.3
LmjF.27.0420	ribokinase, putative	0.07	0.05	3.183E7	35.3
LmjF.17.0735	Lysine decarboxylase-like protein	0.07	0.02	4.126E7	35.4

LmjF.28.0690	serine/threonine protein phosphatase catalytic subunit, putative	0.07	0.05	2.196E7	34.4
LmjF.05.0100	phosphoprotein phosphatase, putative	0.06	0.03	1.570E7	71.7
LmjF.01.0650	mitochondrial processing peptide beta subunit, putative	0.06	0.03	1.898E7	55.0
LmjF.11.1050	SEC61-like (pretranslocation process) protein, putative	0.06	0.05	2.677E7	53.9
LmjF.24.0420	ubiquitin carboxyl-terminal hydrolase, putative	0.06	0.01	1.902E7	34.4
LmjF.31.1750	nucleosome assembly protein-like protein	0.06	0.03	6.238E7	45.4
LmjF.10.1110	PAB1-binding protein , putative	0.05	0.04	1.658E7	56.2
LmjF.27.2390	TPR-repeat protein, putative	0.05	0.02	1.034E7	53.6
LmjF.16.0760	transaldolase, putative	0.05	0.01	1.312E8	36.9
LmjF.33.0310	ATP-binding cassette protein subfamily F, member 3, putative (ABCF2)	0.04	0.03	2.054E7	74.5
LmjF.24.2060	transketolase	0.04	0.02	1.273E8	71.6
LmjF.36.6750	prolyl oligopeptidase, putative	0.04	0.03	1.001E7	78.2
LmjF.24.0730	signal recognition particle, putative	0.04	0.03	1.743E7	56.7
LmjF.07.0630	vacuolar-type Ca2 -ATPase, putative	0.03	0.02	3.560E7	122.2
LmjF.22.0650	guide RNA associated protein, GAP2, putative	0.03	0.03	6.214E6	54.6
