# Supplementary material for: The eIF3 complex of Leishmania—subunit composition and mode of recruitment to different cap-binding complexes
Source: Nucleic Acids Res. 2015 Jun 19;43(13):6222–35. doi: 10.1093/nar/gkv564 (PMC4513851; doi:10.1093/nar/gkv564)
Supplement: SUPPLEMENTARY DATA [file supp_gkv564_nar-01173-v-2015-File011.docx]

| **Accession No** | **Protein** | **Relative PAF** |
| --- | --- | --- |
| **eIF3 subunits** |  |  |
| LmjF.17.0010 | eukaryotic translation initiation factor 3a | 1.00 |
| LmjF.17.1290 | eukaryotic translation initiation factor 3b | 0.78 |
| LmjF.36.6980 | eukaryotic translation initiation factor 3c | 0.40 |
| LmjF.30.3040 | eukaryotic translation initiation factor 3d | 0.21 |
| LmjF.28.2310 | eukaryotic translation initiation factor 3e | 0.70 |
| LmjF.25.1610 | eukaryotic translation initiation factor 3f | 0.86 |
| LmjF.34.2700 | eukaryotic translation initiation factor 3g | 0.54 |
| LmjF.07.0640 | eukaryotic translation initiation factor 3h | 0.69 |
| LmjF.36.3880 | eukaryotic translation initiation factor 3i | 0.60 |
| LmjF.32.2180 | eukaryotic translation initiation factor 3k | 0.58 |
| LmjF.36.0250 | eukaryotic translation initiation factor 3l | 0.57 |
| **Other initiation factors** |  |  |
| LmjF.24.1210 | translation factor sui1, putative | 0.70 |
| LmjF.08.0550 | eukaryotic translation initiation factor 2 beta subunit, putative | 0.31 |
| LmjF.09.1070 | eukaryotic translation initiation factor 2 subunit, putative | 0.35 |
| LmjF.03.0980 | eukaryotic initiation factor 2a, putative | 0.42 |
| LmjF.34.0350 | eukaryotic translation initiation factor 5, putative | 0.66 |
| LmjF.16.0140 | eukaryotic translation initiation factor 1A, putative | 0.46 |
| LmjF.01.0770 | eukaryotic initiation factor 4a, putative | 0.45 |
|  |  |  |

**Supplemental Table 3. LC-MS/MS analysis of eIF3 subunits and other initiation factors co-purified with SBP tagged LeishIF3a**. LeishIF3a was tagged at the C-terminus and its associated complex was purified over Streptavidin beads. The purified complex was further analyzed by LC-MS/MS. The calculated PAF values were subjected to background subtraction of the PAF values obtained in a control purification experiment using SBP-tagged luciferase. The relative PAF values give an indication for the relative abundance of the eIF3 subunits and other initiation factors that were present in the pulled down complex.
