# Supplementary material for: The eIF3 complex of Leishmania—subunit composition and mode of recruitment to different cap-binding complexes
Source: Nucleic Acids Res. 2015 Jun 19;43(13):6222–35. doi: 10.1093/nar/gkv564 (PMC4513851; doi:10.1093/nar/gkv564)
Supplement: SUPPLEMENTARY DATA [file supp_gkv564_nar-01173-v-2015-File012.pdf]

Supplemental Table 4. **The complete list of proteins pulled down by tagged LeishIF4G3**.The table shows the proteins that were pulled down with the SBP-tagged LeishIF4G3 and further analyzed by LC-MS/MS. The relative abundance of the proteins is represented by peptide peak areas and relative PAF values. The latter were determined by normalizing the peptide count of each protein to its molecular weight. The relative PAF was calculated for each protein by normalization to the PAF value of the bait protein. Proteins represented by less than two peptides were not included. All values were subjected to background substraction using the peptide count from a mock purification with a cell line expressing the tagged luciferase protein

Accession Number	Protein	Relative PAF	Area	Mol.Wt
**Initiation factors**				
**LmjF.16.1600**	**eukaryotic translation initiation factor 4 gamma, eIF4G3**	**1.00**	**1.010E9**	**71.2**
LmjF.30.0450	eukaryotic translation initiation factor 4e, eIF4E4	0.57	3.826E8	33.8
LmjF.01.0770	eukaryotic initiation factor 4a,	0.47	4.722E8	45.3
**LmjF.17.0010**	**eukaryotic translation initiation factor 3a**	**0.23**	**1.961E7**	**87.6**
**LmjF.17.1290**	**eukaryotic translation initiation factor 3b**	**0.26**	**1.663E7**	**80.7**
**LmjF.36.6980**	**eukaryotic translation initiation factor 3c**	**0.28**	**3.118E7**	**82.0**
**LmjF.30.3040**	**eukaryotic translation initiation factor 3d**	**0.09**	**1.263E7**	**60.6**
**LmjF.28.2310**	**eukaryotic translation initiation factor 3e**	**0.25**	**2.612E7**	**46.4**
**LmjF.25.1610**	**eukaryotic translation initiation factor 3f**	**0.09**	**2.522E7**	**36.7**
**LmjF.34.2700**	**eukaryotic translation initiation factor 3g**	**0.11**	**1.037E7**	**28.8**
**LmjF.07.0640**	**eukaryotic translation initiation factor 3h**	**0.18**	**1.999E7**	**37.9**
**LmjF.36.3880**	**eukaryotic translation initiation factor 3i**	**0.10**	**2.130E7**	**45.4**
**LmjF.32.2180**	**eukaryotic translation initiation factor 3k**	**0.13**	**1.236E7**	**26.3**
**LmjF.36.0250**	**eukaryotic translation initiation factor 3l**	**0.14**	**1.321E7**	**72.6**

LmjF.03.0980	initiation factor 2 alpha subunit	0.15	2.431E7	49.0
LmjF.08.0550	translation initiation factor2 beta subunit	0.20	1.843E7	38.0
LmjF.09.1070	eukaryotic translation initiation factor 2 gamma subunit,	0.26	1.467E7	52.6
LmjF.33.2740	translation initiation factor IF-2,	0.06	1.045E7	92.3
LmjF.34.0350	eukaryotic translation initiation factor 5	0.09	8.056E6	42.9
LmjF.35.5040	polyadenylate-binding protein 1	0.21	9.742E6	62.6
LmjF.35.4130	poly(A)-binding protein 2	0.20	2.853E7	65.3
LmjF.36.0890	eukaryotic translation initiation factor 6 (eIF-6)	0.24	1.299E7	27.1
**Elongation factors**				
LmjF.36.0180	elongation factor 2	0.39	1.769E8	94.1
LmjF.17.0081	elongation factor 1-alpha	0.26	1.198E9	49.1
LmjF.34.0840	translation elongation factor 1-beta	0.15	7.242E7	25.6
LmjF.09.0970	elongation factor-1 gamma	0.14	7.780E7	46.2
LmjF.18.0740	elongation factor Tu,	0.11	1.398E7	51.5
LmjF.36.1430	translation elongation factor 1-beta	0.11	6.068E7	23.3
**Ribosomal proteins**				
LmjF.07.0510	60S ribosomal protein L7a	0.50	2.346E8	29.7
LmjF.35.0420	40S ribosomal protein S3A,	0.45	1.978E8	30.0
LmjF.32.3130	ribosomal protein L3,	0.45	1.027E8	47.5
LmjF.13.1230	40S ribosomal protein S4,	0.44	1.067E8	30.7
LmjF.32.0450	40S ribosomal protein S2	0.38	6.409E7	28.6
LmjF.29.1090	ribosomal protein L1a,	0.33	1.061E8	41.0
LmjF.21.1780	40S ribosomal protein S6,	0.32	6.481E7	28.3
LmjF.36.5010	40S ribosomal protein SA,	0.31	2.029E8	27.5
LmjF.32.3900	60S ribosomal protein L2	0.30	8.569E7	28.3
LmjF.15.0200	60S ribosomal protein L13a	0.28	4.850E7	25.4
LmjF.35.1880	60S ribosomal protein L5	0.27	1.442E8	34.0
LmjF.29.2460	60S ribosomal protein L13	0.23	2.190E8	24.7
LmjF.24.2080	40S ribosomal protein S8,	0.23	2.923E7	24.9
LmjF.04.0750	60S ribosomal protein L10	0.18	3.138E7	24.5

LmjF.20.1650	ribosomal protein S11 homolog	0.16	2.325E6	16.3
LmjF.35.1910	ribosomal protein L15,	0.11	2.742E6	24.0
**tRNA synthetase**				
LmjF.15.1440	glutaminyl-tRNA synthetase	0.11	7.769E6	66.0
LmjF.30.3240	glutamyl-tRNA synthetase	0.10	4.551E6	67.6
LmjF.27.1310	arginyl-tRNA synthetase	0.31	1.609E7	78.2
LmjF.11.0100	seryl-tRNA synthetase	0.23	2.275E7	53.1
LmjF.15.0230	lysyl-tRNA synthetase	0.20	1.205E7	67.0
LmjF.16.1130	tyrosyl or methionyl-tRNA synthetase-like protein	0.20	7.812E6	19.6
LmjF.35.1410	threonyl-tRNA synthetase	0.19	1.225E7	89.4
LmjF.36.3840	glycyl tRNA synthetase	0.15	1.092E7	70.3
LmjF.21.0810	methionyl-tRNA synthetase	0.11	6.022E6	83.8
LmjF.18.1210	prolyl-tRNA synthetase	0.10	1.066E7	81.5
LmjF.34.2340	asparaginyl-tRNA synthetase	0.09	5.290E6	99.4
LmjF.12.0250	cysteinyl-tRNA synthetase	0.09	7.447E6	88.5
**Molecular chaperons**				
LmjF.27.1260	T-complex protein 1, beta subunit	0.42	3.448E7	57.8
LmjF.11.0350	14-3-3 protein	0.38	2.987E7	29.1
LmjF.23.1220	T-complex protein 1, gamma subunit	0.38	1.940E7	60.3
LmjF.13.1660	chaperonin TCP20	0.35	1.381E7	58.9
LmjF.36.3210	14-3-3 protein-like protein	0.35	6.466E7	29.7
LmjF.33.2390	heat shock protein	0.31	1.470E7	72.0
LmjF.21.1090	T-complex protein 1, delta subunit	0.30	1.417E7	59.7
LmjF.30.2550	heat shock 70-related protein 1, mitochondrial precursor	0.27	1.760E8	70.6
LmjF.36.2030	chaperonin HSP60, mitochondrial precursor	0.27	1.926E8	59.3
LmjF.32.1000	chaperonin containing t-complex protein	0.26	1.169E7	59.2
LmjF.32.3270	chaperonin alpha subunit	0.24	3.691E7	59.1
LmjF.08.1110	stress-induced protein sti1	0.23	1.329E7	62.1
LmjF.28.1200	glucose-regulated protein 78	0.20	1.300E8	71.9
LmjF.26.1240	heat shock protein 70-related protein	0.18	5.183E7	70.5

LmjF.26.0620	10 kDa heat shock protein	0.18	7.209E6	10.7
LmjF.18.1370	heat shock protein	0.18	5.246E7	91.7
LmjF.36.6910	T-complex protein 1, theta subunit,	0.18	3.060E7	58.1
LmjF.33.0355	heat shock protein 83-1	0.18	5.036E8	80.5
LmjF.36.6940	protein disulfide isomerase	0.16	1.222E8	52.3
LmjF.36.0070	stress-inducible protein STI1 homolog	0.16	1.096E7	29.0
LmjF.04.0770	nascent polypeptide associated complex subunit- like protein, copy 1	0.14	4.876E6	18.2
LmjF.35.3860	T-complex protein 1, eta subunit	0.12	2.438E7	61.7
**RNA binding proteins**				
LmjF.32.0750	RNA binding protein	0.28	1.711E7	25.2
LmjF.35.2200	RNA-binding protein	0.23	1.013E7	30.2
LmjF.04.1170	RNA-binding protein	0.19	1.106E7	34.5
LmjF.23.0760	mitochondrial RNA binding protein	0.16	8.010E6	39.7
LmjF.09.1120	mitochondrial RNA binding protein 2,MRP2, gBP25	0.14	4.542E6	26.8
LmjF.07.0990	nucleolar RNA-binding protein	0.12	1.110E7	37.5
LmjF.21.0540	la RNA binding protein	0.10	1.776E8	37.2
**RNA/DNA helicases**				
LmjF.21.1552	RNA helicase	0.47	3.982E7	49.5
LmjF.32.0400	ATP-dependent RNA helicase	0.42	3.234E7	67.0
LmjF.34.2610	ATP-dependent DNA helicase, ,ruvb DNA helicase-like protein	0.36	2.086E7	53.6
LmjF.34.3500	ruvb-like 1 DNA helicase	0.33	1.295E7	50.3
LmjF.35.3100	ATP-dependent RNA helicase	0.14	1.500E7	100.2
LmjF.07.0340	ATP-dependent DEAD/H RNA helicase	0.14	1.077E7	64.0
**Cytoskelton**				
LmjF.04.1230	actin	0.34	1.109E8	42
LmjF.13.0390	alpha tubulin	0.33	9.866E7	49.7
LmjF.33.0808	beta tubulin	0.23	7.598E7	49.7

**Protein degradation machinery**				
LmjF.36.1600	proteasome alpha 1 subunit	0.24	1.945E7	29.6
LmjF.21.1830	proteasome alpha 5 subunit, ,20S proteasome subunit alpha 5	0.21	1.704E7	26.8
LmjF.35.1380	mitochondrial processing peptidase, beta subunit	0.20	2.510E7	54.5
LmjF.02.0370	proteasome regulatory non-ATPase subunit 6	0.16	1.008E7	58.8
LmjF.11.0240	proteasome alpha 7 subunit	0.14	8.929E6	27.8
LmjF.34.4370	20s proteasome beta 7 subunit	0.13	6.880E6	24.7
LmjF.33.1610	peptidase M20/M25/M40	0.13	2.253E7	51.5
LmjF.26.1570	thimet oligopeptidase, ,metallo-peptidase, Clan MA(E), Family M3	0.12	1.520E7	77.1
LmjF.36.4360	proteasome regulatory ATPase subunit	0.11	1.447E7	45.6
LmjF.27.1460	proteasome regulatory non-ATP-ase subunit 3	0.10	6.522E6	37.9
LmjF.27.2660	peptidyl dipeptidase, ,metallo-peptidase, Clan MA(E), Family M3	0.10	8.814E6	76.5
LmjF.19.1120	proteasome regulatory non-ATP-ase subunit	0.10	9.224E6	46.5
LmjF.21.0760	proteasome regulatory non-ATP-ase subunit 5, ,19S proteasome regulatory subunit 4	0.10	6.585E6	54.2
**Signal transduction**				
LmjF.28.2750	activated protein kinase c receptor (LACK)	0.53	8.252E7	34.4
LmjF.30.2740	small glutamine-rich tetratricopeptide repeat protein	0.24	1.703E7	45.7
LmjF.19.0150	protein kinase, ,mitogen-activated protein kinase,	0.21	1.043E7	30.9
LmjF.25.0750	protein phosphatase	0.17	1.003E7	44.9
LmjF.27.2330	GTP binding protein,	0.13	3.171E7	44.0
LmjF.13.0160	protein kinase A regulatory subunit	0.13	1.039E7	56.2
LmjF.25.1420	GTP-binding protein	0.12	4.127E6	24.2
LmjF.10.0490	mitogen-activated protein kinase 3, ,map kinase 3	0.09	9.120E6	43.8
LmjF.10.0200	mitogen-activated protein kinase, ,map kinase-like protein	0.08	6.950E6	46.3
LmjF.05.0280	protein tyrosine phosphatase	0.08	3.021E6	25.2
LmjF.21.1080	cell division protein kinase 2,cdc2-related kinase	0.08	3.475E6	34.4
**Metabolic enzymes**				
LmjF.14.1160	enolase	0.45	1.558E8	46.1

LmjF.25.2130	succinyl-CoA synthetase alpha subunit	0.40	5.961E7	30.9
LmjF.10.0290	isocitrate dehydrogenase [NADP], mitochondrial precursor,	0.39	4.152E7	48.5
LmjF.01.0050	carboxylase,	0.39	1.458E8	73.7
LmjF.36.2950	succinyl-CoA ligase [GDP-forming] beta-chain	0.32	7.184E7	44.2
LmjF.34.3670	vacuolar ATP synthase catalytic subunit A	0.31	1.275E7	67.7
LmjF.36.3910	S-adenosylhomocysteine hydrolase	0.30	5.565E7	47.8
LmjF.21.0240	hexokinase,	0.30	5.857E7	51.7
LmjF.32.3310	dihydrolipoamide dehydrogenase	0.28	4.352E7	50.5
LmjF.28.2420	2-oxoglutarate dehydrogenase, E2 component, dihydrolipoamide succinyltransfera	0.26	6.312E7	41.7
LmjF.24.0770	malic enzyme,	0.25	1.085E7	63.4
LmjF.36.1260	fructose-1,6-bisphosphate aldolase	0.25	6.374E7	40.8
LmjF.35.0820	aspartate aminotransferase,	0.25	2.360E7	46.0
LmjF.33.2540	carboxypeptidase, ,metallo-peptidase, Clan MA(E), Family M32	0.25	1.916E7	57.0
LmjF.30.2970	glyceraldehyde 3-phosphate dehydrogenase, glycosomal	0.25	9.344E7	39.1
LmjF.27.1805	glycosomal phosphoenolpyruvate carboxykinase,	0.23	3.882E7	58.2
LmjF.29.0760	lipophosphoglycan biosynthetic protein, ,heat shock protein 90, ,glucose regulated	0.22	1.132E7	86.6
LmjF.31.2150	prostaglandin f2-alpha synthase/D-arabinose dehydrogenase	0.22	2.034E7	31.8
LmjF.35.3230	cystathione gamma lyase,	0.22	9.869E7	44.5
LmjF.23.1480	alanine racemase	0.22	1.131E7	27.1
LmjF.35.1480	arginase	0.21	3.309E7	36.1
LmjF.19.0160	aminopeptidase, ,metallo-peptidase, Clan MG, Family M24	0.21	4.433E7	42.5
LmjF.28.2430	vacuolar ATP synthase subunit b,	0.21	2.783E7	55.5
LmjF.36.1370	Transitional endoplasmic reticulum ATPase	0.21	1.840E7	86.8
LmjF.35.3700	Gim5A protein, ,glycosomal membrane protein	0.21	1.652E7	24.9
LmjF.36.3100	ATP synthase,	0.21	1.043E7	25.1
LmjF.28.0490	propionyl-coa carboxylase beta chain,	0.21	8.698E7	56.7
LmjF.31.1630	3-ketoacyl-CoA thiolase-like protein,	0.20	7.152E6	38.1
LmjF.19.0200	ADP,ATP carrier protein 1, mitochondrial precursor, ,ADP/ATP translocase 1,	0.20	1.277E8	35.1
LmjF.23.0540	acetyl-CoA synthetase,	0.20	4.059E7	78.5
LmjF.16.0550	orotidine-5-phosphate decarboxylase/orotate phosphoribosyltransferase	0.20	1.384E7	49.5
LmjF.19.0710	glycosomal malate dehydrogenase	0.19	1.846E7	33.6
LmjF.25.2010	2,4-dihydroxyhept-2-ene-1,7-dioic acid aldolase	0.19	2.009E7	30.3
LmjF.35.0030	pyruvate kinase	0.19	2.074E7	54.2
LmjF.25.1170	ATPase beta subunit	0.18	8.585E7	56.3
LmjF.18.0510	aconitase	0.18	2.644E7	97.4

LmjF.35.1010	casein kinase	0.18	1.242E7	39.7
LmjF.28.2510	acyl-CoA dehydrogenase	0.18	1.354E7	68.8
LmjF.12.0530	glucose-6-phosphate isomerase	0.17	9.874E6	67.1
LmjF.31.2970	acetyl-CoA carboxylase	0.17	2.379E8	241.0
LmjF.23.0710	acetyl-CoA synthetase	0.17	3.735E7	77.4
LmjF.36.3590	cysteine synthase	0.16	2.716E7	35.4
LmjF.29.1570	glutamamyl carboxypeptidase, ,metallo-peptidase, Clan MH, Family M18	0.16	1.532E7	43.8
LmjF.36.1140	short chain 3-hydroxyacyl-CoA dehydrogenase	0.16	1.236E7	33.0
LmjF.29.1310	carnitine/choline acetyltransferase	0.15	7.438E6	68.6
LmjF.36.6650	2,3-bisphosphoglycerate-independent phosphoglycerate mutase	0.15	1.967E7	60.7
LmjF.27.0930	isovaleryl-coA dehydrogenase	0.15	2.960E7	44.7
LmjF.13.0090	carboxypeptidase, ,metallo-peptidase, Clan MA(E), family 32	0.15	2.220E7	57.0
LmjF.36.4170	oxidoreductase,	0.14	1.033E7	36.1
LmjF.31.1070	biotin/lipoate protein ligase-like protein	0.14	1.717E7	28.3
LmjF.03.0200	delta-1-pyrroline-5-carboxylate dehydrogenase	0.14	1.192E7	61.9
LmjF.33.2340	succinyl-coA:3-ketoacid-coenzyme A transferase, mitochondrial precursor	0.13	1.280E7	52.5
LmjF.05.0510	ATPase alpha subunit	0.13	9.294E7	62.5
LmjF.34.0110	adenylate kinase	0.13	1.363E7	24.4
LmjF.35.4430	mitochondrial phosphate transporter	0.13	1.155E7	34.6
LmjF.27.1870	trypanothione synthetase	0.13	8.338E6	74.4
LmjF.31.2600	calreticulin	0.13	3.749E7	45.1
LmjF.29.1960	fumarate hydratase	0.12	7.493E6	62.6
LmjF.23.0110	GDP-mannose pyrophosphorylase	0.12	2.164E7	41.7
LmjF.26.0320	C-1-tetrahydrofolate synthase, cytoplasmic	0.12	1.081E7	31.7
LmjF.31.2250	3,2-trans-enoyl-CoA isomerase, mitochondrial precursor	0.12	1.689E7	42.8
LmjF.20.1560	aminoacylase, ,N-acyl-L-amino acid amidohydrolase	0.12	1.209E7	42.8
LmjF.27.2020	D-lactate dehydrogenase-like protein	0.12	1.619E7	53.7
LmjF.05.0960	dipeptidyl-peptidase III, ,metallo-peptidase, Clan M-, Family M49	0.12	9.498E6	75.9
LmjF.15.1010	glutamate dehydrogenase	0.12	1.833E7	115.0
LmjF.19.1560	inosine-5'-monophosphate dehydrogenase	0.12	1.264E7	55.5
LmjF.20.0110	phosphoglycerate kinase B, cytosolic	0.12	2.985E7	44.9
LmjF.35.1230	short chain dehydrogenase,	0.11	1.005E7	28.1
LmjF.16.0530	dihydroorotate dehydrogenase	0.11	8.017E6	34.6
LmjF.06.0860	dihydrofolate reductase-thymidylate synthase	0.11	5.833E6	58.7
LmjF.17.0725	guanosine monophosphate reductase	0.11	9.729E6	58.8

LmjF.31.1220	vacuolar-type proton translocating pyrophosphatase 1	0.11	1.842E7	83.5
LmjF.18.0670	citrate synthase,	0.10	1.917E7	50.4
LmjF.22.0770	NADH-cytochrome b5 reductase	0.10	4.714E6	31.8
LmjF.30.2900	aldehyde dehydrogenase	0.10	4.814E6	63.9
LmjF.14.0130	inosine-guanine nucleoside hydrolase	0.10	7.637E6	39.0
LmjF.16.0950	sucrose-phosphate synthase-like protein	0.10	8.569E6	52.4
LmjF.12.0670	cytochrome c oxidase subunit IV	0.10	1.266E7	39.4
LmjF.34.0140	malate dehydrogenase	0.10	8.714E7	33.3
LmjF.14.0990	ADP/ATP mitochondrial carrier-like protein	0.10	4.013E6	40.5
LmjF.21.1340	ATP synthase,	0.10	1.509E7	54.0
LmjF.24.0850	triosephosphate isomerase	0.10	9.966E6	27.2
LmjF.30.1250	Pyridoxal kinase	0.10	5.928E6	33.2
**Hypothetical protein**				
LmjF.34.2580	hypothetical protein, conserved	0.26	1.270E7	22.6
LmjF.27.1300	hypothetical protein, conserved	0.21	1.425E7	59.9
LmjF.09.1010	hypothetical protein, conserved	0.19	1.952E7	67.2
LmjF.08.1100	hypothetical protein, conserved	0.17	1.502E7	42.0
LmjF.24.2110	hypothetical protein, conserved	0.16	2.309E7	55.2
LmjF.32.3210	hypothetical protein, conserved	0.16	1.130E6	24.2
LmjF.25.2020	hypothetical protein, conserved	0.16	4.732E6	32.8
LmjF.28.2170	hypothetical protein, conserved	0.15	3.176E7	71.2
LmjF.22.0300	hypothetical protein, conserved	0.15	6.706E6	60.6
LmjF.29.1240	hypothetical protein, unknown function	0.15	8.144E6	78.8
LmjF.33.1590	hypothetical protein, conserved	0.14	9.274E6	36.1
LmjF.23.1410	hypothetical protein, conserved	0.14	7.158E6	27.4
LmjF.14.0450	hypothetical protein, conserved	0.14	1.042E7	36.9
LmjF.35.4470	hypothetical protein, conserved	0.12	1.607E7	21.5
LmjF.14.0190	hypothetical protein, conserved	0.12	4.987E6	22.3
LmjF.21.0430	hypothetical protein, conserved	0.12	2.865E6	44.7
LmjF.30.3430	hypothetical protein, conserved	0.11	9.907E6	90.2
LmjF.15.0040	hypothetical protein, conserved	0.10	3.482E6	37.7
LmjF.26.2000	hypothetical protein, conserved	0.10	7.886E6	38.9

LmjF.35.0190	hypothetical protein, conserved	0.10	7.256E6	27.2
LmjF.35.0900	hypothetical protein, conserved	0.09	2.611E6	27.9
LmjF.16.0520	hypothetical protein, conserved	0.09	3.429E6	36.9
LmjF.34.0010	hypothetical protein, conserved	0.08	5.173E6	33.6
LmjF.32.0950	hypothetical protein, conserved	0.08	6.004E6	102.2

Miscellaneous

LmjF.15.1450	proliferative cell nuclear antigen (PCNA)	0.20	1.735E7	32.4
LmjF.35.0070	prohibitin,	0.16	1.153E7	32.3
LmjF.32.1730	coatomer epsilon subunit	0.14	1.013E7	35.0
LmjF.30.3380	PAS-domain containing phosphoglycerate kinase	0.13	6.219E6	57.5
LmjF.24.1510	IgE-dependent histamine-releasing factor	0.13	1.403E6	19.4
LmjF.19.0440	nucleosome assembly protein	0.13	1.845E7	39.7
LmjF.31.1750	nucleosome assembly protein-like protein	0.11	7.796E6	45.4
LmjF.30.1120	importin alpha	0.10	1.050E7	58.1
