# Supplementary material for: The eIF3 complex of Leishmania—subunit composition and mode of recruitment to different cap-binding complexes
Source: Nucleic Acids Res. 2015 Jun 19;43(13):6222–35. doi: 10.1093/nar/gkv564 (PMC4513851; doi:10.1093/nar/gkv564)
Supplement: SUPPLEMENTARY DATA [file supp_gkv564_nar-01173-v-2015-File013.pdf]

A

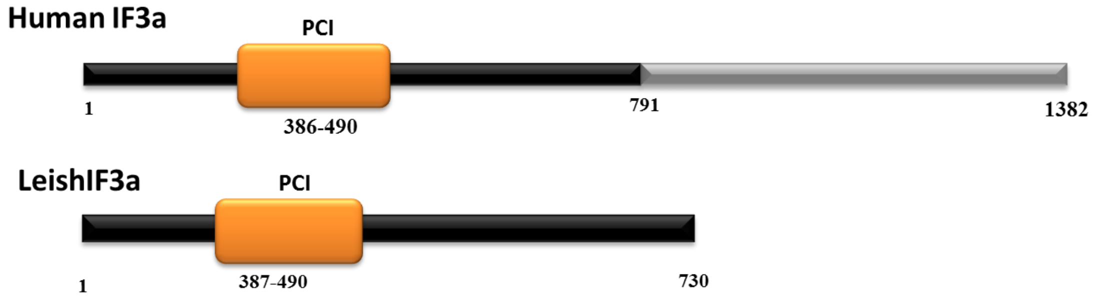

B

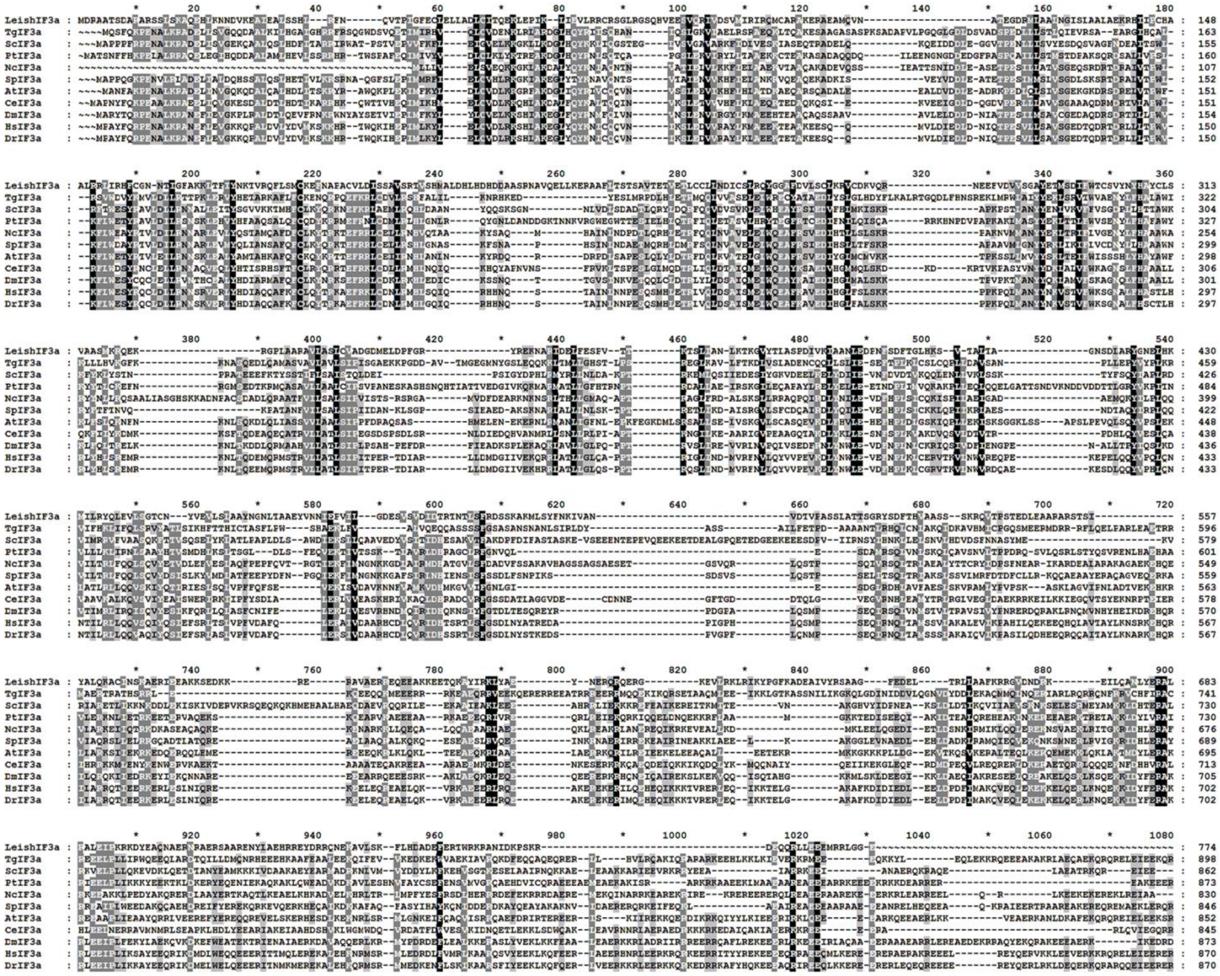

Supplemental Figure 1. A. Schematic representation of the *Leishmania* and human eIF3a proteins. The orange boxes marks the PCI domains of eIF3a and the position of amino acids are indicated. The C-terminal extension of the human IF3a which is not conserved in *Leishmania* is represented by a grey bar. **B**. Multiple sequence alignment of LeishIF3a (LmMx.17.0010) with its orthologs from different organisms: *Homo sapiens* (HsIF3a, NP_003741.1), *Toxoplasma gondii* (TgIF3a, TGME49_201680), *Phaeodactylum tricornutum* (PtIF3a, XP_002180731), *Neurospora crassa* (NcIF3a, XP_956151.2), *Schizosaccharomyces pombe* (SpIF3a, NP_596379.1), *Caenorhabditis elegans* (CeIF3a, NP_498698.1), *Arabidopsis thaliana* (AtIF3a, NP_192881.1), *Drosophila melanogaster* (DmIF3a, NP_649470.2), *Danio rerio* (DrIF3a, NP_956114.2). The non-conserved C-terminal region of the human eIF3 and eIF3 from other eukaryotes was not included in the alignment.

A

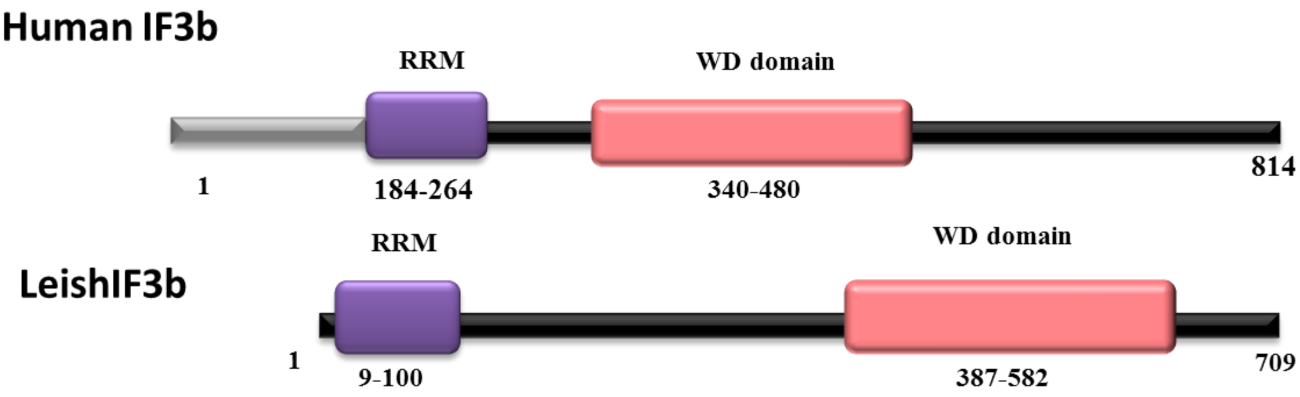
Human IF3b
RRM
WD domain
1
184-264
340-480
814
LeishIF3b
RRM
WD domain
1
9-100
387-582
709

B

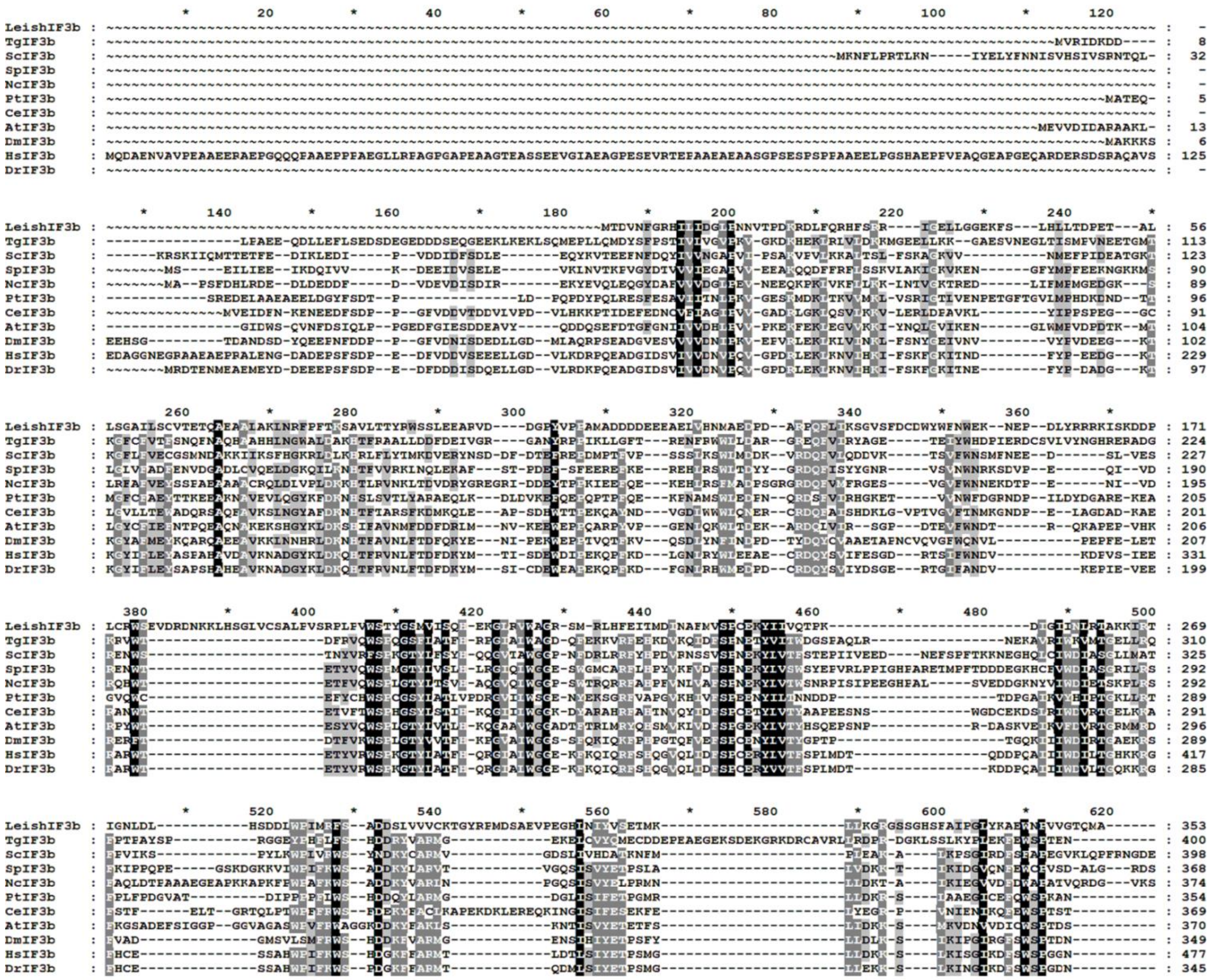

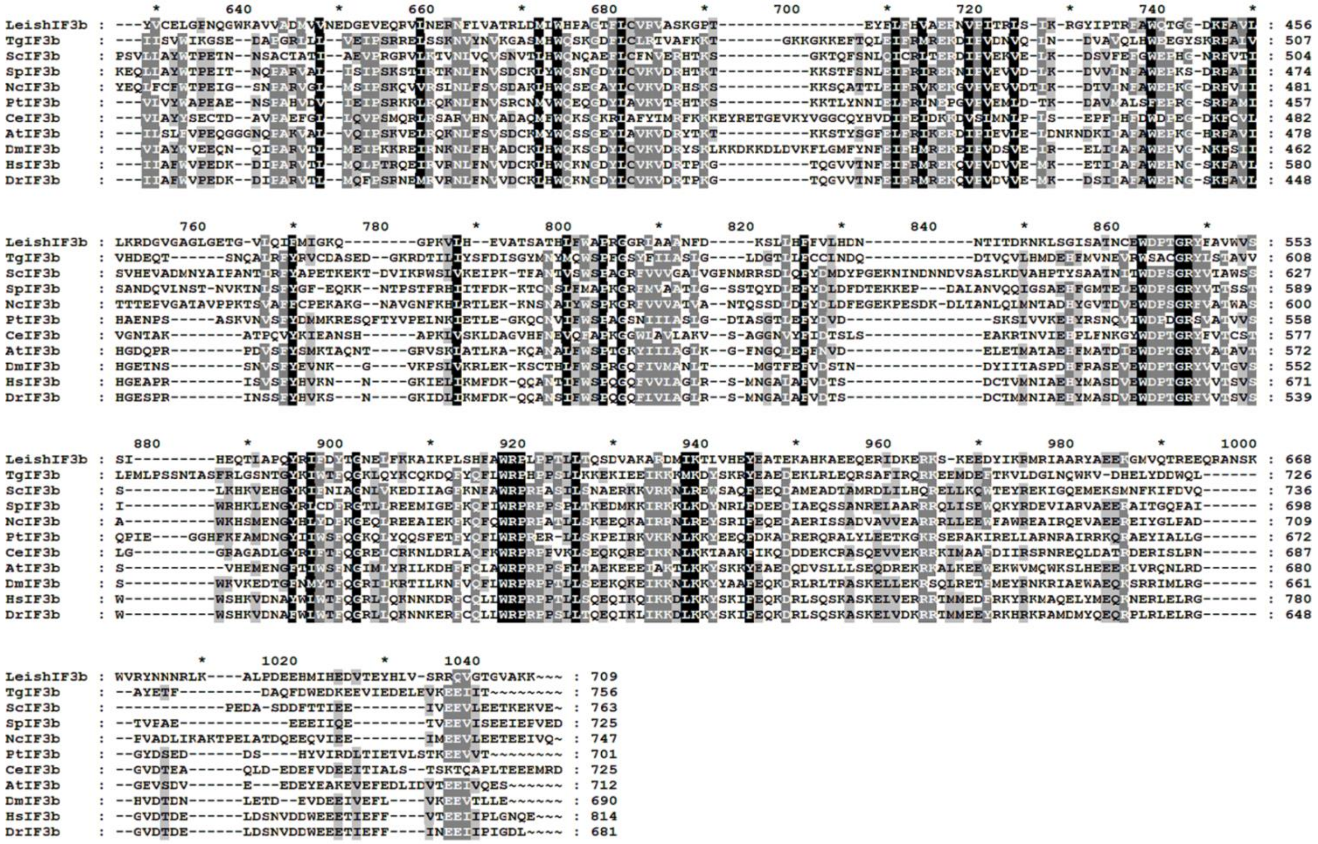

Supplemental Figure 2. A. Schematic representation of the *Leishmania* and human IF3b proteins. The RRM and WD domains are marked by purple and pink boxes, respectively. **B**. Multiple sequence alignment of LeishIF3b with its orthologs from different organisms: *Leishmania mexicana* (LeishIF3b, LmxM17. 1290), *Saccharomyces cerevisiae* (ScIF3b, NP_015006.3), *Toxoplasma gondii* (TgIF3b, XP_002370073.1), *Phaeodactylum tricornutum* (PtIF3b, XP_002179558.1), *Neurospora crassa* (NcIF3b, XP_959527.1), *Schizosaccharomyces pombe* (SpIF3b, NP_594528.1), *Caenorhabditis elegans* (CeIF3b, NP_001022469.2), *Arabidopsis thaliana* (AtIF3b, NP_568498.1), *Drosophila melanogaster* (DmIF3b, NP_725691.1), *Danio rerio* (DrIF3b, NP_001277022.1), *Homo sapiens* (HsIF3b, NP_001032360.1)

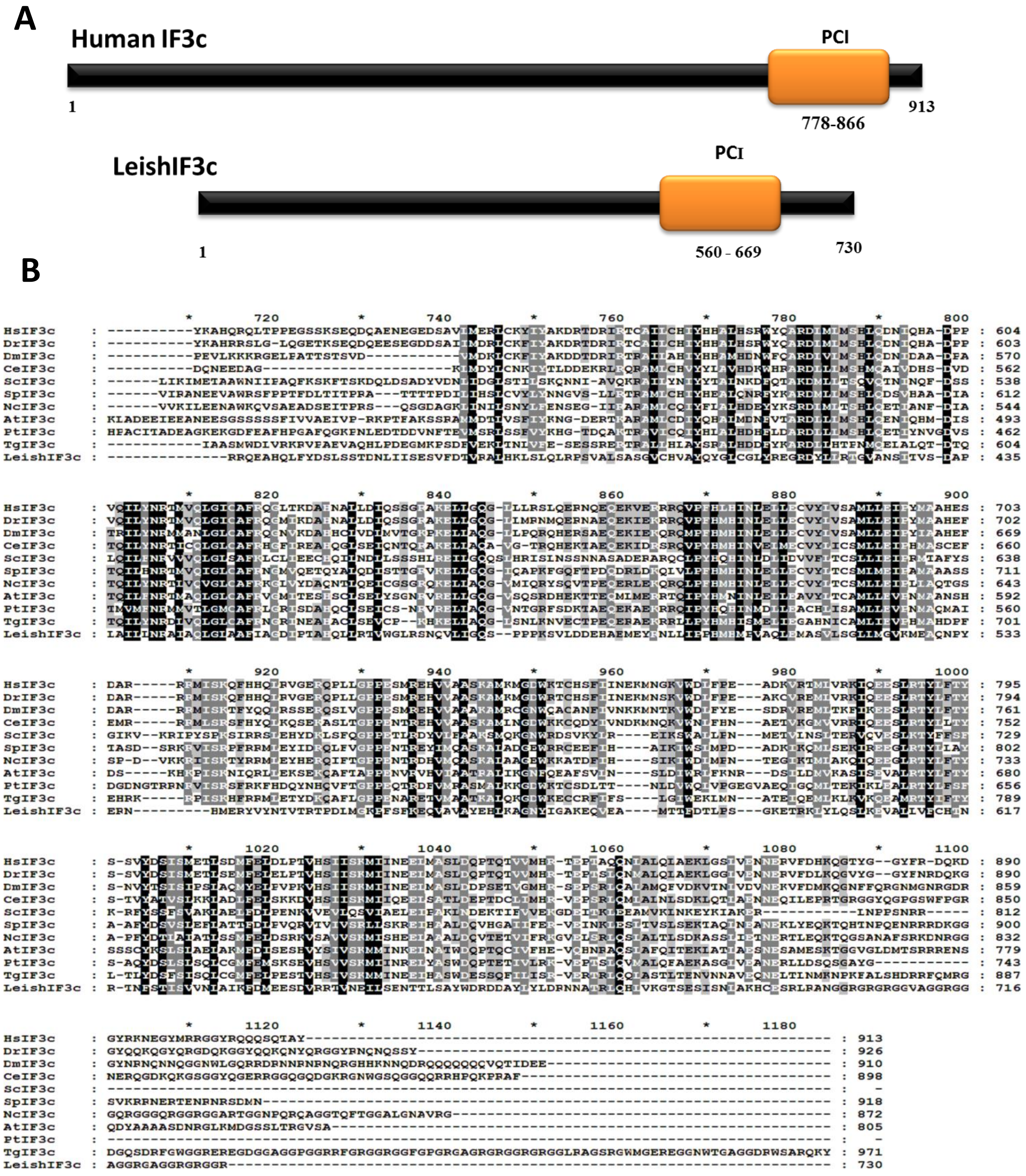

Supplemental Figure 3. A. Schematic representation of the *Leishmania* and human eIF3c proteins. The PCI domain is marked by an orange box. **B**. Multiple sequence alignment of LeishIF3c with its orthologs from different organisms: *Leishmania mexicana* (LmxM36.6980), *Saccharomyces cerevisiae* (ScIF3c, NP_014040.1), *Toxoplasma gondii* (TgIF3c, XP_002370231.1), *Phaeodactylum tricornutum* (PtIF3c, XP_002183081.1), *Neurospora crassa* (NcIF3c, XP_962952.1), *Schizosaccharomyces pombe* (SpIF3c, NP_593828.2), *Caenorhabditis elegans* (CeIF3c, NP_492638.1), *Arabidopsis thaliana* (AtIF3c, NP_188926.4), *Drosophila melanogaster* (DmIF3c, NP_611242.1), *Danio rerio* (DrIF3c, NP_998628.1), *Homo sapiens* (HsIF3c, NP_003743.1)

A

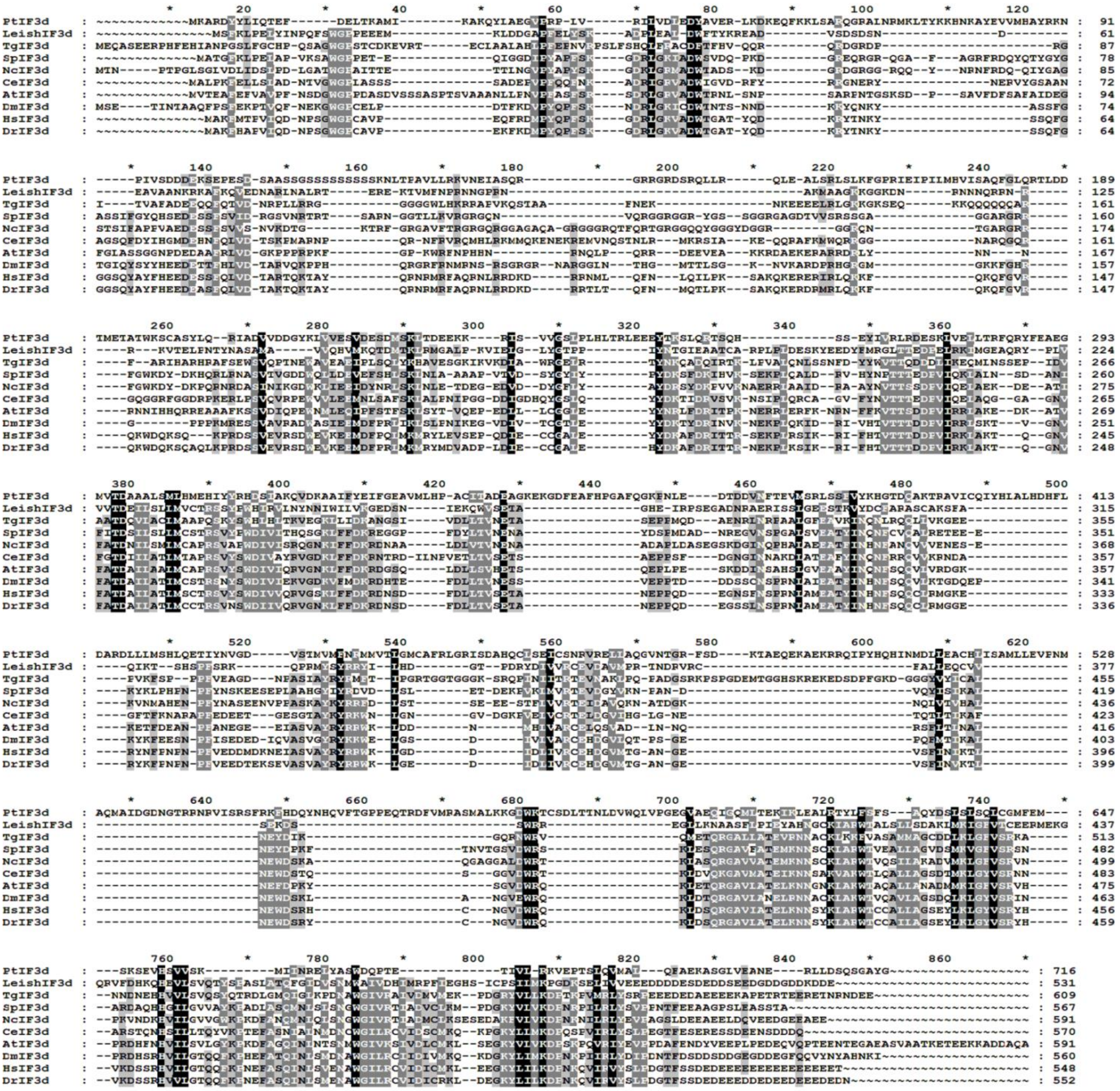

Supplemental Figure 4. Specific domains were difficult to identify. **A.** Multiple sequence alignment of LeishIF3d with its orthologs from different organisms: *Leishmania mexicana* (LmxM29.3040), , *Toxoplasma gondii* (TgIF3d, XP_002372060.1), *Phaeodactylum tricornutum* (PtIF3d, XP_002183081.1), *Neurospora crassa* (NcIF3d, XP_958873.1), *Schizosaccharomyces pombe* (SpIF3d, NP_594625.1), *Caenorhabditis elegans* (CeIF3d, NP_498984.1), *Arabidopsis thaliana* (AtIF3d, NP_193830.1), *Drosophila melanogaster* (DmIF3d, NP_524463.2), *Danio rerio* (DrIF3d, NP_956310.1), *Homo sapiens* (HsIF3d, NP_003744.1).

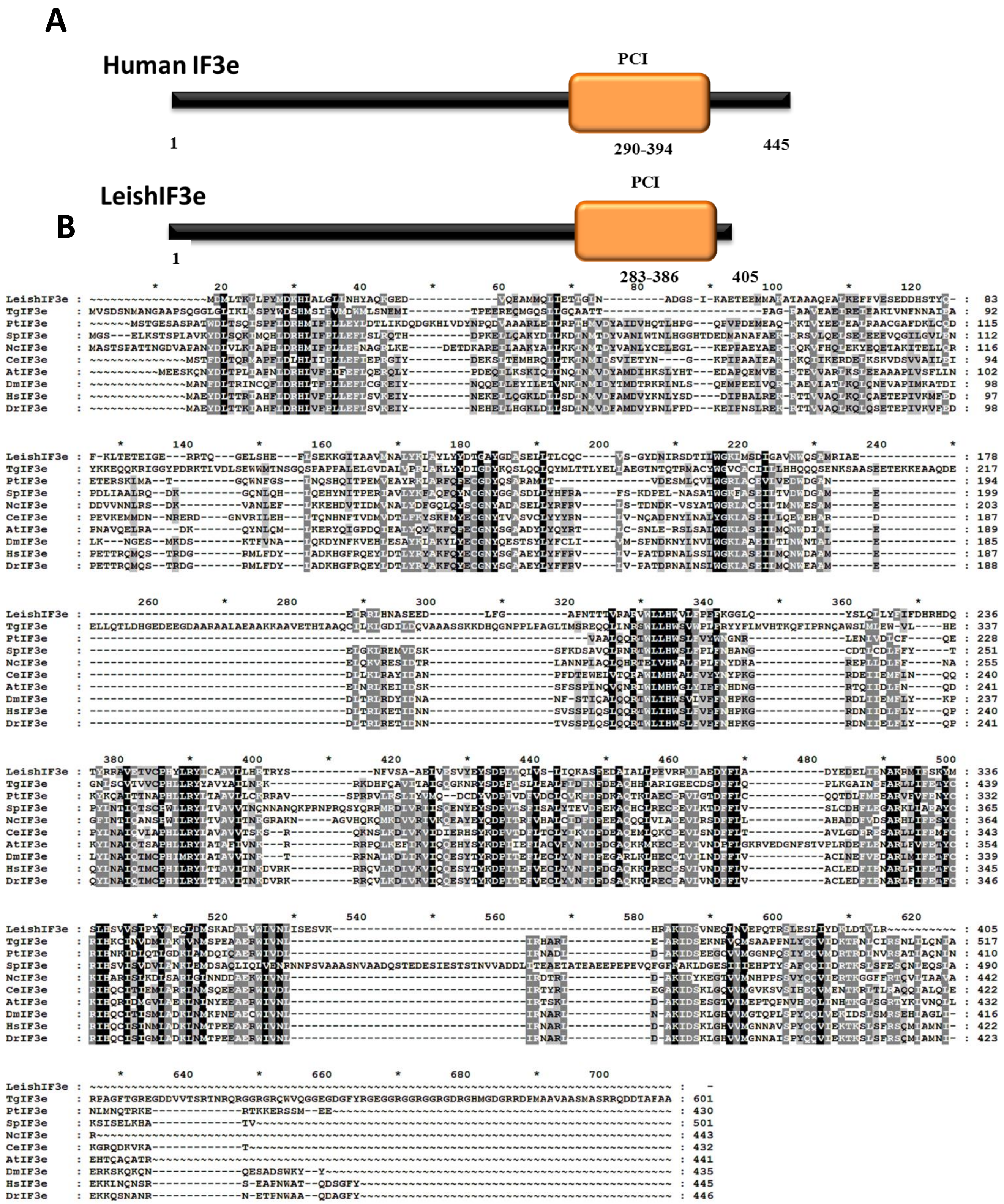

Supplemental Figure 5. A. Schematic representation of the *Leishmania* and human eIF3e proteins. The orange box marks a PCI domain. **B.** Multiple sequence alignment of LeishIF3e with its orthologs from different organisms: *Leishmania mexicana* (LmxM28.2310), *Toxoplasma gondii* (TgIF3e, XP_002365953.1), *Phaeodactylum tricornutum* (PtIF3e, XP_002181668.1), *Neurospora crassa* (NcIF3e, XP_959880.1), *Schizosaccharomyces pombe* (SpIF3e, NP_595367.1), *Caenorhabditis elegans* (CeIF3e, NP_492785.1), *Arabidopsis thaliana* (AtIF3e, NP_567047.1), *Drosophila melanogaster* (DmIF3e, NP_477385.1), *Danio rerio* (DrIF3e, NP_957133.1), *Homo sapiens* (HsIF3e, NP_001559.1)

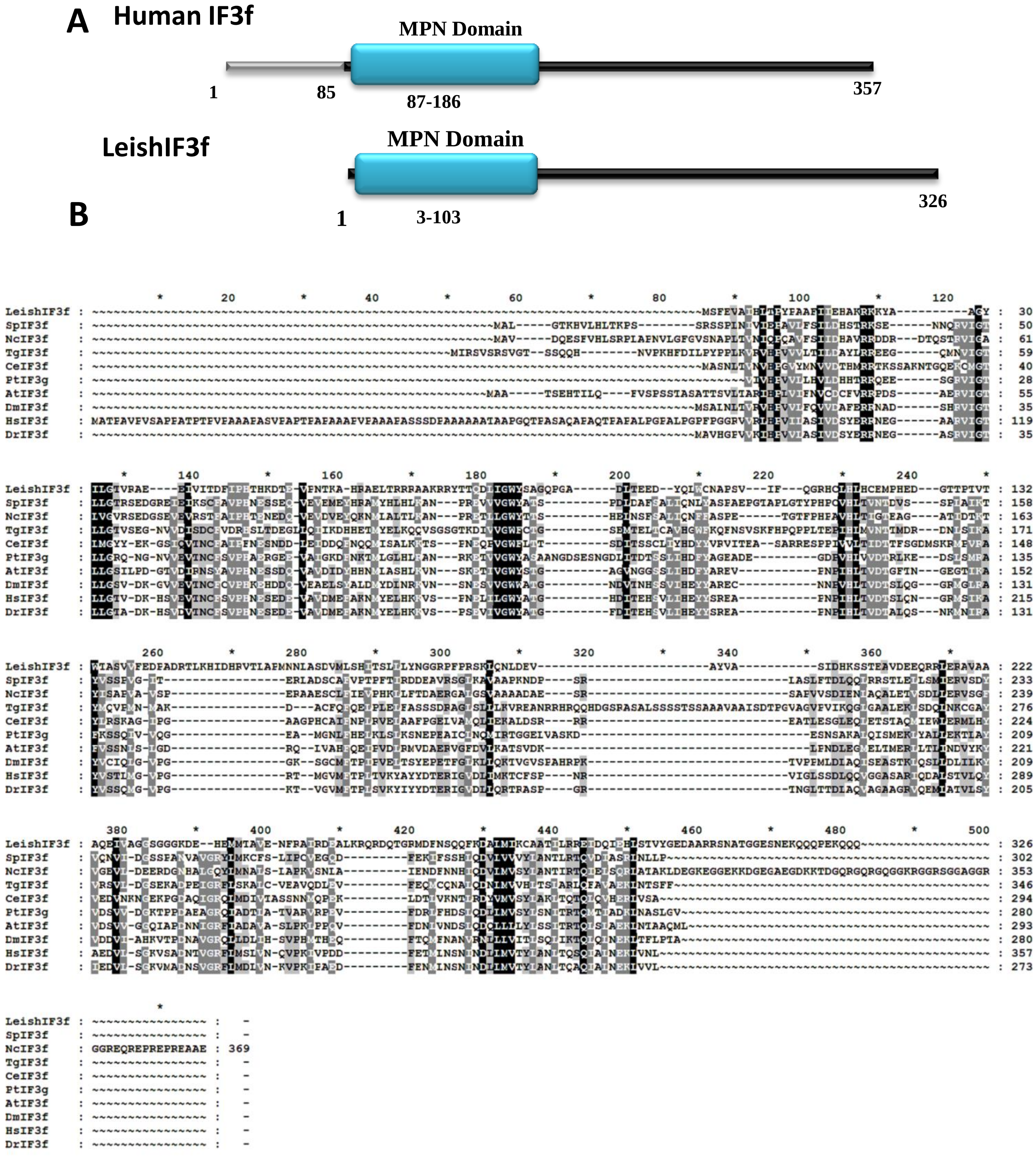

Supplemental Figure 6. A. Schematic representation of the *Leishmania* and human eIF3f proteins. The blue box marks the MPN domain. The *Leishmania* protein lacks the region located upstream of the MPN domain, marked in grey in the human eIF3f. **B.** Multiple sequence alignment of LeishIF3f with its orthologs from different organisms. *Leishmania mexicana* (LmxM25.1610), *Toxoplasma gondii* (TgIF3f, XP_002366441), *Phaeodactylum tricornutum* (PtIF3f, EEC49441.1), *Neurospora crassa* (NcIF3f, XP_961633.1), *Schizosaccharomyces pombe* (SpIF3f, NP_596298.1), *Caenorhabditis elegans* (CeIF3f, NP_495988.1), *Arabidopsis thaliana* (AtIF3f, NP_181528.1), *Drosophila melanogaster* (DmIF3f, NP_649489.1), *Danio rerio* (DrIF3f, NP_001186938.1), *Homo sapiens* (HsIF3f, NP_0037).

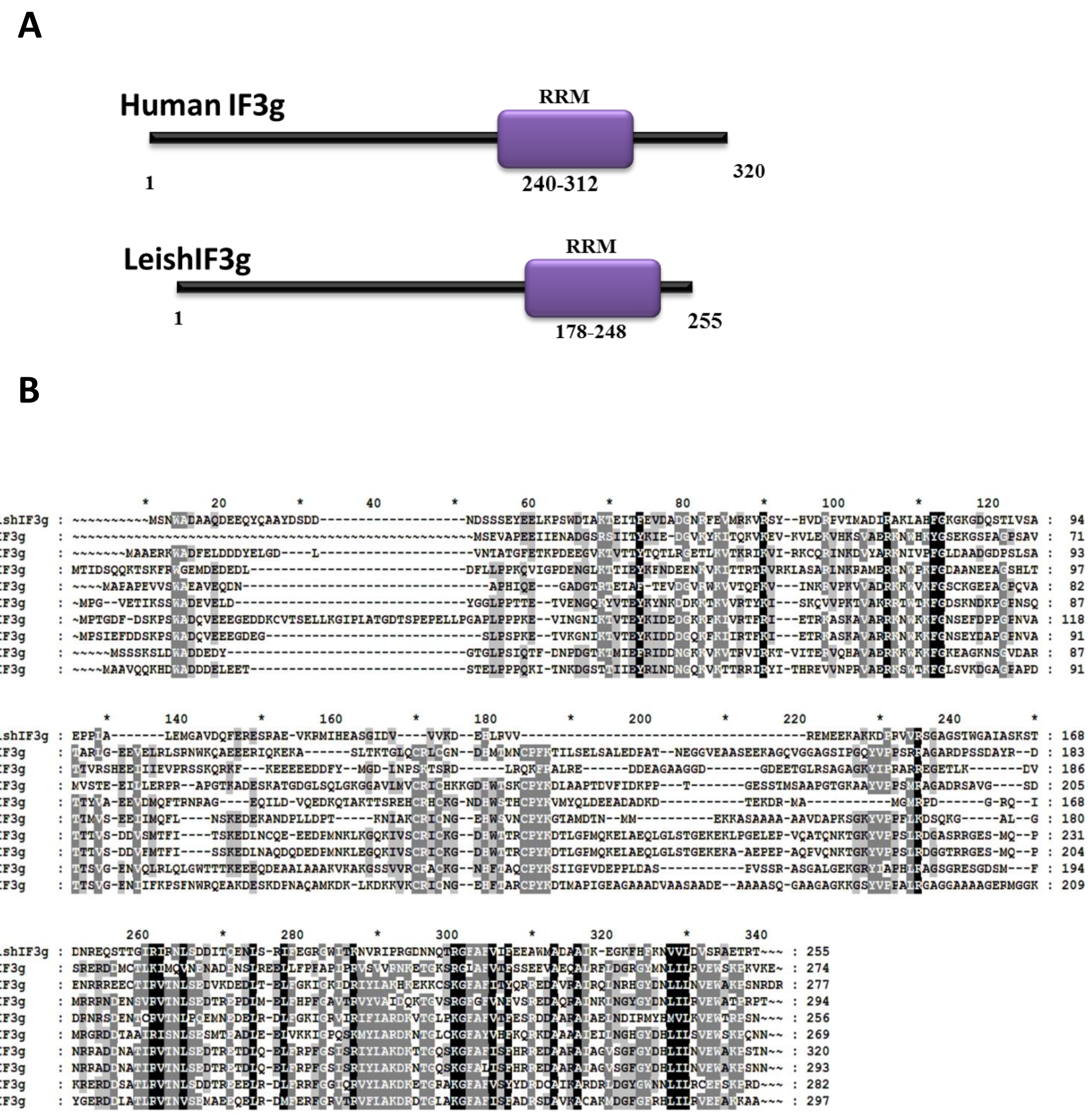

Supplemental Figure 7. A. Schematic representation of h of the *Leishmania* and human eIF3g proteins. The purple box marks the RRM domain. **B.** Multiple sequence alignment of LeishIF3g with its orthologs from different organisms: *Leishmania mexicana* (LmxM33.2700), *Toxoplasma gondii* (TgIF3g, XP_002370236.1), *Neurospora crassa* (NcIF3g, XP_962716.2), *Schizosaccharomyces pombe* (SpIF3g, NP_595727.1), *Caenorhabditis elegans* (CeIF3g, NP_495778.1), *Arabidopsis thaliana* (AtIF3g, NP_187747.1), *Drosophila melanogaster* (DmIF3g, NP_570011.1), *Danio rerio* (DrIF3g, NP_957293.1), *Homo sapiens* (HsIF3g, NP_003746.2) *Saccharomyces cerevisiae* (ScIF3g, NP_010717.1)

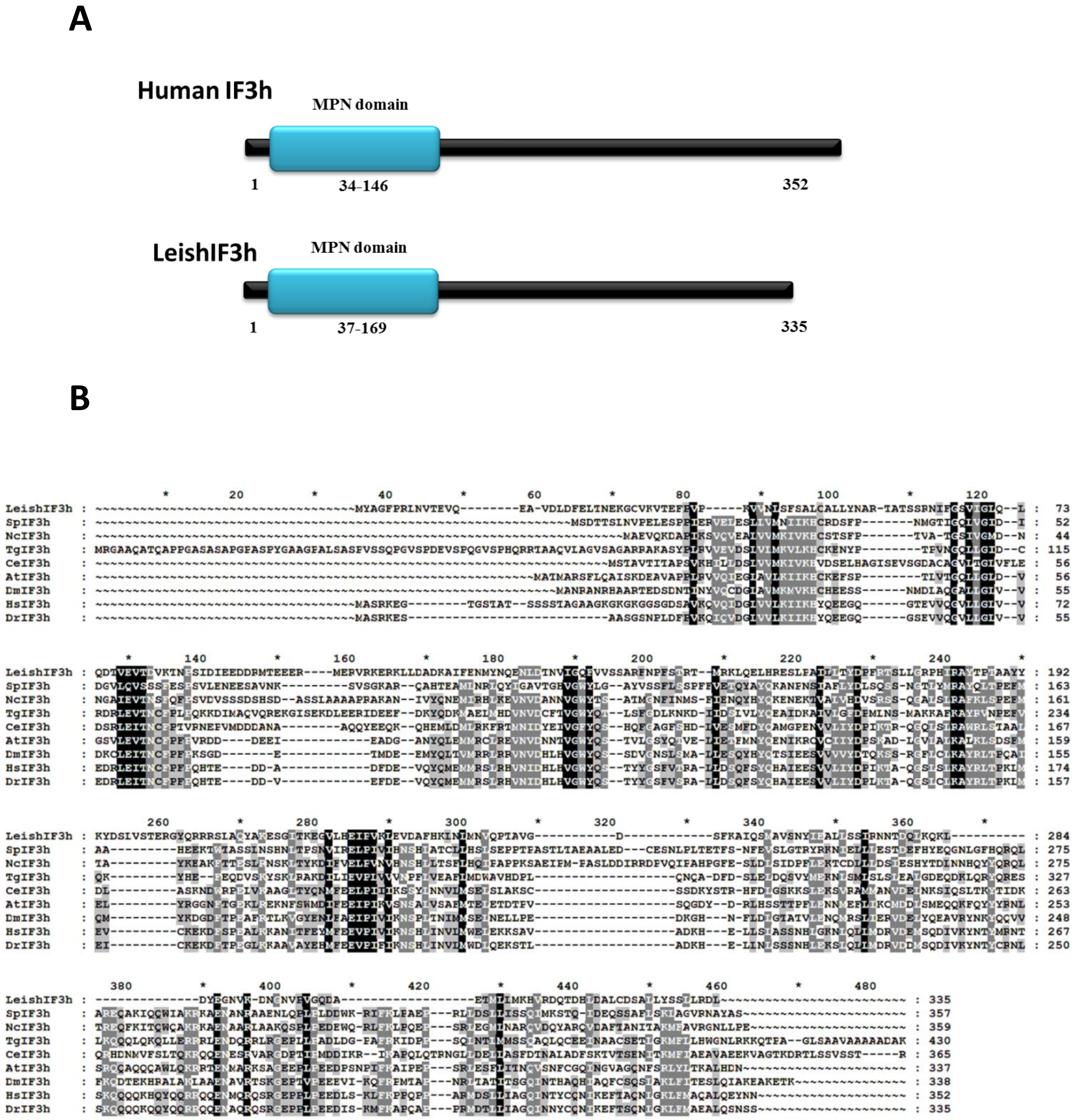

Supplemental Figure 7. A. Schematic representation of h of the *Leishmania* and human eIF3g proteins. The purple box marks the RRM domain. **B.** Multiple sequence alignment of LeishIF3g with its orthologs from different organisms: *Leishmania mexicana* (LmxM33.2700), *Toxoplasma gondii* (TgIF3g, XP_002370236.1), *Neurospora crassa* (NcIF3g, XP_962716.2), *Schizosaccharomyces pombe* (SpIF3g, NP_595727.1), *Caenorhabditis elegans* (CeIF3g, NP_495778.1), *Arabidopsis thaliana* (AtIF3g, NP_187747.1), *Drosophila melanogaster* (DmIF3g, NP_570011.1), *Danio rerio* (DrIF3g, NP_957293.1), *Homo sapiens* (HsIF3g, NP_003746.2), *Saccharomyces cerevisiae* (ScIF3g, NP_010717.1)

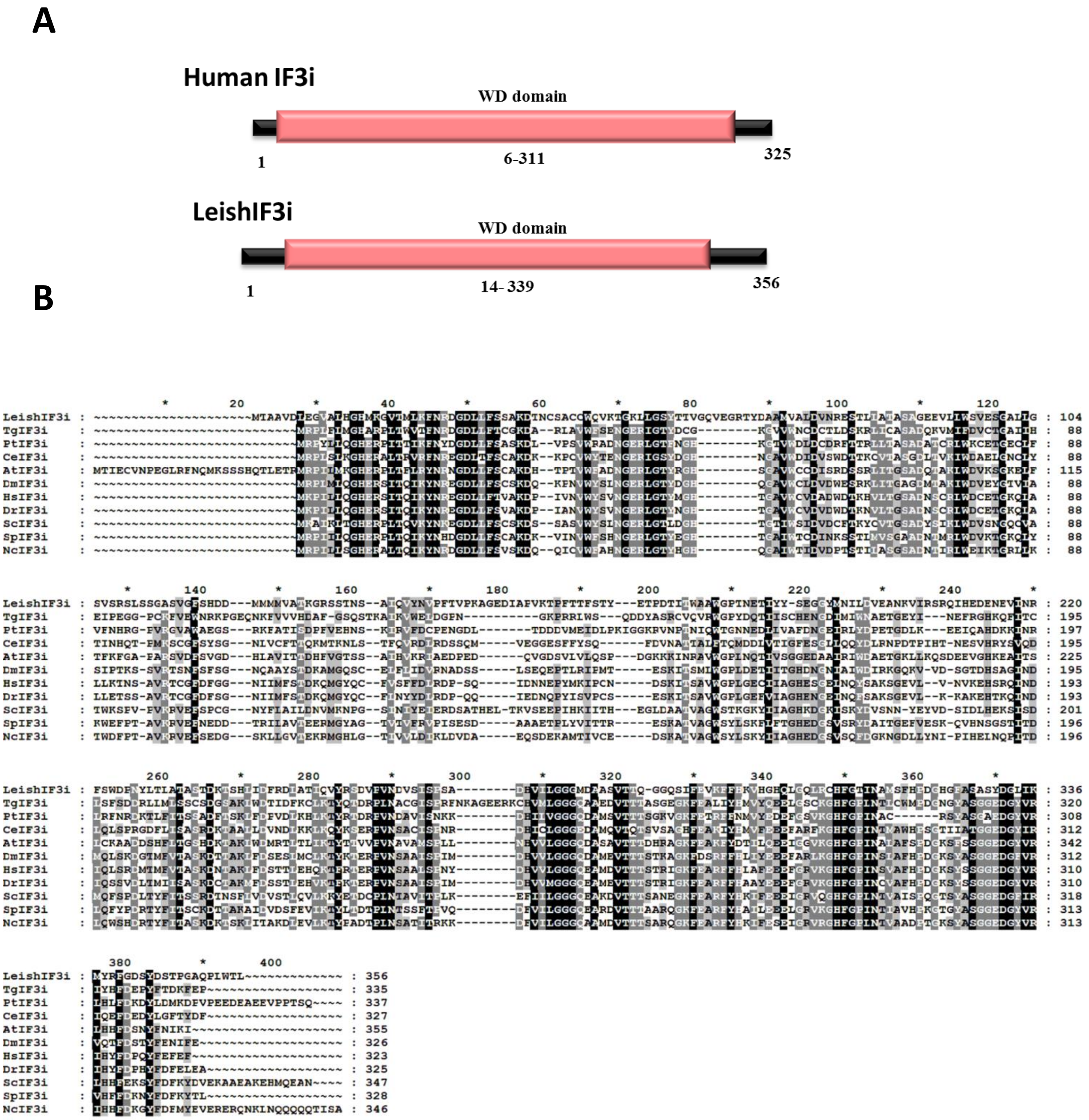

Supplemental Figure 9. A. Schematic representation of the *Leishmania* and human eIF3i proteins. The pink boxes mark the WD domain. **B**. Multiple sequence alignment of LeishIF3i (LmMx.36.3880) with its orthologs from different organisms: *Leishmania mexicana* (LmxM36.3880), *Toxoplasma gondii* (TgIF3i, XP_002368408.1), *Phaeodactylum tricornutum* (PtIF3i, XP_002183663.1), *Neurospora crassa* (NcIF3i, XP_956549.1), *Schizosaccharomyces pombe* (SpIF3i, NP_594958.1), *Caenorhabditis elegans* (CeIF3i, NP_490988.2), *Arabidopsis thaliana* (AtIF3i, NP_182152.2), *Drosophila melanogaster* (DmIF3i, NP_523478.1), *Danio rerio* (DrIF3i, NP_998155.1), *Homo sapiens* (HsIF3i, NP_003748.1) *Saccharomyces cerevisiae* (ScIF3i, NP_013866.1)

A

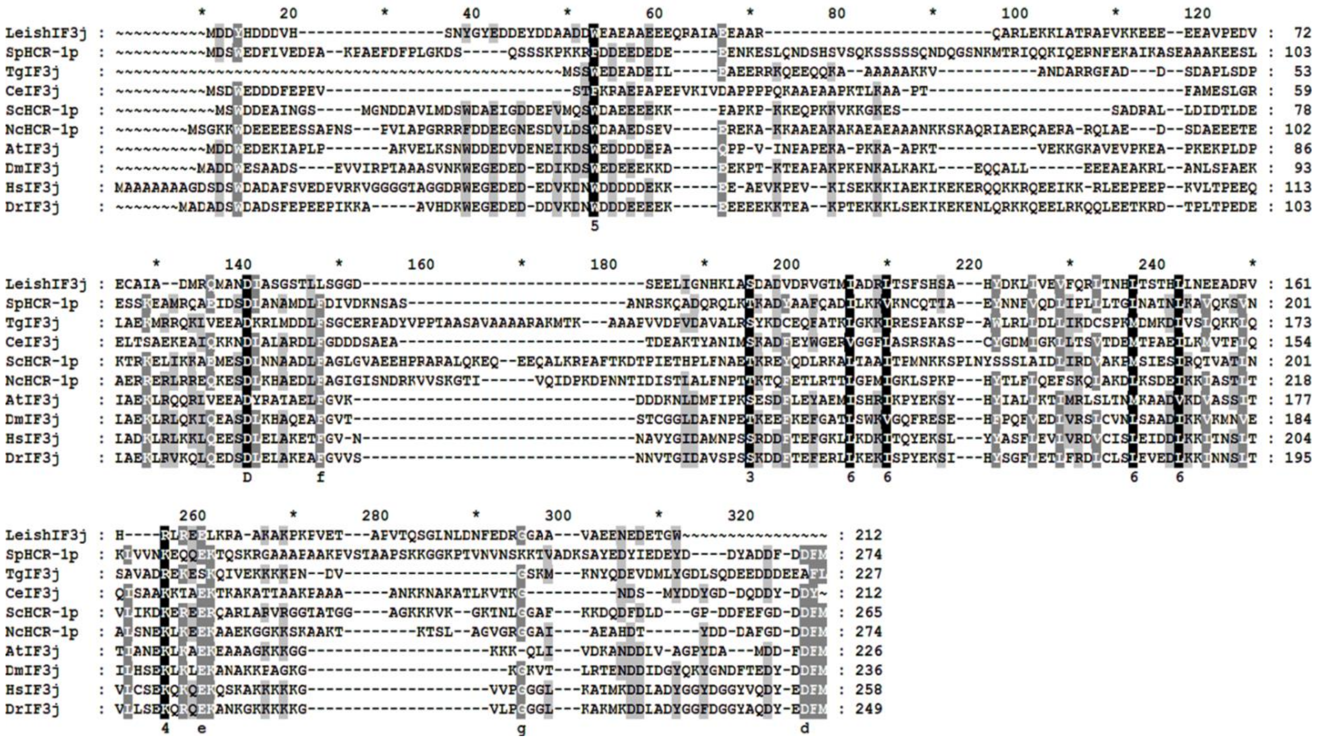

Supplemental Figure 10. A. Multiple sequence alignment of LeishIF3j with its orthologs from different organisms. *Leishmania mexicana* (LmxM25.2120), *Toxoplasma gondii* (TgIF3j, XP_002366351), *Neurospora crassa* (NcHCR-1p, Q7S931), *Schizosaccharomyces pombe* (SpHCR-1p, P87128), *Caenorhabditis elegans* (CeIF3j, NP_493365.1), *Arabidopsis thaliana* (AtIF3j, Q9C8D8), *Drosophila melanogaster* (DmIF3j, NP_610541.1), *Danio rerio* (DrIF3j, NP_957508.1), *Homo sapiens* (HsIF3j, NP_003749.2) *Saccharomyces cerevisiae* (ScHCR-1p, NP_013293.1

A

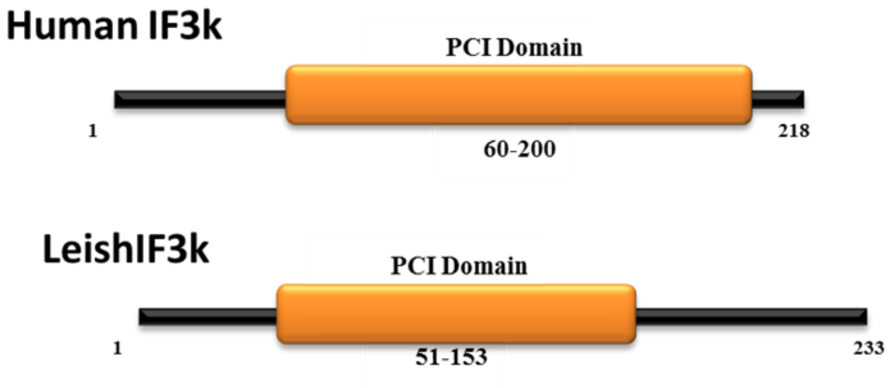

B

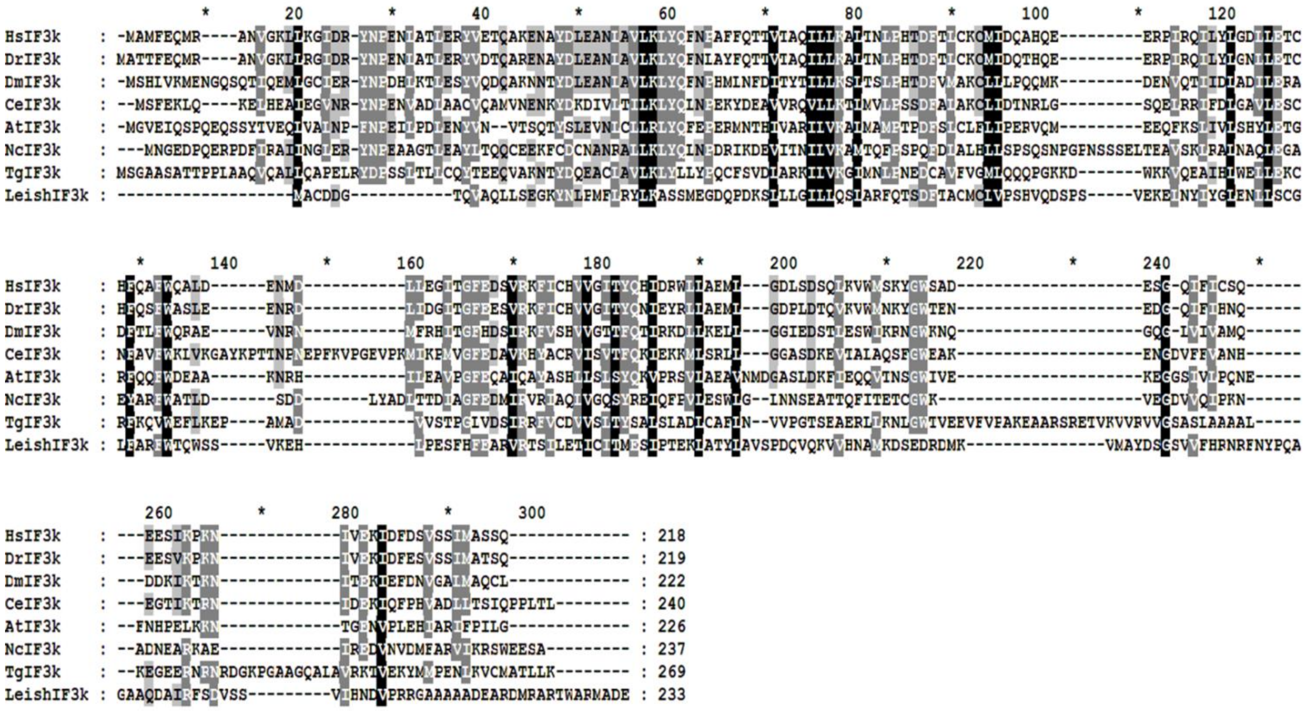

Supplemental Figure 11. A. Schematic representation of the *Leishmania* and human eIF3k proteins. The orange box marks the PCI domain **B.** Multiple sequence alignment of LeishIF3k (LmMx.31.2180) with its orthologs from different organisms: *Leishmania mexicana* (LmxM31.2180), *Toxoplasma gondii* (TgIF3k, TGME49_262040), *Neurospora crassa* (NcIF3k, XP_958946.1), *Caenorhabditis elegans* (CeIF3k, NP_506241.1), *Arabidopsis thaliana* (AtIF3k, NP_195051.1), *Drosophila melanogaster* (DmIF3k, NP_611604.1), *Danio rerio* (DrIF3k, NP_001017583.1) and *Homo sapiens* (HsIF3k, NP_037366).

A

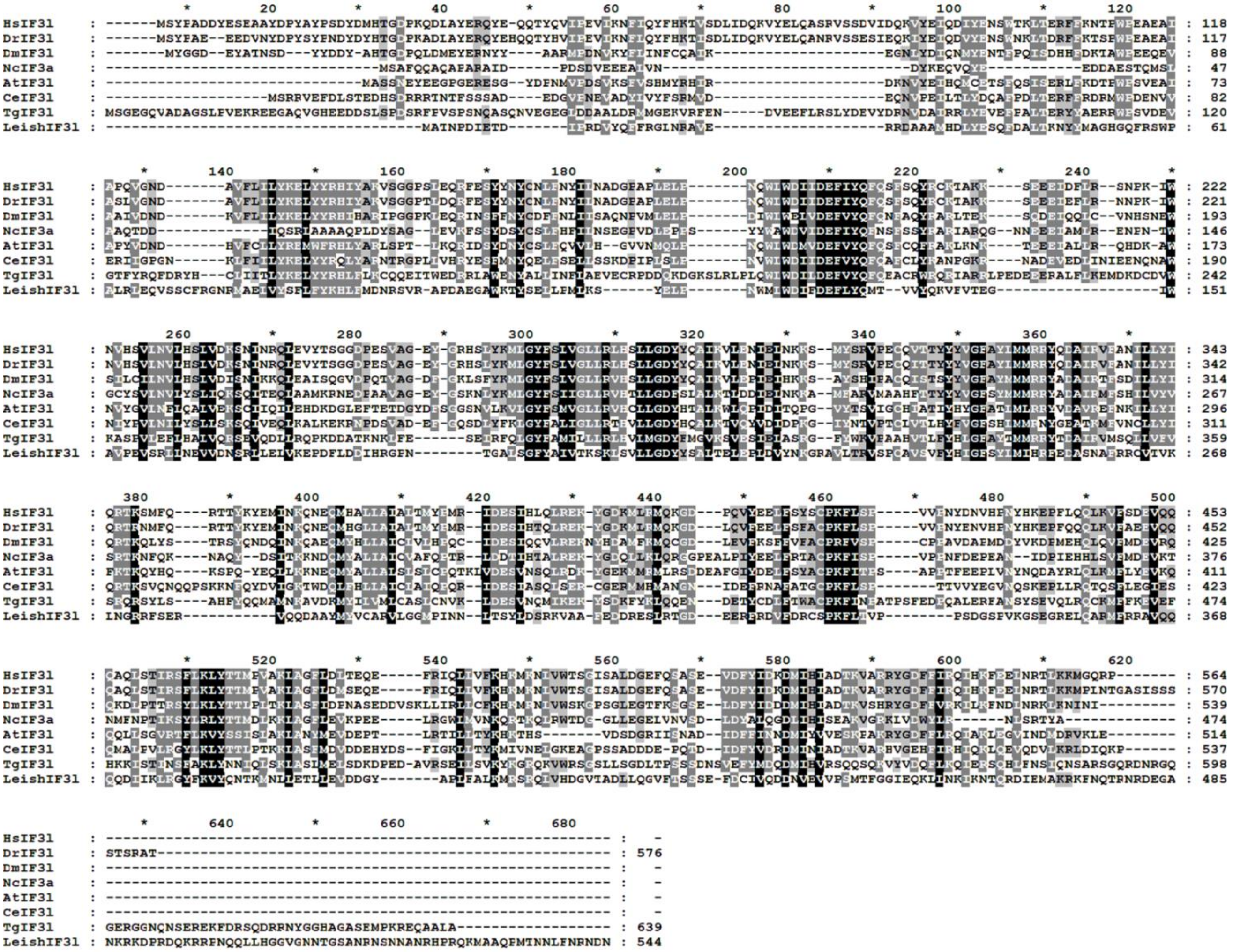

Supplemental Figure 12. Specific domains were difficult to identify. **B**. Multiple sequence alignment of LeishIF3l (LmMx.36.0250) with its orthologs from different organisms: *Leishmania mexicana* (LeishIF3l, LmxM36.0250), *Toxoplasma gondii* (TgIF3l, XP_002365947.1), *Neurospora crassa* (NcIF3l, XP_962883.1), *Caenorhabditis elegans* (Q95QW0), *Arabidopsis thaliana* (NP_680222.1), *Drosophila melanogaster* (NP_648553.1), *Danio rerio*(NP_998293.1), *Homo sapiens* (NP_057175.1)
