# Supplementary material for: The eIF3 complex of Leishmania—subunit composition and mode of recruitment to different cap-binding complexes
Source: Nucleic Acids Res. 2015 Jun 19;43(13):6222–35. doi: 10.1093/nar/gkv564 (PMC4513851; doi:10.1093/nar/gkv564)
Supplement: SUPPLEMENTARY DATA [file supp_gkv564_nar-01173-v-2015-File014.pdf]

Early branching of eIF3 subunits of *Leishmania* during evolution. **C**. A chart showing percent identity between different eIF3 subunits from *Leishmania* and other organisms.The sequences were drawn from Homologene(www. www.ncbi.nlm.nih.gov/HomoloGene/). The *Toxoplasma* eIF3 subunits which are not annotated were obtained by a BLAST search in *Toxoplasma* geneDB (www.toxodb.org/) using human eIF3 orthologs as query sequences. The hits were confirmed by reciprocal blast in NCBI. The percent identity was calculated by MUSCLE. A three color gradient formatting is applied on charts where red represent the lowest percent identities whereas green represent highest value, the average values are in yellow. **D**. Phylogenetic tree showing the evolutionary relationship between eIF3 subunits of different species. The trees were generated by phylogeny.fr (phylogeny.lirmm.fr/)

Sup.Fig 1. C

eIF3a											
L.mexicana	100										
S.cerevisae	15.56	100									
T. gondii	15.63	23.62	100								
P.tricornutum	17.21	25.03	28.77	100							
N.crassa	16.47	29.3	27.68	31.56	100						
S.prombe	17.12	30.11	25.87	29.26	45.25	100					
C. elegans	15.3	23.57	24.47	28.09	26.18	26.32	100				
A.thaliana	17.62	26.08	29.18	32.86	33.64	33.98	30.65	100			
D.melanogaster	16.96	26.07	26.77	31.16	33.48	31.65	34.21	34.92	100		
D.rerio	19.16	25.41	28.34	32.3	34.93	32.83	36.03	38.05	48.14	100	
H.sapiens	18.89	26.29	27.59	33.64	34.39	33.26	35.54	37.66	48.34	80.78	100
	L.mexicana	*S.cerevisae*	*T. gondii*	*P.tricornutum*	*N.crassa*	*S.prombe*	*C. elegans*	*A.thaliana*	*D.melanogaste*	*D.rerio*	*H.sapiens*

D

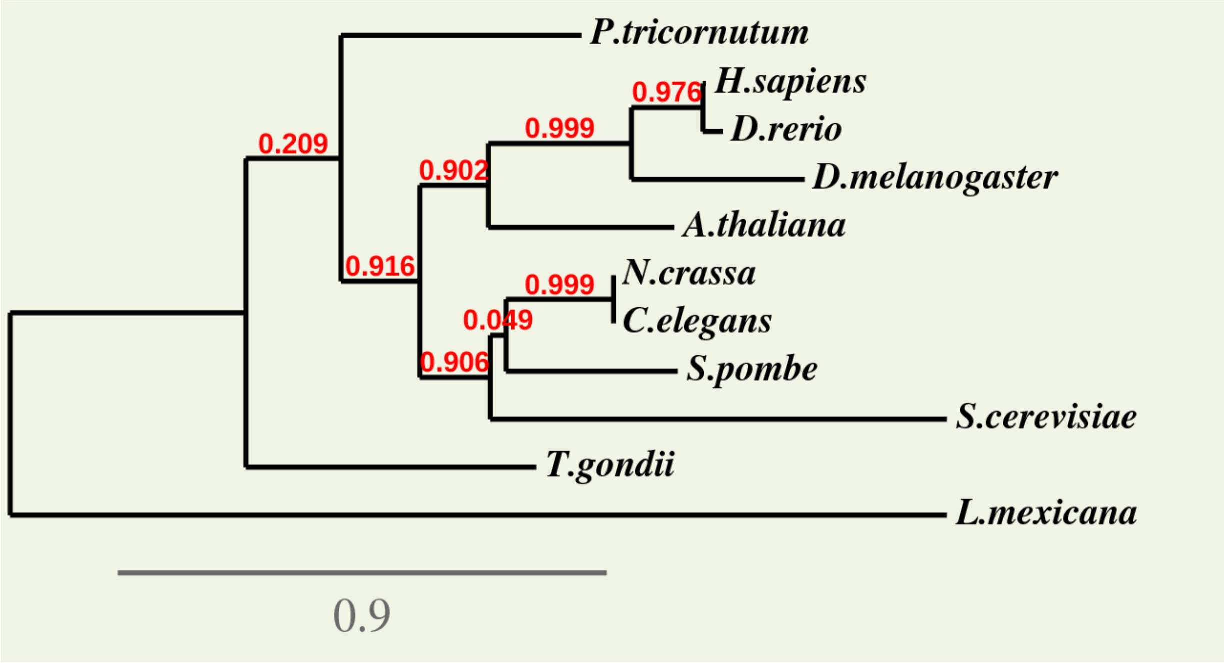

Sup.Fig 1. C. Percent of identity between full length proteins sequences of eIF3a subunits from different species. D. The phylogenetic tree showing evolutionary relationship between different eIF3a orthologs. *Leishmania Mexicana* (LmxM17.0010), *Saccharomyces cerevisiae* (NP_009635.1), *Toxoplasma gondii* (TGME49_201680), *Phaeodactylum tricornutum* (XP_002180731), Neurospora crassa (XP_956151.2), *Schizosaccharomyces pombe* (NP_596379.1), Caenorhabditis elegans (NP_498698.1), *Arabidopsis thaliana* (NP_192881.1), *Drosophila melanogaster* (NP_649470.2), *Danio rerio* (NP_956114.2), *Homo sapiens* (NP_003741.1)

Sup.Fig 2. C

eIF3b											
L.mexicana	100										
S.cerevisae	19.51	100									
T. gondii	19.24	24.19	100								
P.tricornutum	20.67	27.86	30.5	100							
N.crassa	22.42	36.95	29.32	34.83	100						
S.prombe	21.7	35.1	30.39	34.34	49.3	100					
C. elegans	20.16	23.85	26.73	28.08	27.81	28.38	100				
A.thaliana	23.99	28.63	32.31	35.13	37.08	36.55	30.62	100			
D.melanogaster	21.88	31.44	29.96	32.98	37.61	37.02	33.73	36.96	100		
D.rerio	21.97	31.14	34.29	35.98	39.15	39.51	34.18	39.82	51.42	100	
H.sapiens	18.89	30.35	34.38	35.98	40.52	39.06	35.02	39.35	52.08	86.49	100
	L.mexicana	*S.cerevisae*	*T. gondii*	*P.tricornutum*	*N.crassa*	*S.prombe*	*C. elegans*	*A.thaliana*	*D.melanogaster*	*D.rerio*	*H.sapiens*

D

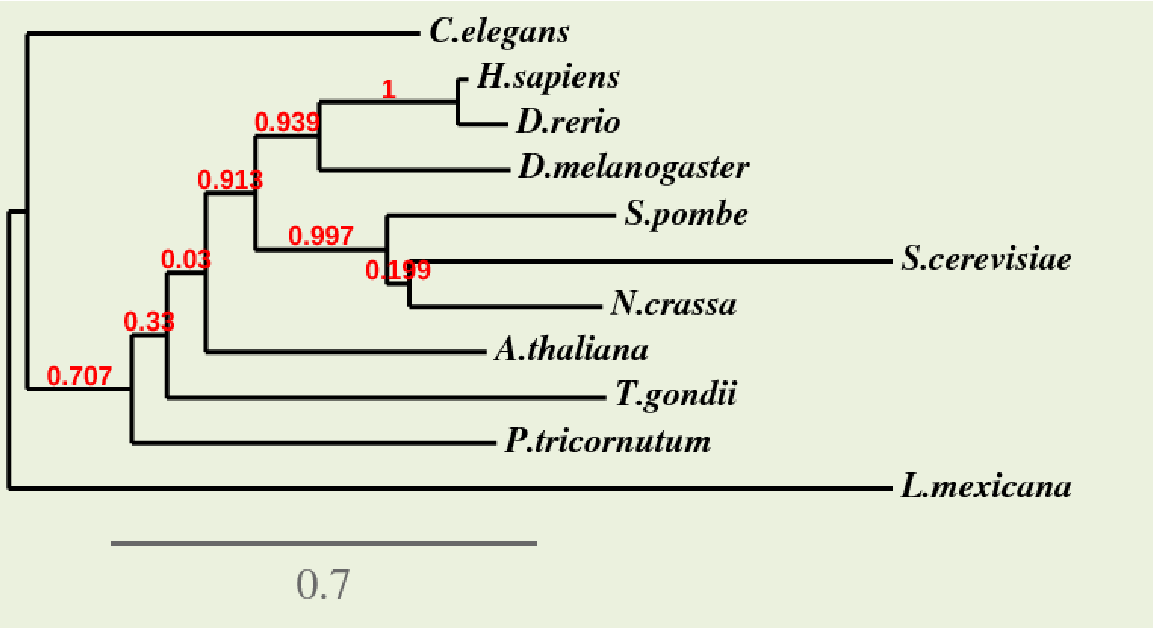

Sup.Fig 2. C. Percent of identity between full length protein sequences of eIF3b subunits from different species. D. The phylogenetic tree showing evolutionary relationship between different eIF3b orthologs. *Leishmania Mexicana* (LmxM17. 1290), *Saccharomyces cerevisiae* (NP_015006.3), *Toxoplasma gondii* (XP_002370073.1), *Phaeodactylum tricornutum* (XP_002179558.1), *Neurospora crassa* (XP_959527.1), *Schizosaccharomyces pombe* (NP_594528.1), *Caenorhabditis elegans* (NP_001022469.2), *Arabidopsis thaliana* (NP_568498.1), *Drosophila melanogaster* (NP_725691.1), *Danio rerio* (NP_001277022.1), *Homo sapiens* (NP_001032360.1)

Sup.Fig 3. C

eIF3c											
L.mexicana	100										
S.cerevisae	16.18	100									
T. gondii	20.36	26.15	100								
P.tricornutum	20.18	27.43	36.09	100							
N.crassa	18.86	32.57	33.66	35.28	100						
S.prombe	17.74	33.12	33.17	32.81	46.18	100					
C. elegans	20.94	27.11	30.41	36.63	35.03	31.87	100				
A.thaliana	20.31	27.81	32.43	37.95	35.19	36.04	36.22	100			
D.melanogaster	19.97	27.46	29.79	34.79	35.58	35.17	42.51	36.3	100		
D.rerio	21.11	29.26	32.54	38.43	38.61	37.05	44.94	38.23	53.44	100	
H.sapiens	19.8	29.08	31.95	38.23	38.1	37.97	44.9	38.76	52.69	77.18	100
	L.mexicana	*S.cerevisae*	*T. gondii*	*P.tricornutum*	*N.crassa*	*S.prombe*	*C. elegans*	*A.thaliana*	*D.melanogaster*	*D.rerio*	*H.sapiens*

D

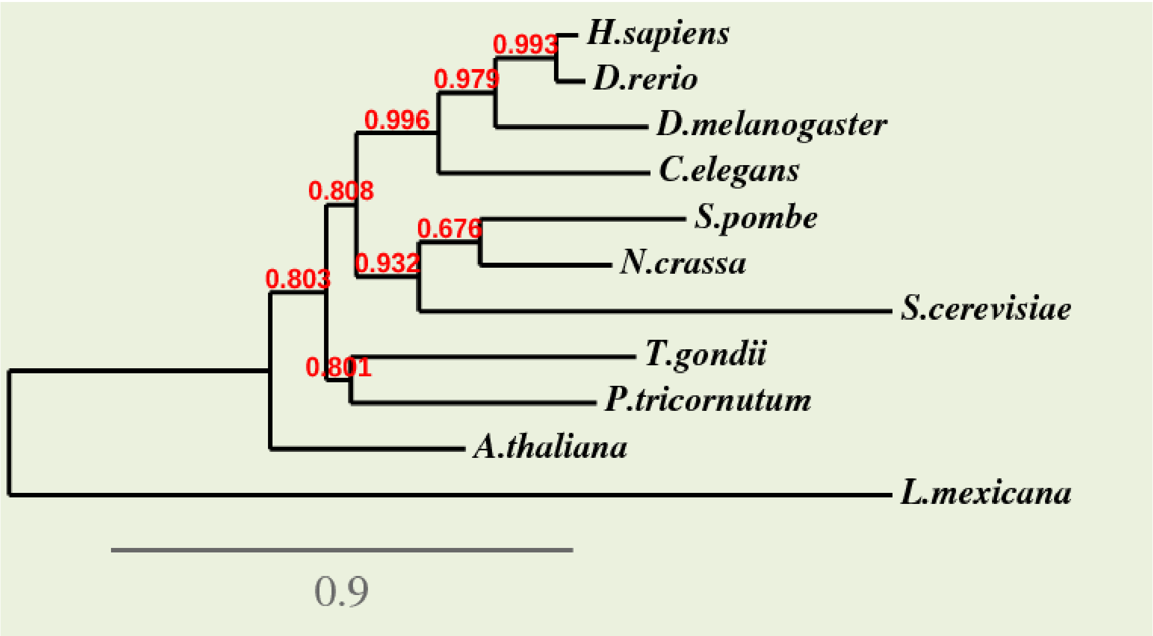

Sup.Fig 3. C. Percent of identity between full length protein sequences of eIF3c subunits from different species. D. Phylogenetic tree showing evolutionary relationship between different eIF3c orthologs. *Leishmania mexicana* (LmxM36.6980), *Saccharomyces cerevisiae* (NP_014040.1), *Toxoplasma gondii* (XP_002370231.1), *Phaeodactylum tricornutum* (XP_002183081.1), *Neurospora crassa* (XP_962952.1), *Schizosaccharomyces pombe* (NP_593828.2), *Caenorhabditis elegans* (NP_492638.1), *Arabidopsis thaliana* (NP_188926.4), *Drosophila melanogaster* (NP_611242.1), *Danio rerio* (NP_998628.1), *Homo sapiens* (NP_003743.1).

Sup.Fig 4. C

eIF3d										
L.mexicana	100									
T. gondii	22.74	100								
P.tricornutum	29.34	38.23	100							
N.crassa	24.01	31.04	43.36	100						
S.prombe	25.81	33.21	44.05	50	100					
C. elegans	24.75	29.21	42.9	35.14	36.86	100				
A.thaliana	25.96	34.77	47.93	40.04	35.37	39.52	100			
D.melanogaster	23.59	31.16	45.9	39.93	41.56	45.17	41.96	100		
D.rerio	26.61	32.76	47.14	39.23	40.89	44.38	43.74	57.83	100	
H.sapiens	26.81	32.82	47.68	39.56	41.42	45.93	44.01	57.38	90.88	100
	L.mexicana	***T. gondii***	***P.tricornutum***	***N.crassa***	***S.prombe***	***C. elegans***	***A.thaliana***	***D.melanoga***	***D.rerio***	***H.sapiens***

D

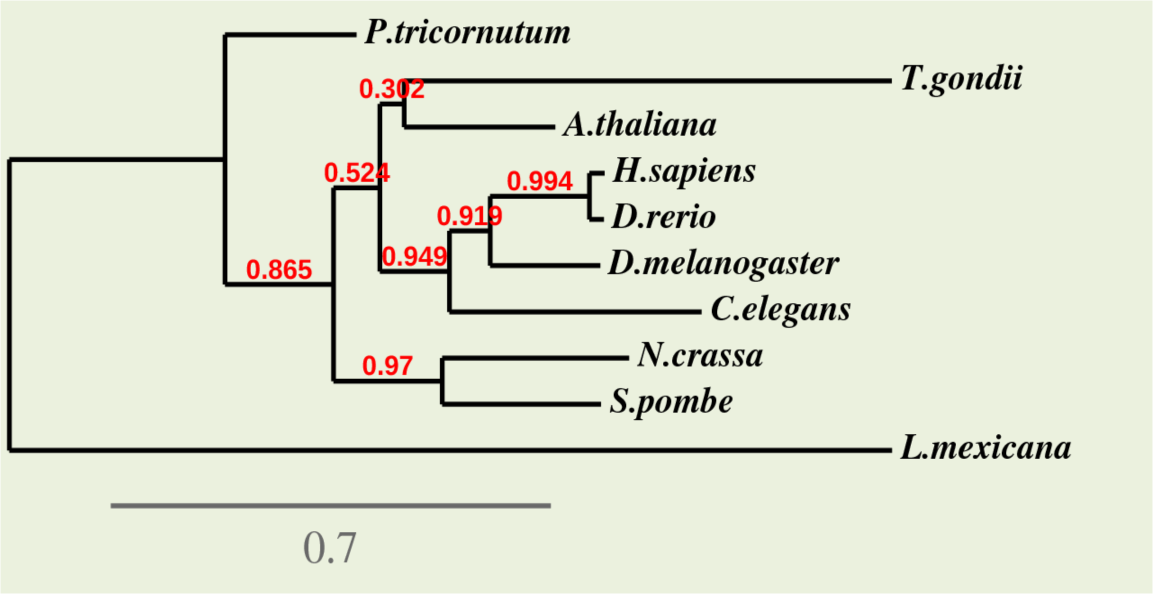

Sup.Fig 4. C. Percent of identity between full length protein sequences of eIF3d subunits from different species. D. Evolutionary relationship between eIF3d orthologs from different organisms. *Leishmania Mexicana* (LmxM29.3040), *Toxoplasma gondii* (XP_002372060.1), *Phaeodactylum tricornutum* (XP_002183081.1), *Neurospora crassa* (XP_958873.1), *Schizosaccharomyces pombe* (NP_594625.1), *Caenorhabditis elegans* (NP_498984.1), *Arabidopsis thaliana* (NP_193830.1), *Drosophila melanogaster* (NP_524463.2), *Danio rerio* (NP_956310.1), *Homo sapiens* (NP_003744.1)

Sup.Fig 5. C

eIF3e										
L.mexicana	100									
T. gondii	21.68	100								
P.tricornutum	25.41	26.47	100							
N.crassa	27.18	27.19	36.3	100						
S.prombe	26.09	24.59	33.17	43.45	100					
C. elegans	22.73	27.73	38.73	37.35	35.53	100				
A.thaliana	25.38	27.38	40.25	39.67	40.05	40.38	100			
D.melanogaster	26.67	27.93	38.4	37.44	35.14	44.21	44.66	100		
D.rerio	27.92	29.26	42.89	44.66	42.69	50.7	52.24	60.37	100	
H.sapiens	27.41	29.26	43.73	43.81	43.02	50.23	54.01	60.51	93.26	100
	L.mexicana	*T. gondii*	*P.tricornutum*	*N.crassa*	*S.prombe*	*C. elegans*	*A.thaliana*	*D.melanoga*	*D.rerio*	*H.sapiens*

D

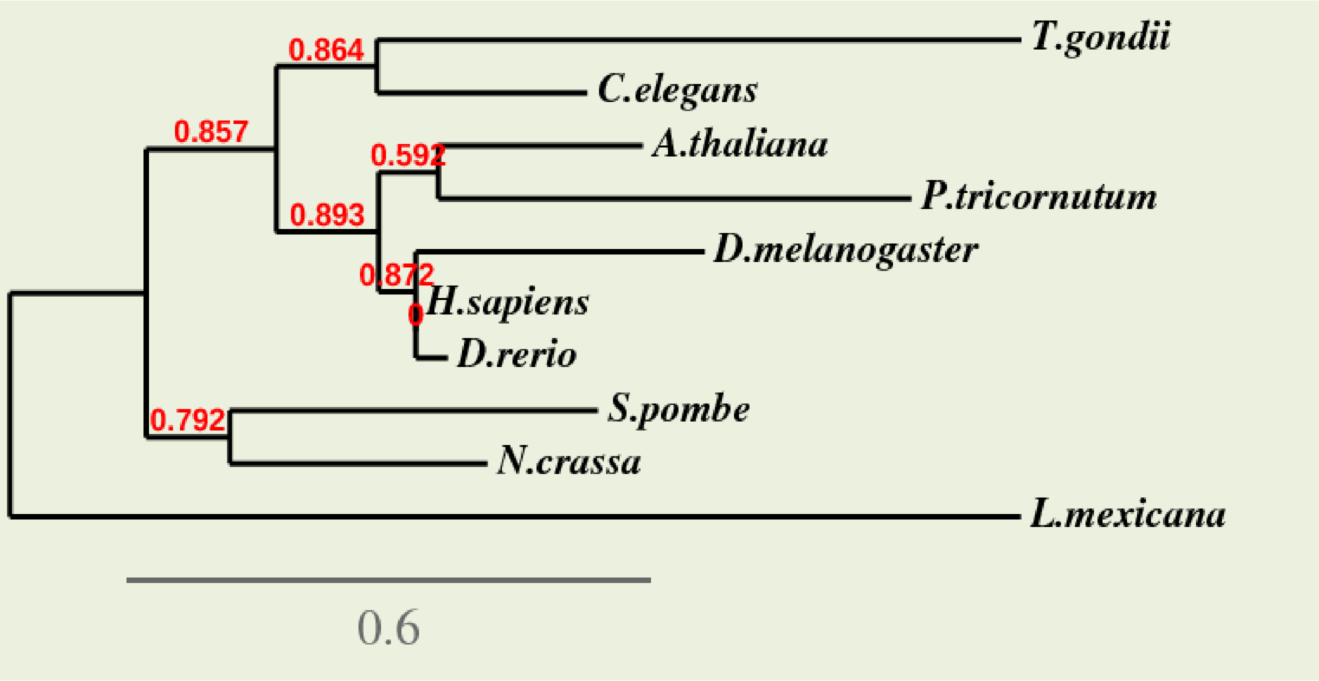

Sup.Fig 5. C. Percent of identity between full length protein sequences of eIF3e subunits from different species. D. Phylogenetic tree showing evolutionary relationship between eIF3e subunits from different organisms. *Leishmania Mexicana* (LmxM28.2310), *Toxoplasma gondii* (XP_002365953.1), *Phaeodactylum tricornutum* (XP_002181668.1), *Neurospora crassa* (XP_959880.1), *Schizosaccharomyces pombe* (NP_595367.1), *Caenorhabditis elegans* (NP_492785.1), *Arabidopsis thaliana* (NP_567047.1), *Drosophila melanogaster* (NP_477385.1), *Danio rerio* (NP_957133.1), *Homo sapiens* (NP_001559.1)

Sup.Fig 6. C

eIF3f										
L.mexicana	100									
T. gondii	14.44	100								
P.tricornutum	14.79	33.83	100							
N.crassa	17.41	22.92	28.52	100						
S.prombe	18.08	26.94	31.84	42.57	100					
C. elegans	12.04	25.09	29.7	26.16	26.69	100				
A.thaliana	15	28.37	39.77	30.48	33.92	29.96	100			
D.melanogaster	14.61	28.52	37.55	26.91	31.99	34.93	38.01	100		
D.rerio	17.31	31.73	36.84	30.51	36.16	36.4	39.18	49.08	100	
H.sapiens	18.46	31.08	35.71	29.83	32.87	37.13	36.46	47.99	79.12	100
	L.mexicana	*T. gondii*	*P.tricornutum*	*N.crassa*	*S.prombe*	*C. elegans*	*A.thaliana*	*D.melanoga*	*D.rerio*	*H.sapiens*

D

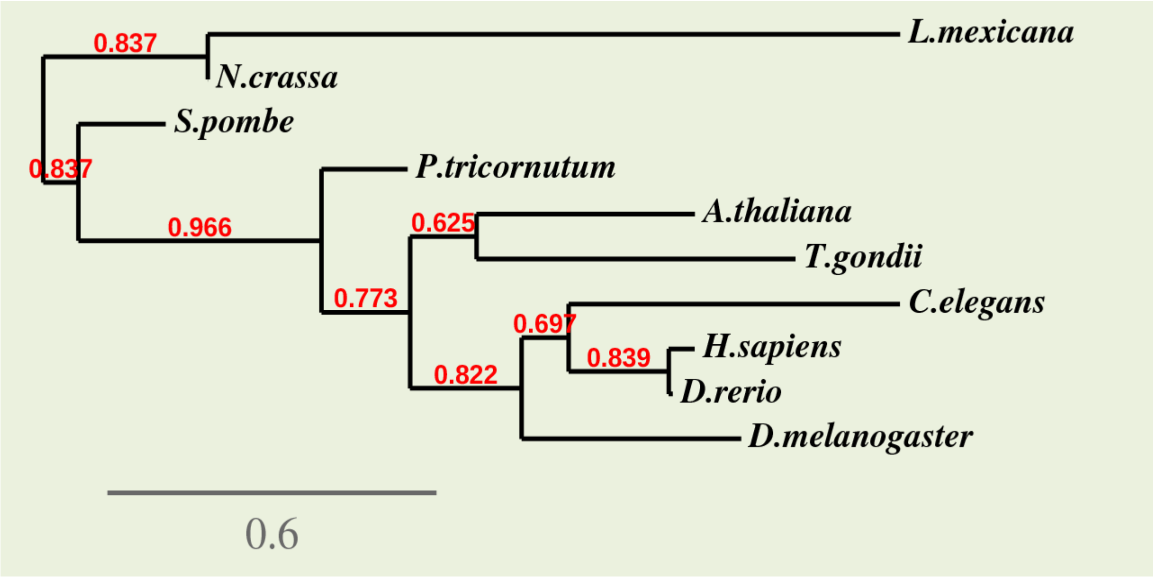

Sup.Fig 6. C. Percent of identity between full length protein sequences of eIF3f subunits from different species. D. Phylogenetic tree showing evolutionary relationship between different eIF3f orthologs. *Leishmania mexicana* (LmxM25.1610), *Toxoplasma gondii* (XP_002366441), *Phaeodactylum tricornutum* (EEC49441.1), *Neurospora crassa* (XP_961633.1), *Schizosaccharomyces pombe* (NP_596298.1), *Caenorhabditis elegans* (NP_495988.1), *Arabidopsis thaliana* (NP_181528.1), *Drosophila melanogaster* (NP_649489.1), *Danio rerio* (NP_001186938.1), *Homo sapiens* (NP_003745.1)

Sup.Fig 7. C

eIF3g											
L.mexicana	100										
S.cerevisae	17.26	100									
T. gondii	20.51	26.19	100								
P.tricornutum	26.51	41.67	50	100							
N.crassa	18.93	32.45	24.81	52.38	100						
S.prombe	23.4	35.14	28.35	55.95	43.57	100					
C. elegans	20.47	33.05	27.98	47.62	32.02	33.33	100				
A.thaliana	17.84	33.72	30.68	57.14	34.98	37.87	28.97	100			
D.melanogaster	18.57	29.03	30.56	51.19	38.78	31.64	39.26	34.62	100		
D.rerio	19.01	29.81	33.33	64.29	40.35	39.34	36.22	38.65	48.88	100	
H.sapiens	21.58	32.08	33.58	65.48	37.89	40.07	36.61	39.86	47.01	87.29	100
	L.mexicana	*S.cerevisae*	*T. gondii*	*P.tricornutum*	*N.crassa*	*S.prombe*	*C. elegans*	*A.thaliana*	*D.melanogaster*	*D.rerio*	*H.sapiens*

D

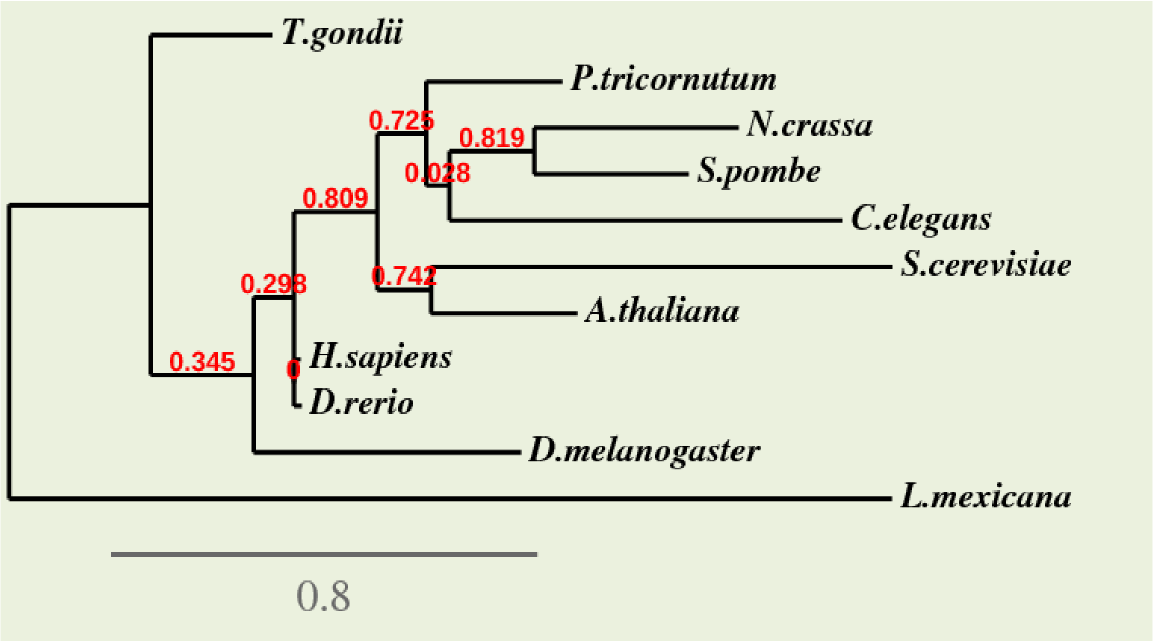

Sup.Fig 7. C. Percent of identity between full length protein sequences of eIF3g subunits from different species. D. Phylogenetic tree showing evolutionary relationship between different eIF3g orthologs. (LmxM33.2700), *Toxoplasma gondii* (XP_002370236.1), *Phaeodactylum tricornutum* (XP_002184494.1), *Neurospora crassa* (XP_962716.2), *Schizosaccharomyces pombe* (NP_595727.1), *Caenorhabditis elegans* (NP_495778.1), *Arabidopsis thaliana* (NP_187747.1), *Drosophila melanogaster* (NP_570011.1), *Danio rerio* (NP_957293.1), *Homo sapiens* (NP_003746.2), *Saccharomyces cerevisiae* (NP_010717.1).

Sup.Fig 8. C

eIF3h										
L.mexicana	100									
T. gondii	17.89	100								
P.tricornutum	18.27	29.76	100							
N.crassa	16.78	27.81	27.6	100						
S.prombe	15.09	22.62	25.7	37.46	100					
C. elegans	14.19	20.81	29.27	24.77	24.09	100				
A.thaliana	14.18	32.13	39.18	36.34	28.35	29.01	100			
D.melanogaster	13.62	27.03	35.26	32.4	26.3	32.42	35.56	100		
D.rerio	16.73	29.6	41.15	33.75	27.96	39.14	40.79	46.25	100	
H.sapiens	16.49	31.12	40.1	33.13	27.05	38.53	39.82	45.35	83.88	100
	L.mexicana	*T. gondii*	*P.tricornutum*	*N.crassa*	*S.prombe*	*C. elegans*	*A.thaliana*	*D.melanoga*	*D.rerio*	*H.sapiens*

D

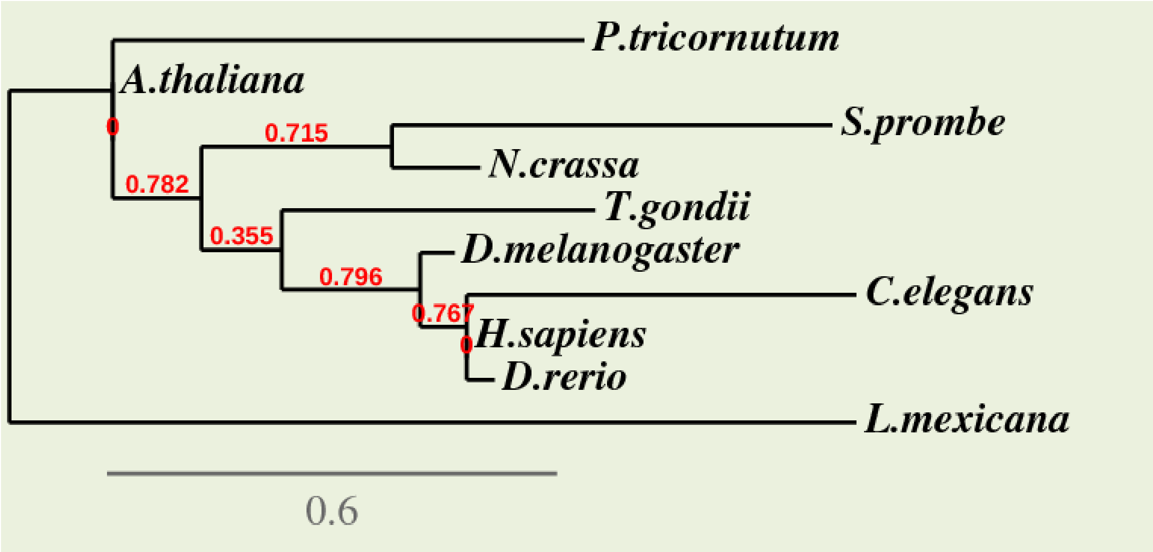

Sup.Fig 8. C. Percent of identity between full length protein sequences of eIF3h subunits from different species. D. Phylogenetic tree showing evolutionary relationship between different eIF3h orthologs. *Leishmania mexicana* (LmxM07.0640), *Toxoplasma gondii* (XP_002367391.1), *Phaeodactylum tricornutum* (XP_002184494.1), *Neurospora crassa*(XP_962445.1), *Schizosaccharomyces pombe* (NP_593158.1), *Caenorhabditis elegans* (NP_491370.1), *Arabidopsis thaliana*(NP_563880.1), *Drosophila melanogaster* (NP_524834.2), *Danio rerio* (NP_001003763.1), *Homo sapiens* (NP_003747.1) *Saccharomyces cerevisiae* (NP_010717.1)

Sup.Fig 9. C

eIF3i											
L.mexicana	100										
S.cerevisae	28.44	100									
T. gondii	28.66	36.84	100								
P.tricornutum	31.45	41.19	38.8	100							
N.crassa	31.6	54.41	39.25	43.98	100						
S.prombe	32.21	52.76	41.19	41.19	60.98	100					
C. elegans	29.94	39.57	37.69	36.79	40.62	36.92	100				
A.thaliana	34.64	40	43.61	43.71	44.31	45.23	37.35	100			
D.melanogaster	33.13	44.17	40.31	42.9	45.23	44.31	44.31	46.13	100		
D.rerio	30.65	45.85	37.38	44.03	50.15	48.62	42.15	44.89	60.49	100	
H.sapiens	30.03	45.85	38.63	44.34	51.69	48.92	43.08	46.13	60.8	83.69	100
	L.mexicana	*S.cerevisae*	*T. gondii*	*P.tricornutum*	*N.crassa*	*S.prombe*	*C. elegans*	*A.thaliana*	*D.melanogaster*	*D.rerio*	*H.sapiens*

D

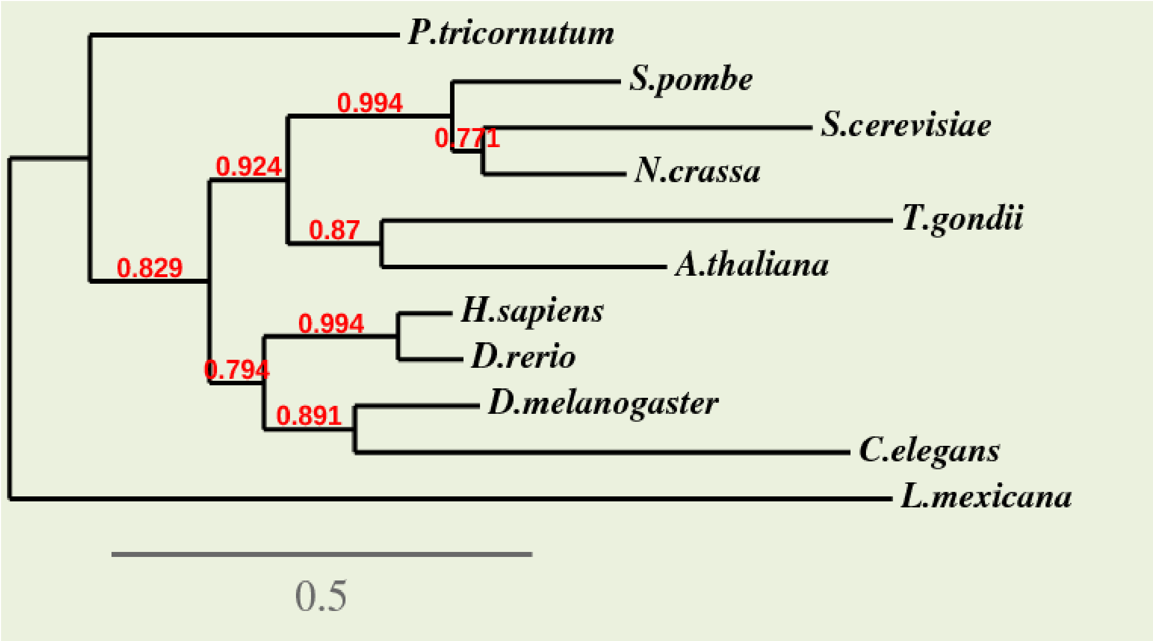

Sup.Fig 9. C. Percent of identity between protein sequences of eIF3i subunits from different species.

D. Phylogenetic tree showing evolutionary relationship between different eIF3i orthologs. *Leishmania mexicana* (LmxM36.3880), *Toxoplasma gondii* (XP_002368408.1), *Phaeodactylum tricornutum* (XP_002183663.1), *Neurospora crassa* (XP_956549.1), *Schizosaccharomyces pombe* (NP_594958.1), *Caenorhabditis elegans* (NP_490988.2), *Arabidopsis thaliana* (NP_182152.2), *Drosophila elanogaster* (NP_523478.1), *Danio rerio* (NP_998155.1), *Homo sapiens* (NP_003748.1), *Saccharomyces cerevisiae* (NP_013866.1)

Sup.Fig 10. C

eIF3j										
L.mexicana	100									
S.cerevisae **D**	11.88	100								
T. gondii	19.35	21.96	100							
N.crassa	13.94	29.2	17.97	100						
S.prombe	13.88	20.78	18.63	20.83	100					
C. elegans	19.44	20.73	19.05	21.29	24.12	100				
A.thaliana	17	23.22	23.4	26.24	21.72	22.56	100			
D.melanogaster	19.12	26.39	20	27.04	23.81	23.59	31.36	100		
D.rerio	18.93	29.09	26.53	24.46	23.87	22.61	30.94	40.09	100	
H.sapiens	18.54	28.31	25.26	24.89	22.08	22.08	30.94	39.06	67.89	100
	L.mexicana	*S.cerevisae*	*T. gondii*	*N.crassa*	*S.prombe*	*C. elegans*	*A.thalian*	*D.melanog*	*D.rerio*	*H.sapiens*

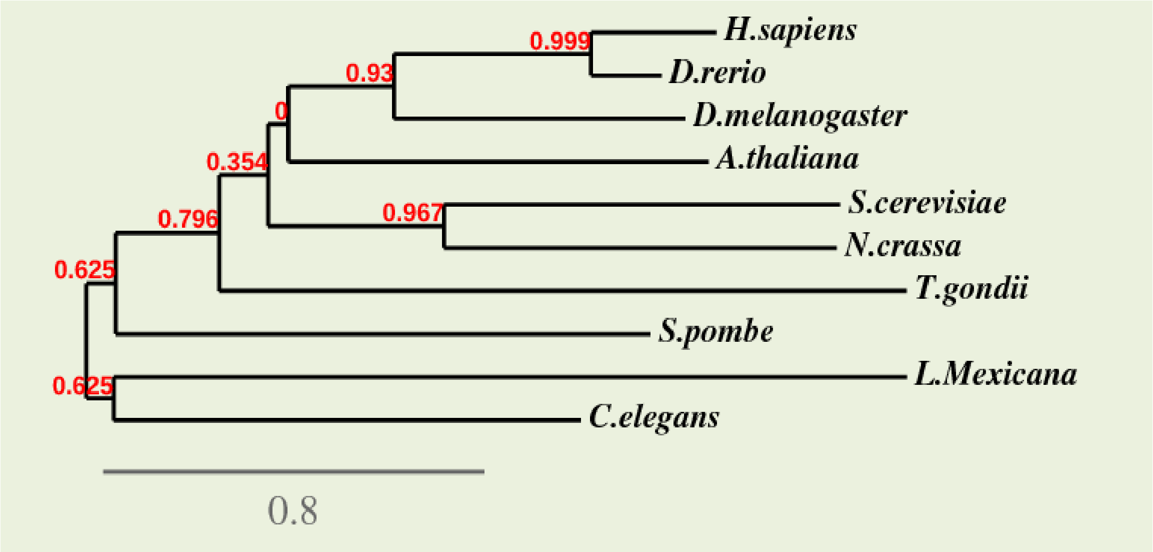

Sup.Fig 10. C. Percent of identity between protein sequences of eIF3j subunits from different species. D. Phylogenetic tree showing evolutionary relationship between different eIF3j orthologs. *Leishmania mexicana* (LmxM25.2120), *Toxoplasma gondii* (XP_002366351), *Phaeodactylum tricornutum* (XP_002183663.1), *Neurospora crassa* (Q7S931), *Schizosaccharomyces pombe* (P87128), *Caenorhabditis elegans* (NP_493365.1), *Arabidopsis thaliana* (Q9C8D8), *Drosophila melanogaster* (NP_610541.1), *Danio rerio* (NP_957508.1), *Homo sapiens* (NP_003749.2), *Saccharomyces cerevisiae* (NP_013293.1)

Sup.Fig 11. C

eIF3k								
L.mexicana	100							
T. gondii	13.81	100						
N.crassa	15.92	21.93	100					
C. elegans	21.61	21.88	22.32	100				
A.thaliana	17.62	20.91	24.88	25.58	100			
D.melanogaster	17.1	28.38	28.77	33.64	29.55	100		
D.rerio	25.39	26.94	28.31	37.33	31.48	43.12	100	
H.sapiens	25.39	28.44	28.9	38.25	32.41	46.79	83.49	100
	L.mexicana	*T. gondii*	*N.crassa*	*C. elegans*	*A.thaliana*	*D.melanogc*	*D.rerio*	*H.sapiens*

D

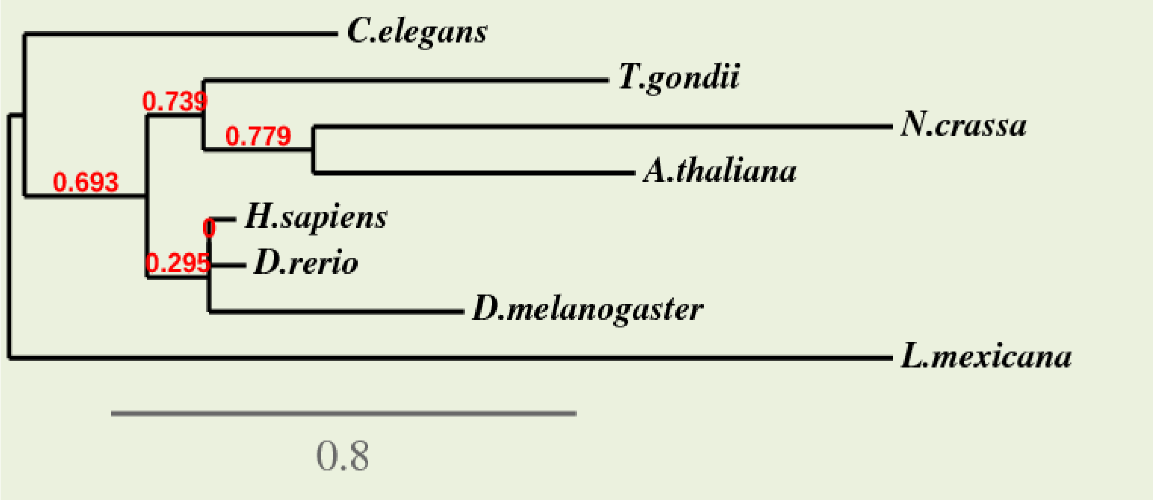

Sup.Fig 11. C. Percent of identity between full length protein sequences of eIF3k subunits from different species. D. Phylogenetic tree showing evolutionary relationship between eIF3k subunits from different organisms. *Leishmania Mexicana* (LmxM31.2180), *Toxoplasma gondii* (TGME49_262040), *Neurospora crassa* (XP_958946.1), *Caenorhabditis elegans* (NP_506241.1), *Arabidopsis thaliana* (NP_195051.1), *Drosophila melanogaster* (NP_611604.1), *Danio rerio* (NP_001017583.1), *Homo sapiens* (NP_037366.1)

Sup.Fig 12. C

eIF3l								
L.mexicana	100							
T. gondii	24.75	100						
N.crassa	21.55	30.26	100					
C. elegans	22.51	28.98	34.9	100				
A.thaliana	24.5	33.86	35.59	35.59	100			
D.melanogaster	22.83	30.45	40.17	39.16	41.98	100		
D.rerio	23.58	31.39	45.94	42.02	45.26	56.42	100	
H.sapiens	24.24	32.77	46.79	42.1	46.05	55.64	90.04	100
	L.mexicana	*T. gondii*	*N.crassa*	*C. elegans*	*A.thaliana*	*D.melanoga*	*D.rerio*	*H.sapiens*

D

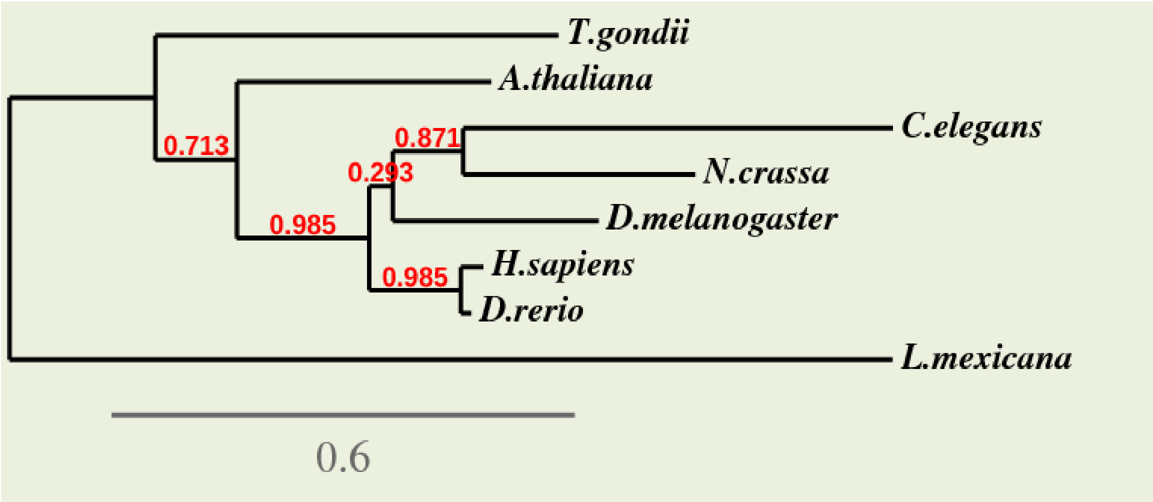

Sup.Fig 12. C Percent of identity between full length protein sequences of eIF3l subunits from different species D. Phylogenetic tree showing evolutionary relationship between eIF3l subunits from different organisms. *Leishmania Mexicana* (LmxM36.0250), , *Toxoplasma gondii* (XP_002365947.1), *Neurospora crassa* (XP_962883.1), *Caenorhabditis elegans* (Q95QW0), *Arabidopsis thaliana* (NP_680222.1), *Drosophila melanogaster* (NP_648553.1), *Danio rerio* (NP_998293.1), *Homo sapiens* (NP_057175.1)
