# Supplementary figures and images for: The eIF3 complex of Leishmania—subunit composition and mode of recruitment to different cap-binding complexes
Source: Nucleic Acids Res. 2015 Jun 19;43(13):6222–35. doi: 10.1093/nar/gkv564 (PMC4513851; doi:10.1093/nar/gkv564)

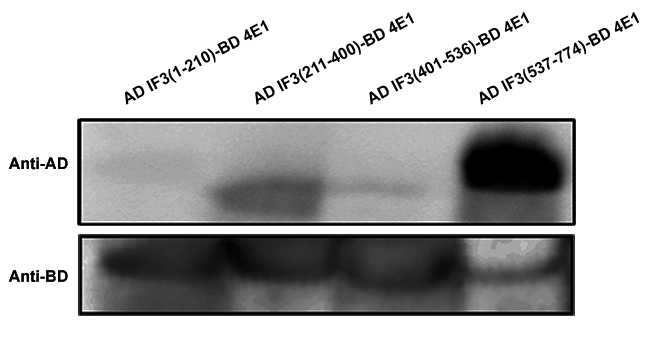

Supplement: SUPPLEMENTARY DATA [file supp_gkv564_nar-01173-v-2015-File015.tif]

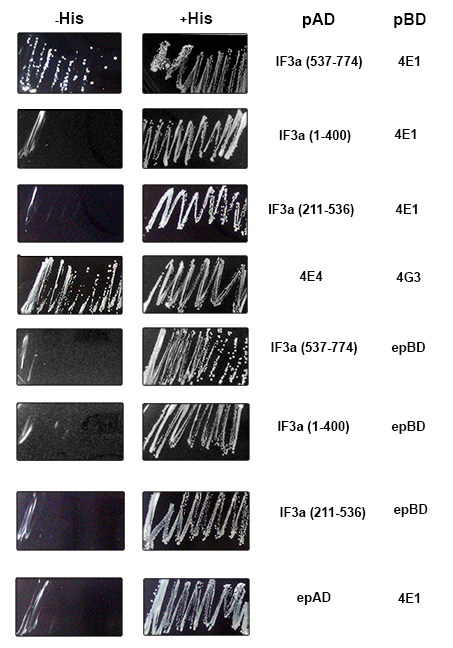

Supplement: SUPPLEMENTARY DATA [file supp_gkv564_nar-01173-v-2015-File016.tif]

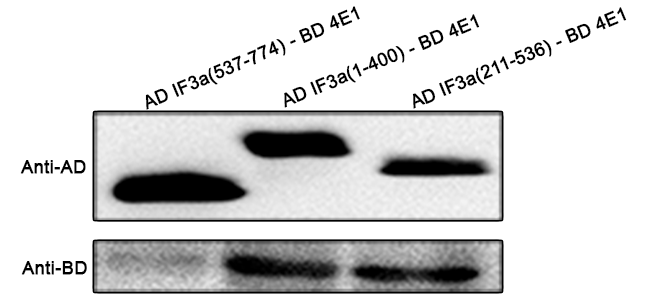

Supplement: SUPPLEMENTARY DATA [file supp_gkv564_nar-01173-v-2015-File017.tif]
